# Large-scale transcriptomic analyses reveal downstream target genes of ZFY1 and ZFY2 transcription factors in male germ cells

**DOI:** 10.1038/s41418-025-01569-6

**Published:** 2025-08-27

**Authors:** Hayden Holmlund, Manon Coulée, Yasuhiro Yamauchi, Benazir Yarbabaeva, Muhammetnur Tekayev, Isabella R. Garcia, Olivier U. Feudjio, Alberto de la Iglesia, Lee Larcombe, Peter J. I. Ellis, Julie Cocquet, Monika A. Ward

**Affiliations:** 1 Yanagimachi Institute for Biogenesis Research, John A. Burns School of Medicine, University of Hawaii, Honolulu, HI USA; 2 Institut Cochin, Université Paris Cité, INSERM, CNRS, Paris, France; 3 School of Biosciences, University of Kent, Canterbury, UK; 4 ADLIN Science, Evry, Cedex France; 5 Apexomic, Stevenage Bioscience Catalyst, Stevenage, UK

**Keywords:** Gene expression, Cell biology, Chromatin, Development

## Abstract

The mouse zinc finger genes *Zfy1* and *Zfy2* are essential for male fertility. Recently, we produced *Zfy1* knock-out (KO), *Zfy2* KO, and *Zfy1/2* double-knock-out (*Zfy* DKO) mice, and found that *Zfy* DKO males were infertile. The mechanism by which ZFY contributes to reproduction remains unknown but based on predicted protein sequence and in vitro assays we hypothesize that it controls expression of genes essential for spermatogenesis. To identify which genes ZFY regulates, we performed comparative transcriptome analysis of sorted male germ cells at three different spermatogenesis stages from three *Zfy* KO models and control wild-type males. Significantly altered germ cell transcriptomes were identified with *Zfy2* KO and *Zfy* DKO. Analyses of differentially expressed genes supported that *Zfy* loss altered spermatogenesis, DNA packaging/chromatin organization, and apoptosis pathways. Alternative splicing was deregulated in *Zfy* KO models, affecting sperm function and chromatin regulation pathways. In support of in-silico findings, *Zfy* DKO males were shown to have impaired post-meiotic chromatin remodeling and sperm chromatin organization, functional sperm deficiencies, and increased germ cell apoptosis. ZFY regulation of apoptotic pathways was demonstrated also in transfected human cells. We conclude that *Zfy* is a critical regulator of meiosis and spermiogenesis in addition to its previously described function as a cell-cycle regulator.

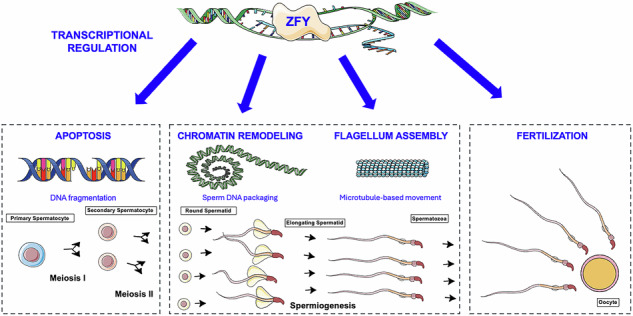

## Highlights

- Murine *Zfy* regulates expression and alternative splicing of hundreds of genes in male germline.
- *Zfy* loss alters pathways related to spermatogenesis, DNA packaging/chromatin organization, and apoptosis.
- Regulation of cell cycle and apoptosis pathways is conserved in human *ZFY*.

## Introduction

The zinc finger genes Y-linked (*ZFY*) are critical regulators of reproduction and cellular processes (reviewed by [1]). *ZFY* belongs to the broader *ZFX* family of genes that have been recently shown to act primarily as regulators of cell proliferation and general cellular functions [2]. Structurally, all family members comprise an N terminal acidic protein-binding region and a C terminal zinc-finger DNA-binding region, separated by a nuclear localization signal. However, while *ZFX* family genes are ubiquitously expressed in mammals, *ZFY* is unique in also expressing a shorter testis-specific splice variant ([3]; reviewed by [1]) with a reduced acidic domain. In humans, the full-length form is ubiquitously expressed, while in mice both short and long forms are testis specific, indicating that in this species ZFY is specialized for its role in spermatogenesis.

Although all *ZFX* family genes are believed to be transcriptional activators [3], the mechanism by which they control cellular processes is still under investigation. Deletion of *ZFX* and *ZNF711* in human female cultured cells (naturally lacking *ZFY*) strongly reduced cell division rates, and transcriptome analysis of these mutant lines revealed severe downregulation of cell cycle regulation pathways. Epigenetic analysis showed that *ZFY*, *ZFX*, and *ZNF711* bind to the majority of CpG island promoters, suggesting that all three proteins regulate global processes. It was proposed that the *ZFX* family genes share a similar function to the *MYC* family of oncogenic genes, which bind to thousands of promoters but primarily control cell proliferation [2, 4, 5]. Analysis of aneuploid cell lines with varying X and Y chromosome content has shown that the sex chromosomes modulate the expression of hundreds of autosomal genes, with *ZFX* and *ZFY* identified as major drivers of this both in vitro [6] and in vivo [7].

**Fig. 1:**
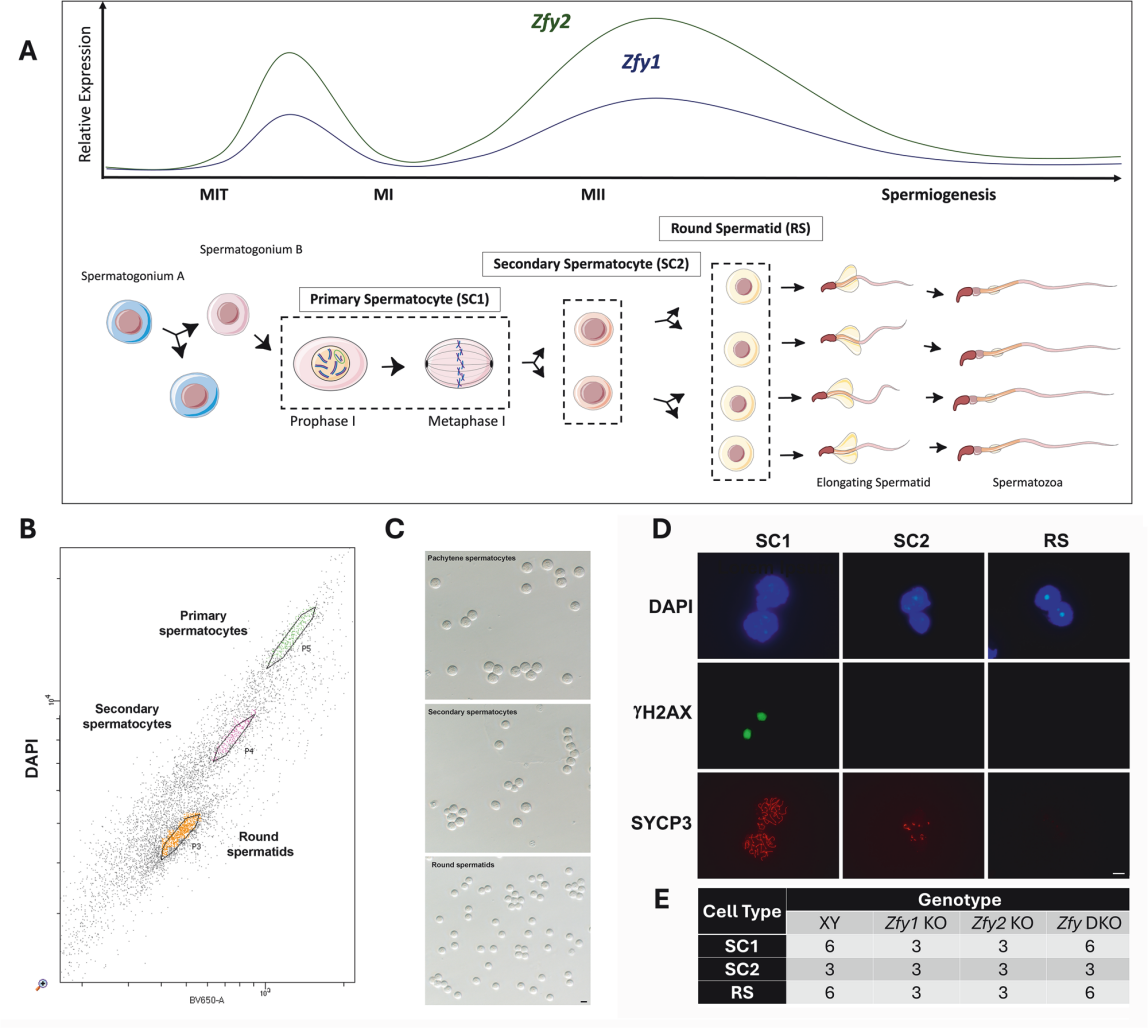
Isolation of male germ cells from *Zfy* KO mice. **A** Schematic diagram illustrating *Zfy* expression. Expression of both *Zfy* homologs in relation to mitosis (MIT), meiosis I and II (MI and MII), and spermiogenesis is represented by lines (*Zfy1* shown with dark blue, *Zfy2* shown with dark green). Relative expression levels shown are representative of published RNA-seq data [80–82]. The paternal and maternal chromosomes are shown in purple and blue, respectively, during prophase I and metaphase I. The Meiotic Sex Chromosome Inactivation (MSCI) sex body in pachytene spermatocytes is shown by green oval. This figure panel was adapted from Fig. 2 in [1]. **B** Example of FACs sorting showing populations of primary spermatocytes (SC1), secondary spermatocytes (SC2), and round spermatids (RS). **C** Phase contrast photo showing FACs-purified fractions of SC1, SC2, and RS. Scale, 10 µm. **D** Identification of male germ cells using DAPI, γH2AX, and SYCP3 immunostaining. Cells are differentiated based on morphology (DAPI) and presence or absence of γH2AX (visible only in primary pachytene spermatocytes) and SYCP3 (visible only in primary and to a lesser extent in secondary spermatocytes). Scale, 10 µm. **E** number of biological replicates used for RNA-seq. In the pilot experiment, 3 *Zfy* DKO and 3 XY males were used, each providing SC1 and RS cell types (SC2 were not collected). In the expanded experiment, 3 males of each genotype (*Zfy1* KO, *Zfy2* KO, *Zfy* DKO and XY) were used, each male providing three cell types (SC1, SC2, RS). The data from the pilot and the expanded experiment for matching genotypes and cell types were compared and were consistent. Thus, for all analyses, the data from both runs were analyzed together, with the number of biological replicates as shown.

Spermatogenesis is the process during which spermatozoa are formed (Fig. 1A). Briefly, germline stem cells (spermatogonia type A) proliferate mitotically, replenishing the stem cell pool and also giving rise to a daughter lineage (spermatogonia type B) committed to entering meiosis. On initiation of meiosis, they become primary spermatocytes which undergo two successive meiotic divisions: meiosis I produces secondary spermatocytes and meiosis II produces haploid round spermatids. Finally, round spermatids undergo spermiogenesis, a morphological differentiation process resulting in the final mature spermatozoa. Differentiated sex chromosomes (XX in females and XY in males) present specific challenges in the pachytene stage of meiosis I prophase, when genetic recombination occurs. Since the great majority of Y chromosome sequence is male-specific, it lacks homology to the X chromosome and does not pair. This triggers global transcriptional silencing of the X and Y chromosomes during pachytene, termed Meiotic Sex Chromosome Inactivation (MSCI) [8]. MSCI is a specialized form of a broader mechanism, which silences all chromosomal regions that fail to pair during meiosis. MSCI is essential for spermatogenesis and ensures proper meiotic development and sperm formation.

Mouse *Zfy* genes have been previously shown to have multiple functions during spermatogenesis. First, they promote MSCI [9] at the onset of meiosis I prophase, triggering their own silencing. Second, in spermatocytes that fail to correctly undergo MSCI, *Zfy* remains active throughout prophase, triggering apoptotic elimination of these aberrant cells [10, 11] and thus being termed a “meiotic executioner”. Thirdly, *Zfy* promotes meiosis II via unknown mechanisms [12], and fourthly, it is essential for spermiogenesis [13–15]. *Zfy’s* role as a meiotic executioner—for which the strongest evidence comes from mice [9, 10], but also horses [16, 17], pigs [18] and rodents that have lost Y chromosome [19, 20]—is proposed to have broader evolutionary consequences. In general, Y-linked genes are subject to evolutionary pressures that lead either to loss of function, or translocation to other genomic locations—this explains why the Y chromosome is small and gene poor (reviewed in [21]). However, *ZFY* is essential for male fertility (i.e., it cannot be lost), and *must* remain subject to meiotic silencing to avoid triggering its executioner function (i.e., it cannot be relocated). This combination of properties gives rise to the “Persistent Y” hypothesis, in which the presence of ZFY explains why the mammalian Y chromosome is seemingly more evolutionarily resilient than Y chromosomes in other taxa, where sex determination mechanisms are more labile and Y chromosomes may be eliminated from the population entirely [21, 22].

The mouse has two *Zfy* genes, *Zfy1* and *Zfy2*, both present as single copies on the Y chromosome short arm. They encode for the isoforms ZFY1 and ZFY2, which are highly similar and are putative transcription factors. Transgene-rescue experiments have shown that these *Zfy* genes are critical for male fertility and normal sperm production [3, 9–13, 15, 23] reviewed by [1]). Recently, we confirmed the importance of *Zfy* by creating *Zfy1* knock-out (KO), *Zfy2* KO, and *Zfy1/2* double knock-out (DKO) mice [14]. The *Zfy1* KO males were fertile with minor abnormalities in spermatogenesis, while the *Zfy2* KO males were sub-fertile, and the *Zfy* DKO males were completely infertile, producing poor quality dysfunctional sperm [14].

Here, we report on using these *Zfy* KO mouse models to investigate the function of ZFY in vivo. We collected three types of male germ cells, in which *Zfy1* and *Zfy2* are expressed and expected to play roles, primary spermatocytes (SC1), secondary spermatocytes (SC2), and round spermatids (RS), isolated from *Zfy1* KO, *Zfy2* KO, and *Zfy* DKO males, and performed RNA sequencing analyses on 48 different samples. Significantly altered transcriptomes were observed for all germ cell types from *Zfy2* KO and *Zfy* DKO males. In-depth analyses of differentially expressed genes (DEGs) supported that loss of *Zfy* altered pathways relating to spermatogenesis, DNA packaging/chromatin organization, and apoptosis. Alternative splicing was also found deregulated in *Zfy* KO mouse models, affecting pathways relating to sperm function and chromatin regulation. The in-silico findings were confirmed by showing DNA damage in testicular male germ cells, defects in post-meiotic chromatin remodeling and abnormal chromatin packaging in sperm from *Zfy* DKO males, and severely impaired sperm function in vitro with *Zfy2* KO males. The data provide insights into *Zfy* roles in male reproduction and guide ongoing attempts to discover the roles of the human *ZFY* ortholog in vivo.

## Results

### Large-scale RNA sequencing analyses of the three Zfy KO models show that ZFY1 and ZFY2 regulate expression of thousands of genes in meiotic and postmeiotic cells

**Fig. 2:**
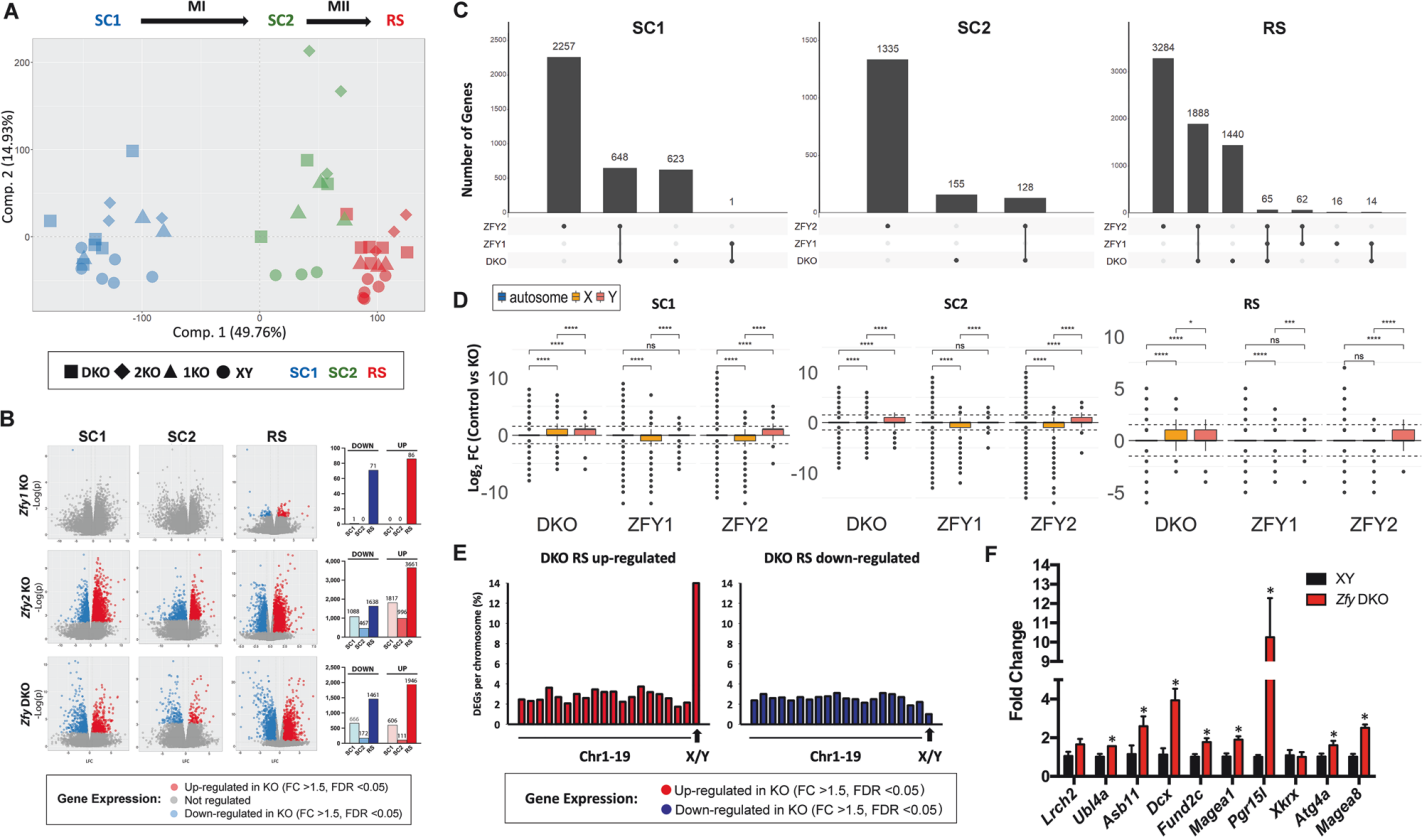
Significantly altered transcriptomes were observed for *Zfy2* KO and *Zfy* DKO germ cells. **A** Principal component analysis (PCA) comparing transcriptomes of XY, *Zfy1* KO, *Zfy2* KO, and *Zfy* DKO primary spermatocytes (SC1), secondary spermatocytes (SC2), and round spermatids (RS). Samples clustered according to the cell type before genotype, with SC2 samples being closer to RS than to SC1. The code for cell type and genotype is as indicated. MI = Meiosis 1; MII = Meiosis 2; 1KO, *Zfy1* KO; 2KO, *Zfy2* KO; DKO, *Zfy* DKO; and XY, wild-type male of same genetic background. **B** Volcano plots and bar graphs showing the differentially expressed genes (DEGs) identified by RNA-seq for SC1, SC2, and RS, *Zfy1* KO, *Zfy2* KO, or *Zfy* DKO vs XY. FC = fold change, −log(p) = −log_2_ of FDR-adjusted *p* value, LFC = log_2_ fold change. **C** UpSet plot showing overlap (shown as lines connecting filled cells) of DEGs (FC > 1.5, FDR < 0.05) for all KO models and cell types. **D** Boxplot distributions showing the mean Log_2_ FC of autosomal genes compared to X and Y genes. Significant increases were observed between X or Y genes compared to autosomes in all cell types from DKO, while downregulation of X genes was observed in SC1 and SC2 from *Zfy1* KO and *Zfy2* KO, and in RS from *Zfy1* KO. Box: 25th/75th percentiles. Bar in the box: median. Whiskers: 1.5 times the interquartile range from the 25th/75th percentiles. Dashed lines: 1.5-fold change. ns: not significant. Stars indicate *p* values calculated using Wilcoxon test adjusted with Benjamini-Hochberg correction (*: *p* < 0.05, **: *p* < 0.005, ****: *p* < 0.00005, ns: non-significant. **E** *Zfy* DKO up-regulated (red) and down-regulated (blue) DEGs (FC > 1.5, FDR < 0.05) listed by chromosome for DKO RS cells. Percentage of DEGs per total gene number on each chromosome (mouse autosomal chromosomes, Chr1-19, and sex chromosomes, XY. **F** qPCR of sex-linked genes in XY (black) and *Zfy* DKO (red) whole testes, with the geometric mean of *Ppia*, *Rsp18*, and *Rplp0* used as a reference. Graphs are *n* = 3 ± SEM. Significant changes (*p* < 0.05) are shown with an asterisk.

To be able to study and compare *Zfy1* KO, *Zfy2* KO and *Zfy* DKO models, we first backcrossed them to C57BL/6 for at least 10 generations using breeding and assisted reproduction since *Zfy* DKO are infertile. The currently proposed functions of mouse *Zfy* include a role in MSCI and apoptosis in primary spermatocytes, completion of meiosis in secondary spermatocytes, and chromatin remodeling, spermatid elongation, and flagellum formation in round spermatids (Fig. 1A) ([3] and reviewed by [1]). We aimed to identify the molecular consequences of *Zfy1* and/or *Zfy2* loss by looking at the spermatogenesis stages where these phenotypes manifest in the testis in the three *Zfy* KO models. We focused our analysis on three key germ cell stages: meiotic primary spermatocytes (SC1), meiotic secondary spermatocytes (SC2), and post-meiotic round spermatids (RS) (Fig. 1A). SC1, SC2, and RS, were isolated from XY, *Zfy1* KO, *Zfy2* KO, and *Zfy* DKO males with a purity of at least 90% using FACs sorting (Fig. 1B–D) and bulk RNA-seq analyses were performed on these cell populations. Altogether, the analysis of transcriptome data was performed using 48 different samples originating from 3 different germ cell types and 4 different mouse genotypes, for a total of 1.6 billion reads (Fig. 1E, Fig. S1, Table S1). Principal component analysis (PCA) showed that most significant transcriptional changes were due to the cell type, with SC2 transcriptome being closer to RS than to SC1 (Fig. 2A and S2A, B). Substantial changes in gene expression were also observed for *Zfy2* KO and *Zfy* DKO compared to XY samples, and more subtle transcriptional changes were observed for *Zfy1* KO samples (Fig. 2A; Fig. S2A, B).

Differential expression analyses comparing KO and XY samples within each germ cell types (fold change, FC > 1.5, FDR < 0.05) showed that for *Zfy* DKO, 1272 differentially regulated genes (DEGs) were detected for SC1, 283 for SC2, and 3407 for RS (Fig. 2B; Fig. S2C). A larger number of DEGs was observed for *Zfy2* KO for all three cell types, with 2905 for SC1, 1463 for SC2, and 5299 for RS (Fig. 2B; Fig. S2C). Only a handful of DEGs were observed in *Zfy1* KO, with 1 for SC1, 0 for SC2, and 157 for RS (Fig. 2B; Fig. S2C). Except for *Zfy* DKO SC1 and SC2, more DEGs were upregulated rather than downregulated (Fig. 2B). To detect minimal transcriptional changes in *Zfy1* KO, we also performed a less stringent analysis (FC > 1.5, *p* < 0.05) to detect DEGs “approaching” significant de-regulation. For *Zfy1* KO, 1415 deregulated genes were detected for SC1, 1009 for SC2, and 2024 for RS (Fig. S2C). For *Zfy2* KO and *Zfy* DKO, thousands of deregulated genes were detected for all three cell types, with larger transcriptional changes observed in RS compared to SC1 and SC2.

Overall, these data support that the loss of *Zfy2*, and both *Zfy1* and *Zfy2*, leads to extensive transcriptomic changes, while the loss of *Zfy1* alone causes only minor gene expression changes.

### *Zfy2* KO and *Zfy* DKO have both common and unique sets of DEGs

We compared the DEGs found in all three models (FC > 1.5, FDR < 0.05) and found that many DEGs detected in *Zfy2* KO were unique and not present in *Zfy* DKO (Fig. 2C). To assess the correlation in transcriptional changes between the three *Zfy* KO models, we performed scatterplot analysis of DEGs (Fig. S3). Overall, the correlation was higher between *Zfy* DKO and *Zfy2* KO than between *Zfy2* DKO and *Zfy1* KO in SC1 and RS. However, the correlation between *Zfy1* KO and *Zfy2* KO was higher than when *Zfy1* KO and *Zfy2* KO were compared with *Zfy* DKO in DEGs downregulated in SC2 (*r* = 0.90 vs. 0.40, Fig. S3Biv and *r* = 0.53 vs. 0.30, Fig. S3Bvi). Since ZFY protein belongs to ZFX family [2], we also specifically looked whether other *Zfx* related genes are deregulated to identify potential compensation mechanisms. Interestingly, we found that *Zfx* is downregulated in SC1 and SC2 from *Zfy2* KO (FC > 1.5, *p* < 0.05, not adjusted), while *Zfp711* is significantly upregulated in *Zfy* DKO RS.

### Loss of both *Zfy1* and *Zfy2* deregulates sex chromosome-encoded genes

We next examined the distribution of DEGs across chromosomes. The mean Log_2_ fold change of all X and Y-encoded genes compared to all autosomal genes was calculated and confirmed a global upregulation of X and Y genes in *Zfy* DKO SC1, SC2 and RS cells (Fig. 2D). When considering the total number of de-regulated X/Y genes, a significant number (15.3%) of all sex-linked genes were up-regulated (FC > 1.5, FDR < 0.05) in *Zfy* DKO RS cells, whereas only a small number (1.0%) of sex-linked genes were down-regulated (Fig. 2E). Among genes approaching significant de-regulation (FC > 1.5, *p* < 0.05), many up-regulated sex-linked genes were identified for SC1 and SC2 as well as for RS (Fig. S4A). Upregulation of individual sex-linked genes was also detected with RT-qPCR in *Zfy* DKO testes for eight out of ten genes that were selected from the pilot RNA-seq DEGs (Fig. 2F) and in purified round spermatids from *Zfy* DKO males (Fig. S5A).

We looked at the expression dynamics of these X/Y-linked DEGs throughout wild-type meiosis and MSCI to determine if they are expressed before MSCI or only after, in RS. We found that a majority of X/Y upregulated genes in *Zfy* DKO RS are predominantly RS-expressed genes (i.e., not or poorly expressed in primary spermatocytes) while ~a third is expressed in spermatogonia and in early spermatocytes (at the leptotene stage). This category of genes is shut down at the pachytene stage by MSCI and is not reactivated in RS (Fig. S6). X/Y-linked upregulated genes in *Zfy* DKO SC1 had similar dynamics (i.e., a mix of RS-enriched genes and MSCI shut down genes) indicating that *Zfy* DKO germ cells fail to silence X/Y gene expression at the pachytene stage but also leads to premature expression of RS-enriched genes (Fig. S6).

Surprisingly, global downregulation of X-linked genes was observed for *Zfy2* KO SC1 and SC2, as well as for *Zfy1* KO SC1, SC2, and RS (Fig. 2D). When considering the percentage of sex-linked genes downregulated, no noticeable trend was observed in *Zfy2* KO germ cells (Fig. S4B). However, for *Zfy1* KO, several sex-linked genes approaching significant downregulation (FC > 1.5, *p* < 0.05) were observed in SC1 and SC2 (respectively, 88 DEG for SC1 and 66 for SC2) (Fig. S4C). We measured expression for eight of these genes (*Slc16a2*, *Ids*, *Nhs*, *Hdac6*, *Acsl4*, *Tspyl2*, *Tsr2*, and *Diaph2*) in whole testes from all genotypes, and did not observe any significant changes in expression except for slight downregulation of *Diaph2* in *Zfy2* KO testis (Fig. S7). The downregulation of *Zfy1* KO sex-linked genes may reflect more subtle transcriptional changes specific to germ cells.

Together, these data show that loss of *Zfy* results in de-regulation of sex-linked genes in meiotic and postmeiotic cells.

### Loss of *Zfy2* and both *Zfy1* and *Zfy2* leads to upregulation of chromatin-related pathways in round spermatids

To identify biological functions of genes that are deregulated in *Zfy* KO mice, we performed pathway enrichment analysis (PEA) and gene set enrichment analysis (GSEA). PEA identifies which pathways are enriched among DEGs but also considers non-regulated genes to account for bias [24]. GSEA provides complementary results as it finds overrepresented gene sets by considering all genes in relation to their expression level [25]. Additionally, gene ontology (GO) analysis was also performed to identify overexpression GO terms. A complete summary of all data is shown in Tables S2–S8.

**Fig. 3:**
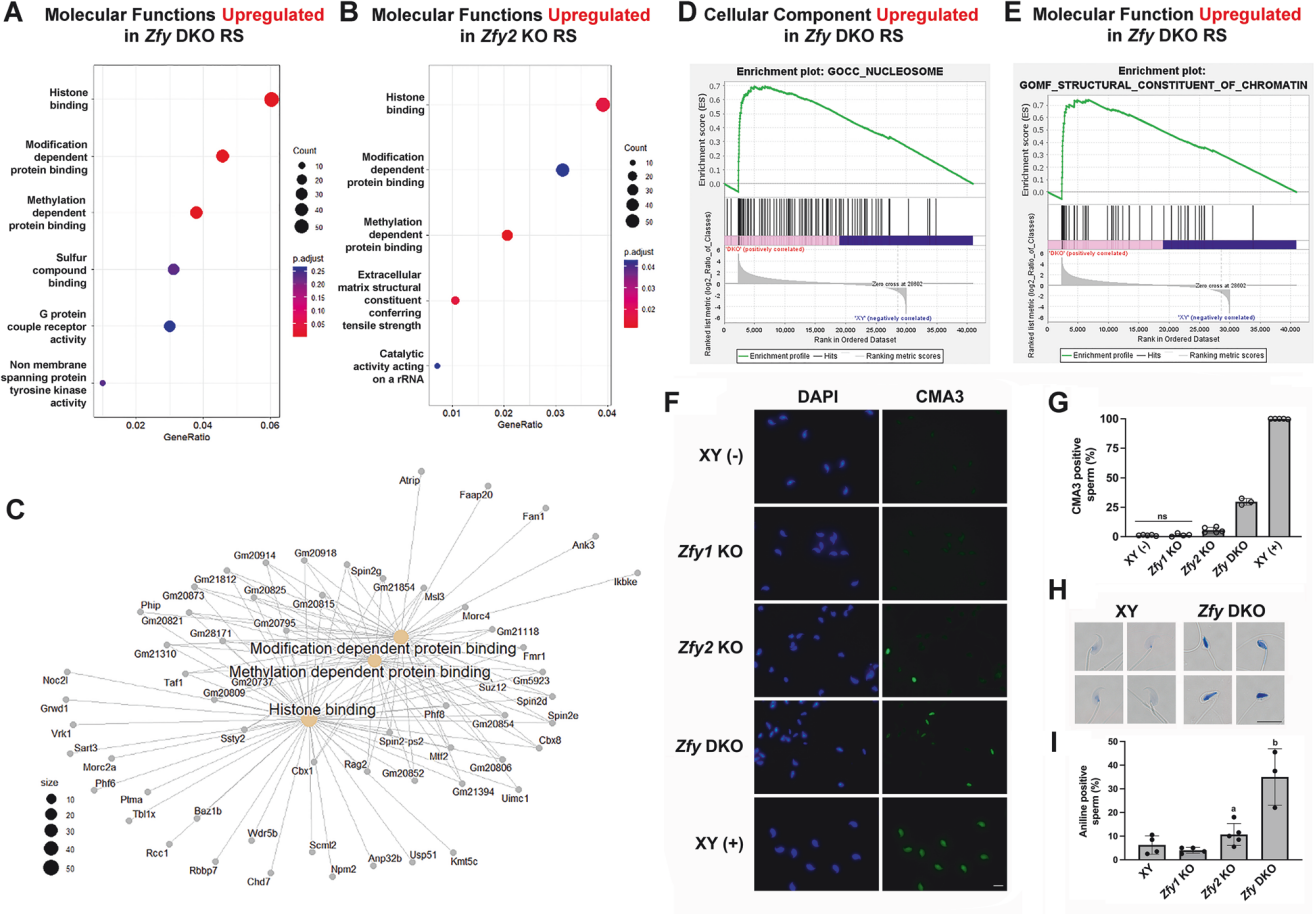
Chromatin-related pathways are upregulated in *Zfy* KO round spermatids with consequences for sperm chromatin packaging. Dot plots for **A** *Zfy* DKO and **B** *Zfy2* KO showing upregulated Molecular Functions in RS DEGs (FC > 1.5, FDR < 0.05). The EnrichR function in ClusterProfiler was used to detect enriched pathways for *Zfy* KO DEGs. **C** CNET plot generated from ClusterProfiler, showing the gene composition of each Molecular Function pathway upregulated in *Zfy* DKO RS. **D**, **E** Gene set enrichment analysis (GSEA) results showing significant enrichment (FDR < 0.05) of DNA-packaging gene sets in *Zfy* DKO RS. For each pathway, enrichment plots depict graphical view of the enrichment score of each gene. **F**–**I** Chromatin deficiencies in *Zfy* KO sperm. **F** Exemplary images of CMA3 stained sperm from *Zfy1* KO, *Zfy2* KO and *Zfy* DKO males. XY (-) are sperm from wild-type males (negative control) and XY (+) are sperm from wild-type males treated with 2 mM DTT and 0.5% Triton to destabilize sperm chromatin (positive control). Scale, 10 µm. **G** Percentage of CMA3 positive sperm for each genotype. Data are shown as average ±SDev, with replicate data as empty circles, and with *n* = 5 for XY(+), XY(-) and *Zfy2* KO, *n* = 4 for *Zfy1* KO, and *n* = 3 for *Zfy* DKO. For each male 200 sperm were analyzed and scored as either positive or negative. Statistical analysis (*t*-test performed on angles): each group different from each other except XY(-) and *Zfy1* KO. H: Exemplary images of wild-type (XY) and *Zfy* DKO sperm heads stained with aniline blue. Scale, 10 µm. **I** Percentage of aniline positive sperm for each genotype. Data are shown as average ±SDev, with replicate data as filled circles, and with *n* = 5 for *Zfy2* KO, *n* = 4 for XY and *Zfy1* KO, and *n* = 3 for *Zfy* DKO males. For each male 200 sperm were analyzed and scored as either positive (dark blue) or negative (light blue or clear). Statistical analysis (*t*-test performed on angles): ^a^different than *Zfy1* KO; ^b^different than all other.

During spermiogenesis, as round spermatids transform into sperm, a major chromatin reorganization takes place which results in the replacement of most histones by protamines and in the extreme compaction of the sperm genome [26]. Here, using both PEA, GO analysis, and GSEA, we observed upregulation of DNA packaging/chromatin pathways in round spermatids from *Zfy* KO males. PEA and GO analysis revealed that chromatin-remodeling and nucleosome processes were enriched in upregulated *Zfy2* KO and *Zfy* DKO RS DEGs (FC > 1.5, FDR < 0.05) (Fig. 3A–C and Tables S2 and S3). PEA using ClusterProfiler showed that “histone binding” ranked among the three upregulated molecular functions for both *Zfy* DKO (Fig. 3A) and *Zfy2* KO (Fig. 3B) RS, and gene network plot analysis revealed that histone binding pathways were clustered closely with modification and methylation-dependent protein binding pathways (Fig. 3C). GSEA found that two out of four significantly upregulated pathways in *Zfy* DKO RS were related to chromatin structure: GOMF-STRUCTURAL-CONSTITUENT-OF-CHROMATIN and GOCC-NUCLEOSOME (Fig. 3D, E, Table S4). Only two upregulated pathways that were not related to DNA-packaging were identified for *Zfy2* KO RS (GOMF-TRACE-AMINE-RECEPTOR-ACTIVITY and GOMF-TYPE I- INTERFERON-RECEPTOR-BINDING; Table S4). Upregulation of individual genes from chromatin-related pathways was also detected with RT-qPCR in purified round spermatids from *Zfy* DKO males (Fig. S5B).

We next performed motif enrichment analysis (MEA) to find potential co-regulators via the identification of binding motifs enriched in the sequence of each DEG group [27]. For each identified motif we attempted to produce a viable model of ZFY binding to the motif sequence in presence of a zinc cofactor using Alphafold3 modeling [28]. MEA detected two significantly enriched motifs for upregulated *Zfy* DKO DEGs, with the human genes *HOXC12* and *HMBOX1* predicted to be the top transcription factor matches for each motif (Fig. S8A). The mouse ortholog of *HMBOX1*, *Hmbox1*, is expressed at low levels in round spermatids (Fig. S8B) although its function in male germ cell DNA packaging is unknown. The mouse ortholog of *HOXC12* is not expressed in the male germline (Fig. S8B). However, another gene from the *HOXC* cluster, *Hoxc9*, is expressed strongly in round spermatids and was also identified as a potential match for this motif (Fig. S9A, B). With this motif, we were able to produce a stronger in-silico modeling for ZFY1 and ZFY2 binding to this motif in the presence of a zinc co-factor than for the other motifs (Fig. S9C, D). This modeling was shown to have a higher degree of confidence than a previously identified motif for human ZFX [2] (Fig. S9E) and was used to visualize ZFY interaction with the motif DNA helix (Fig. S9F).

Six motifs were significantly enriched in *Zfy2* KO upregulated DEGs (Fig. S8C), five of which have gene candidates with mouse orthologs expressed in round spermatid: *Zfp105*, *Zfp809*, *Tead2*, *Sox17*, and *Elf1* (Fig. S8D). For *Zfy1* KO RS, PEA did not identify any enriched pathway. GO analysis identified pathways related to DNA replication and DNA binding, but none directly related to chromatin reorganization, as significantly upregulated (File S1). GSEA did not find upregulated gene sets in *Zfy1* KO RS, and MEA did not identify any significantly enriched motifs among downregulated *Zfy1* KO RS DEGs.

These findings suggest that loss of both *Zfy1* and *Zfy2*, and potentially loss of *Zfy2* alone, causes changes in expression of genes that could be involved in regulation of chromatin reorganization during spermatogenesis.

### Loss of *Zfy2* or of both *Zfy1* and *Zfy2* leads to defects in post-meiotic chromatin remodeling and abnormal chromatin packaging in epididymal sperm

During spermiogenesis, spermatid chromatin undergoes specific reorganization and compaction as most histones are removed and replaced with protamines. Spermatozoa are expected to have compact, protamine-bound chromatin. Because our data indicated alterations in expression of genes involved in chromatin reorganization resulting from *Zfy* loss, we tested chromatin compaction in cauda epididymal sperm from *Zfy1* KO, *Zfy2* KO, and *Zfy* DKO males, and compared to sperm from wild-type XY controls. Two assays were employed, chromomycin-A3 (CMA3) staining and aniline blue staining. CMA3 is a fluorochrome that binds to the minor groove in DNA. Sperm heads that stain positive for CMA3 have protamine deficiencies and improperly condensed chromatin [29, 30]. *Zfy* DKO and *Zfy2* KO males produced higher number of CMA3 positive sperm than controls (Fig. 3F, G). Aniline blue stains the positive lysine residue on histones; positive aniline staining on sperm suggests histone retention and inefficient histone-to protamine transition [31, 32]. More than one-third of sperm from *Zfy* DKO spermatozoa stained positive for aniline blue (Fig. 3H, I). *Zfy2* KO males had more aniline blue positive sperm when compared to *Zfy1* KO but not when compared to XY (Fig. 3I). Sperm from *Zfy1* KO males did not differ from XY in either test (Fig. 3F–I).

**Fig. 4:**
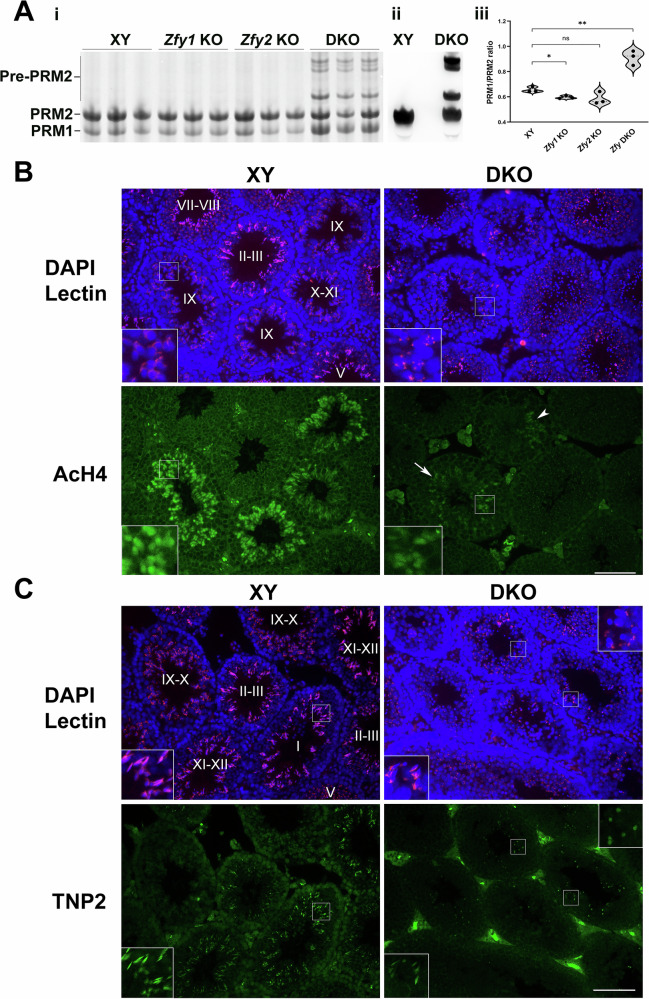
*Zfy* loss leads to defects of post-meiotic chromatin remodeling. **A** Protamine content in sperm from *Zfy* KO males. **i** Coomassie blue-stained acid urea polyacrylamide gel electrophoresis (AU-PAGE) of cauda epididymal sperm basic nuclear protein extracts from XY, *Zfy1* KO, *Zfy2* KO and *Zfy* DKO (DKO) males (*n* = 3 per genotype). PRM1, protamine 1; PRM2, protamine 2, pre-PRM2, precursor forms of PRM2. **ii** Acidic Western blot using anti-PRM2 antibody confirming identity of PRM2 and pre-PRM2. **iii** Quantification of PRM1/PRM2 ratio related to (**A**). PRM1/PRM2 ratios are expressed in arbitrary units. Statistical analysis (unpaired *t*-test): *P < 0.05; **P < 0.01. **B**, **C** Immunofluorescence detection of proteins involved in chromatin remodeling in testis sections from wild-type (XY) and *Zfy* DKO (DKO) males. Hoechst (blue) was used to stain nuclei and Lectin (red) was used to stain acrosome. In DKO, spermatogenesis is severely disturbed, and tubules could not be staged. Only few elongated spermatids could be seen, and these were morphologically abnormal. Punctuated and dispersed lectin pattern indicated disturbances of acrosome development. Insets, 3x magnification. Scale, 100 µm. **B** Immunofluorescence detection of acetylated histone AcH4 (green). In XY, acH4 first appeared and was strongly expressed in the elongating spermatids St9 (stage IX), expression weakened in elongating spermatids St10-11 and was not seen in more advanced spermatids St12-16. In DKO, AcH4 expression was weaker (arrow, inset) and patchy (arrowhead). **C** Immunofluorescence detection of TNP2 (green). In XY, TNP2 expression was first seen in elongating spermatids St11-12 (stage XI-XII), appeared the strongest in St13 (stage I), decreased in St14 (stage II-III), and was no longer seen in St15. TNP2 was not seen in round spermatids (St1-8, stages I to VIII) and early elongating spermatids (St9-10; stage IX-X). In DKO TNP2 expression was observed in elongated spermatids but seemed weaker than in XY (XY vs. DKO, bottom insets). Strong green signal was also seen in highly condensed, roundish cells, which were also strongly stained with DAPI; these are presumed dead germ cells (DKO, top inset).

Sperm chromatin is bound by protamines (PRMs) and presence of histones is limited. The ratio between PRM1 and PRM2 is constant in mature sperm [33, 34] and its alterations are known to be associated with sperm defects and infertility (reviewed in [35, 36]). We examined protamine content in sperm from *Zfy* DKO males and controls. Concomitant to the striking accumulation of pre-PRM2 bands exclusively present in *Zfy* DKO males (Fig. 4Ai-ii, Fig. S10), PRM1/PRM2 ratio was altered in *Zfy* DKO showing a decrease of PRM2 relative to PRM1 (Fig. 4Aiii, *P* < 0.01). *Zfy1* KO showed a significant but not very strong decrease of PRM1/PRM2 ratio (*P* < 0.05) when compared to XY while *Zfy2* KO showed a decrease that did not reach significance (*P* = 0.08). The median PRM1/PRM2 was lower in *Zfy2* KO than *Zfy1* KO, which would indicate stronger chromatin defects. The lack of significance is likely due to the variability between three *Zfy2* KO samples.

Histone variants and transition proteins (TNPs) are transiently expressed in spermatids and are essential for correct reorganization and compaction of sperm genome with protamines. Histone H4 becomes highly acetylated (AcH4) in spermatids and this hyperacetylation is a crucial step that precedes and facilitates replacement of histones with histone variants, transition proteins and protamines (reviewed in [37]). Alterations in expression of genes involved in chromatin reorganization resulting from *Zfy* loss suggested that post-meiotic chromatin remodeling process is altered in *Zfy* KO males. By immunofluorescence on testicular sections, we observed that both AcH4 and TNPs signals were weaker and patchy in *Zfy* DKO testes compared to the specific and dynamic pattern observed in XY testes (Fig. 4B, C).

Together, the data support that sperm from *Zfy* DKO and *Zfy2* KO have poorly packaged chromatin likely resulting from impairments in histone-to-protamine exchange during spermiogenesis.

### Loss of *Zfy* leads to downregulation of spermatogenesis-related pathways in male germ cells

**Fig. 5:**
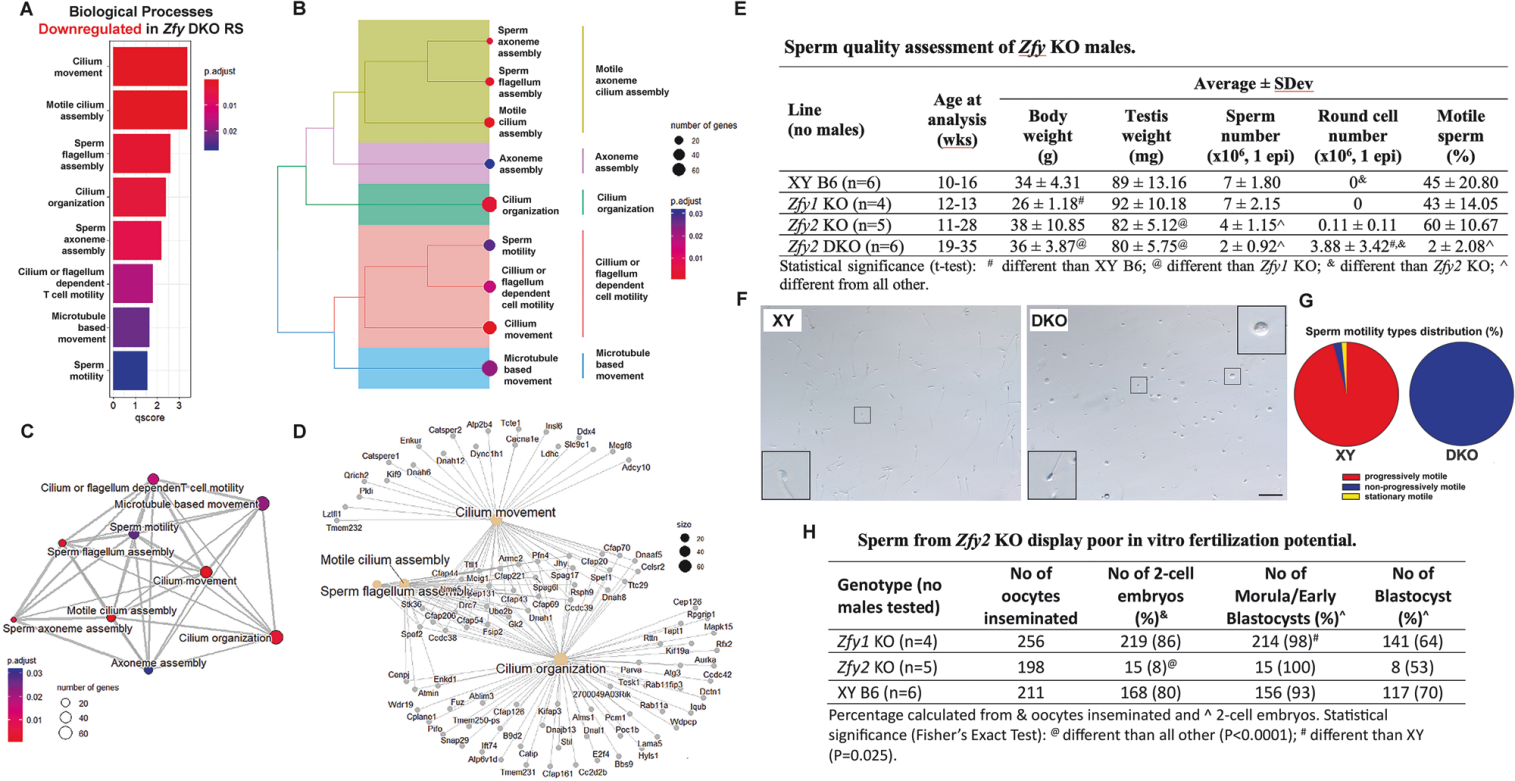
Spermatogenesis-related pathways are downregulated in round spermatids from *Zfy* KO with consequences for sperm quality and function in vitro. **A**–**D** The EnrichR function in ClusterProfiler was used to detect enriched biological processes for DEGs (fold change > 1, FDR < 0.05) downregulated in *Zfy* DKO RS. **A** Bar graph showing all significantly downregulated biological processes in *Zfy* DKO RS. *p* adjust = adjusted *p* value. **B** Tree plot showing the phylogenetic relationship between downregulated biological processes. **C** Enrichment map of downregulated biological processes. **D** Gene-concept network showing the gene composition and overlap of the top four downregulated biological processes. **E**–**H** Sperm quality and function. **E** Sperm quality assessment for wild-type control (XY), *Zfy1* KO, *Zfy2* KO and *Zfy* DKO males. **F** Exemplary phase contrast image of live sperm from XY control and *Zfy* DKO male. XY sample shows morphologically normal sperm (inset) that appear with high density while *Zfy* DKO sample shows few severely abnormal sperm (bottom inset) and many round cells (top inset). Insets, x3 magnification. Scale, 50 µm. **G** Sperm motility type distribution. Progressively motile sperm move fast following a linear trajectory; non-progressively motile sperm change location but move slowly, often in circles and do not progress far in distance; stationary motile sperm do not change location but display tail beating or twitching head movement. The data are average of 3 XY and 3 *Zfy* DKO males tested. **H** The results of in vitro fertilization performed with XY, *Zfy1* KO and *Zfy2* KO sperm. IVF data with *Zfy* DKO was reported before; no fertilization was observed under any conditions tested [14].

As round spermatids differentiate into mature spermatozoa, they undergo a series of morphological changes during which organelles are remodeled, and sperm head/tail development commences. Previously, we have shown that *Zfy* genes are required for normal spermiogenesis progression [13, 15]. Surprisingly, reproduction pathways were not among the most strongly deregulated pathways in *Zfy* KO RS (Table S4). To determine if ZFY regulates spermiogenesis via subtle transcriptional changes, we reduced the fold change threshold and performed PEA using all significantly deregulated genes regardless of expression value. These analyses revealed that processes related to sperm motility, flagellum assembly, cilium organization, manchette, and axoneme assembly were enriched in downregulated *Zfy* DKO RS DEGs (Fig. 5A–D; Table S5 and S6). Bar plot analysis confirmed significant enrichment of spermiogenesis and motility related pathways (Fig. 5A). Phylogenetic analysis of enriched pathways showed that the “assembly” pathways (sperm flagellum, axoneme, and motile cilium) were clustered separately from the “motility” pathways (sperm, cilium movement, microtubule based, flagellum-dependent) (Fig. 5B). However, analysis with enrichment map and gene concept networks confirmed that these spermiogenesis pathways are still interrelated and share many common genes (Fig. 5C, D). Downregulation of sperm motility pathways was not observed when performing GSEA (Table S2). Upregulation of individual genes from spermatogenesis-related pathways was also detected with RT-qPCR in purified round spermatids from *Zfy* DKO males (Fig. S5C).

To identify potential ZFY co-regulators, we used MEA to detect enriched motifs among *Zfy* DKO RS downregulated DEGs. We found three significantly enriched motifs, with *Meis1* (*Mus Musculus*), *POU3F4* (*Homo Sapiens*) and *ZNF35* (*Homo Sapiens*) identified as the top transcription factor candidates for these three motifs (Fig. S8E). Like *Zfy2*, both *ZNF35* gene and its mouse ortholog, *Zfp105*, are expressed in round spermatids (Fig. S8E). *Meis1* is only expressed at low levels in the male germline, and *Pou3f4* is not expressed significantly in the male germline (Fig. S8F).

For *Zfy2* KO RS, GO analysis found downregulated pathways that were related to general cellular function and not spermatogenesis (Table S7), and GSEA identified only one downregulated gene set (GOMF-PHEROMONE-ACTIVITY, Table S4). MEA found five motifs significantly enriched in *Zfy2* KO downregulated DEGs, and identified *Hnf4a* (*Mus Musculus*), *Prdm15* (*Mus Musculus*), *PAX5* (*Homo Sapiens*), *Zbtb14* (*Mus Musculus*), and *ETS* (*Homo Sapiens*) as the top transcription factor candidates for these motifs (Fig. S8G). Neither *Hnf4a* nor *Zbtb14* are expressed significantly in RS (Fig. S8H). *Prdm15*, as well as *Pax5* and *Ets1* (mouse homologs for *PAX5* and *ETS*, respectively) are expressed only at low levels in RS and do not have annotated functions in spermatogenesis (Fig. S8H).

For *Zfy1* KO RS, PEA did not identify any downregulated pathways, and GO analysis revealed that only non-reproduction pathways were downregulated (File S1). GSEA found just four downregulated gene sets: GOBP-RESPONSE-TO-PHENYL-ALANINE, GOMF-TRIPLET-CODON-AMINO-ACID-ADAPTOR-ACTIVITY, GOMF-PHEROMONE-ACTIVITY, and GOMF-PORE-FORMING-ACTIVITY (Table S4). MEA failed to identify any significant among downregulated *Zfy1* KO DEGs.

Collectively, these data suggest that loss of both *Zfy* homologs results in downregulation of spermiogenesis-related pathways in round spermatids but losing a single *Zfy* gene does not produce this effect.

### Sperm from *Zfy* DKO males are poorly motile and sperm from *Zfy2* KO males display poor in vitro fertilization potential

The deregulation of spermiogenesis pathways was only observed with *Zfy* DKO RS. This matches with our prior report showing that *Zfy* DKO males are completely infertile and have severe spermatogenesis defects [14]. Although we did not observe deregulation of spermatogenesis pathways with single *Zfy* KO mice, prior phenotype characterization showed that *Zfy1* KO males were fertile, while loss of *Zfy2* led to some fertility impairment evidenced as decreased litter size, decreased testis size and increased incidence of morphologically abnormal sperm [14]. Since our first characterization of *Zfy* KO mice [14] *Zfy2* KO and *Zfy* DKO males were backcrossed to C57BL/6 genetic background and we therefore revisited the spermiogenic phenotype for all *Zfy* KO models. Both *Zfy2* KO and *Zfy* DKO males had decreased testis size, decreased sperm number, and sloughed round cells in cauda epididymides, with *Zfy2* KO phenotype less severe than that of *Zfy* DKO (Fig. 5E). Sperm from *Zfy* DKO males had drastically low total sperm motility (Fig. 5E, F). Moreover, quantification of specific motility types revealed that all sperm from *Zfy* DKO males displayed non-progressive motility while most sperm from wild-type XY males moved progressively (Fig. 5G, Movies M1 and M2). Finally, when assessing sperm function, we observed a severe impairment in sperm ability to fertilize oocyte in vitro for *Zfy2* KO males, and not for *Zfy1* KO males (Fig. 5H); *Zfy* DKO males were not tested as their sperm have been shown before to be unable to fertilize oocytes in vitro on their own [14].

The new *Zfy* DKO phenotypic data are in par with the in-silico findings showing deregulation of spermatogenesis specific genes and pathways with this genotype. This is not true for *Zfy2* KO, for which subfertility and some spermiogenesis defects are not linked to spermatogenesis-related DEGs and pathways. Nevertheless, the data confirm and extend previous conclusion that loss of *Zfy2* and *Zfy* DKO affects fertility and prompts future investigations aiming to link identified DEGs with this phenotype.

### Loss of *Zfy* leads to upregulation of apoptotic genes in SC1 and SC2 cells and increased germ cell apoptosis

**Fig. 6:**
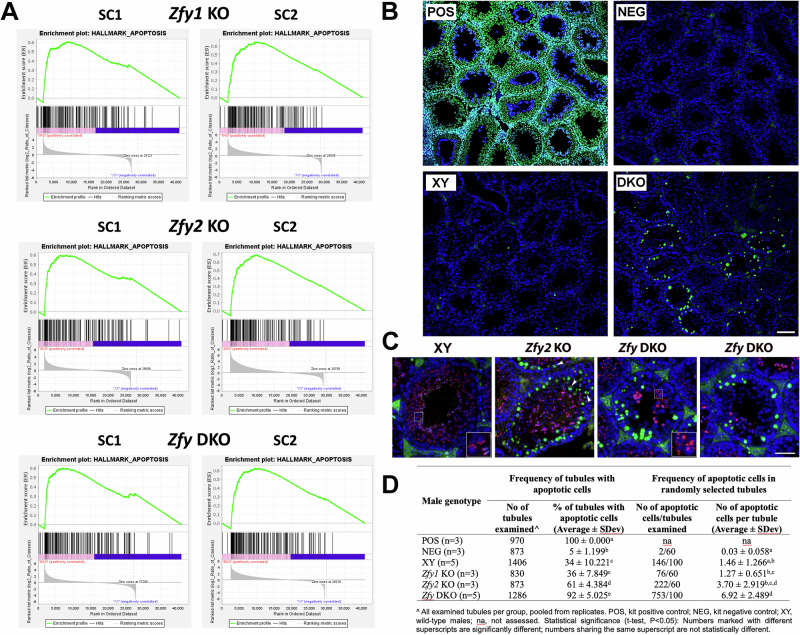
Apoptosis gene sets are enriched in *Zfy* KO spermatocytes. **A** Gene set enrichment analysis (GSEA) results showing significant enrichment (FDR < 0.05%) of the hallmark apoptosis gene set in *Zfy* KO SC1 and SC2 cells. For each pathway, enrichment plots depict graphical view of the enrichment score of each gene. **B**–**D** TUNEL staining of *Zfy* KO males testis cross sections. Testis sections from *Zfy1* KO, *Zfy2* KO, *Zfy* DKO (DKO) and wild-type male serving as genotype control (XY) were examined for presence of apoptosis. Two ‘assay controls’ were included: sections from XY treated with DNA-se serving as a positive control (POS) and sections from XY stained in the absence of TdT enzyme serving as a negative control (NEG). **B** Exemplary images showing frequency of apoptotic cells in DKO and XY, with POS and NEG serving as assay controls. Cell nuclei are stained with DAPI (blue) and apoptotic cells stain green. Scale, 100 µm. **C** Exemplary higher magnification images of selected tubules from XY, *Zfy2* KO and *Zfy* DKO males. Cell nuclei are stained with DAPI (blue), acrosomes are stained with lectin (red), and apoptotic cells stain green. Lectin staining enables observations of the acrosome development. As acrosome develops, it is first seen as a round acrosomal vesicle (visible as a lectin-stained single large dot), then flattens and spreads over the round spermatid surface (seen as variable size semi-circle, see XY, inset) and then elongates along the dorsal side of elongating spermatid nucleus (see XY, inset). In DKO males, lectin staining pattern is abnormal, with many small, punctuated dots, indicative of the problems with acrosome development. A combined DAPI and lectin staining helps defining the stages of spermatogenesis and identification of germ cells in each tubule. Insets, 3x magnification. Scale, 50 µm. **D** Quantitative analysis of TUNEL assay data. To assess the frequency of tubules with apoptotic cells, all tubules in a single testis cross-section per male were examined. The average number of tubules per cross-section was 283 ± 35, range 229–334. To assess the frequency of apoptotic cells within tubules, 20 tubules were randomly selected and apoptotic cells within counted.

In contrast to RS, many enriched pathways and gene sets identified in SC1 and SC2 were related to general cellular function and not reproduction-specific pathways (Tables S4, 5; File S1). GSEA found only a handful of enriched gene sets in *Zfy1* KO SC1 and SC2 (Table S4). To conduct a more comprehensive investigation of de-regulated pathways in *Zfy* KO germ cells, we performed GSEA using all databases (mouse hallmark database, mh.all). During meiosis, *Zfy* have been shown before to regulate apoptotic checkpoints which eliminate chromosomally aberrant spermatocytes [9, 10]. Enrichment of genes related to apoptosis (HALLMARK-APOPTOSIS) was observed with *Zfy1* KO, *Zfy2* KO, and *Zfy* DKO SC1 and SC2 (Table S8). GSEA enrichment plots (Fig. 6A) demonstrated that a disproportionate number of genes related to apoptosis were upregulated in *Zfy* KO SC1 and SC2. Several pathways implicated in modulating apoptosis were also enriched in *Zfy* KO spermatocytes: HALLMARK-P53-PATHWAY, HALLMARK-TGF-BETA-SIGNALING, and HALLMARK-KRAS-SIGNALING-UP (Table S8). Upregulation of individual genes from apoptosis-related pathways was also detected with RT-qPCR in purified primary and secondary spermatocytes from *Zfy* DKO males (Fig. S5D). The most strongly upregulated gene was an apoptosis regulator gene *Bcl2l*; immunofluorescence analysis of testis sections demonstrated that BCL2L protein level was higher in testes from *Zfy* DKO as compared to XY (Fig. S11).

Because *Zfy* regulates apoptotic checkpoints, and because upregulation of apoptosis-related genes was observed in *Zfy* KO spermatocytes, we performed terminal deoxynucleotidyl transferase dUTP nick end labeling (TUNEL) staining to test for DNA damage and apoptosis in testis sections. *Zfy* DKO males had significantly more apoptotic germ cells in seminiferous tubules than XY controls (Fig. 6B). The results of TUNEL assay were quantified to assess the frequency of seminiferous tubules containing apoptotic cells and the frequency of apoptotic cells per tubule. Only few apoptotic cells could be found in some of the tubules from XY males and their staining intensity was low. *Zfy1* KO males resembled XY. *Zfy2* KO and *Zfy* DKO males had more than 60% and 90% of seminiferous tubules containing apoptotic germ cells: leptotene and zygotene spermatocytes and meiosis I and II cells, based on their locations in the seminiferous epithelium and tubule stage. These two genotypes also had more apoptotic cells per tubule, and staining intensity was stronger (Fig. 6C, D).

Together, the data show that loss of *Zfy* leads to testicular germ cell death and confirm critical role of *Zfy* in regulation of apoptosis during spermatogenesis.

### Loss of *Zfy* deregulates the splicing of hundreds of genes

**Fig. 7:**
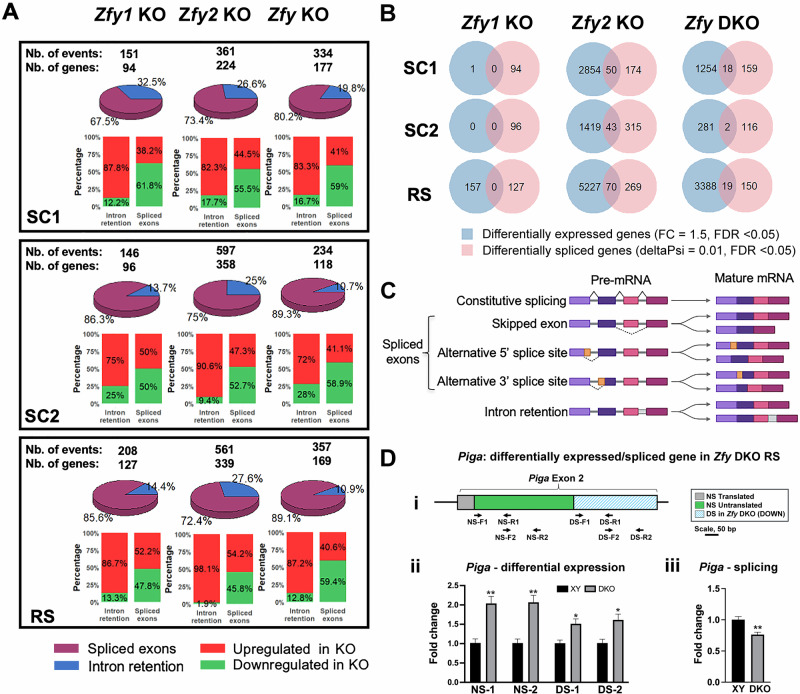
*Zfy* loss deregulates the splicing of hundreds of transcripts. Comparisons are shown for *Zfy1* KO, *Zfy2* KO, and *Zfy* DKO against XY, across three germ cell types: primary spermatocytes (SC1), secondary spermatocytes (SC2), and round spermatids (RS). **A** Pie chart and bar graph representing the distribution of differential splicing events, with a threshold of deltaPSI = 10% and FDR < 0.05, identified by overlapping results from both rMATS and Whippet approaches. **B** Venn diagrams illustrating the overlap between differentially expressed genes (DEGs, with FC = 1.5, FDR < 0.05) and splicing-regulated genes (deltaPSI = 0.01, FDR < 0.05). **C** Types of splicing events. **D** Validation of splicing. **i** diagram of PCR strategy to detect differential splicing of *Piga* in round spermatid (RS) from *Zfy* DKO (DKO) males. Non-spliced (NS) coding regions are shown in green, NS non-coding regions in gray, and downregulated DS regions in crossed blue. Primer sequences are shown by arrows, with two primer pairs for the NS region and two primer pairs for the DS region. Each primer pair was used for quantitative RT-PCR, normalized to three reference genes (*Ppia*, *Rsp18*, and *Rplp0*). **ii** Results from qPCR for the four primer pairs shown in (**i**). The data are average ±SEM, with *n* = 4 males per genotype. A higher expression was seen with *Zfy* DKO than with XY for all primer pairs, consistent with differential expression analysis and qPCR validation of *Piga* overexpression in *Zfy* DKO RS (see Fig. S5E). However, the DS region showed lower levels than the NS region in DKO, suggesting that the alternatively spliced region is downregulated. **iii** The downregulation of the DS region was confirmed by dividing of the average of DS regions by the average of NS regions for each male. The data shown are averages ±SEM, with *n* = 4 males per genotype. Statistical significance (*t*-test): *P < 0.05; **P < 0.01.

Alternative splicing has been shown to be particularly dynamic during spermatogenesis [38]. Making use of our large RNA-seq datasets, we investigated this process in *Zfy* KO models. Using a dedicated splicing-detection tool rMATS (with a differential percent spliced-in >10%), we found several thousand of alternative splicing events concerning at least 2700 genes in all *Zfy* KO models, whatever the cell stage (Fig. S12A). Interestingly, *Zfy1* itself was found differentially spliced in *Zfy2* KO RS versus XY RS. Specifically, *Zfy1* exon 4 was found more retained, while a small region in the 5’ UTR (corresponding to exon 2b) was found more frequently skipped in *Zfy2* KO RS (Fig. S12B, C). In parallel, we used a more stringent approach, which consists of integrating rMATS output with that of a different splicing-detection tool Whippet (with a differential percent spliced-in >10%). This analysis showed several hundreds of genes with at least one alternative splicing event that were deregulated in *Zfy* KO SC1, SC2 and RS compared to XY samples (Fig. 7A–C). For both analyses, most events were alternatively spliced exons, which were upregulated in *Zfy* KO versus XY, while alternatively retained introns represented between 10 to 30% of events (Fig. 7A and Fig. S12A). When we compared the differentially spliced genes with DEGs, very few were in common (Fig. 7B and Fig S12D), however, similar pathways were found enriched as shown with GO analyses (Fig. S13) indicating that splicing deregulation could also contribute to *Zfy* KO phenotypes. Deregulation of individual genes that were detected as deregulated using RNA-seq and that were also among genes detected as differentially spliced was confirmed with qPCR in purified round spermatids from *Zfy* DKO males (Fig. S5E). The actual splicing event of an exemplary gene from this group was confirmed with the same method (Fig. 7D).

### ZFY regulates apoptosis genes in human cell culture but does not specifically target sex-linked genes

**Fig. 8:**
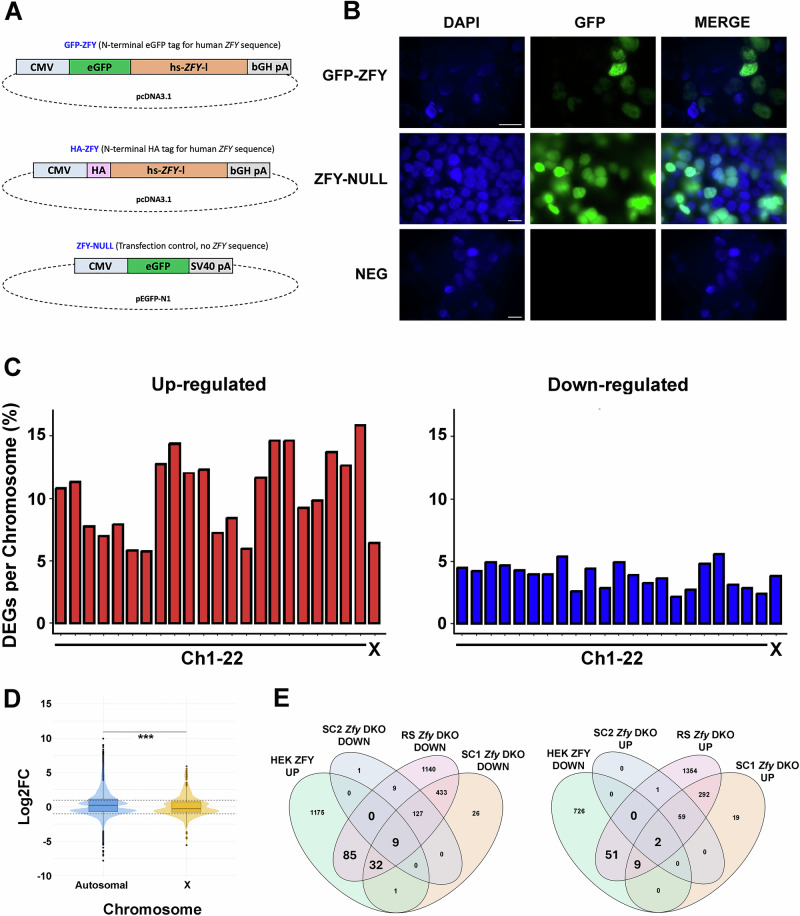
Transcriptome analysis of ZFY-transgenic HEK cells. **A** Strategies for establishing ZFY transgenic HEK cell lines. The two ZFY expression constructs were cloned into the pcDNA3.1 sequence (Addgene Plasmid #129020) and contained the open reading frame for the long isoform of human ZFY (hs-*ZFY*-l) accompanied by an N-terminal eGFP sequence (GFP-ZFY) or an N-terminal HA tag (HA-ZFY). As a negative transfection control, a construct containing a GFP reporter and no ZFY sequence (ZFY-NULL) was used. CMV, human cytomegalovirus enhancer and promoter; bGH pA, bovine growth hormone polyadenylation signal; SV40 pA, simian virus 40 polyadenylation signal. **B** Reporter (eGFP) expression in HEK cells transfected with GFP-ZFY and ZFY-NULL expression constructs, with non-transfected HEK cells (NEG) shown as a reference. Scale, 15 µm. **C** Up-regulated (red) and down-regulated (blue) DEGs (FC > 1.5, FDR < 0.05) listed by chromosome for GFP-ZFY transfected HEK cells. Percentage of DEGs per total gene number on each chromosome (human autosomal chromosomes, Chr1-22, and sex chromosomes, X). **D** Boxplot distributions showing the mean Log_2_ FC of autosomal genes compared to X genes. Statistical analysis was performed using the t-test with adjusted *P* value, *** = 0.001. **E** Venn diagrams showing the concordantly regulated genes in the different experimental systems. HEK ZFY HEK cells transfected with hs-*ZFY*-l, SC1 primary spermatocytes, SC2 secondary spermatocytes, RS round spermatids, DOWN downregulated, UP upregulated.

After completing the bioinformatics analysis exploring the indirect regulatory targets of endogenous *Zfy* using the *Zfy* KO models, we next performed transient overexpression assays with *ZFY* constructs and human embryonic kidney (HEK293) cells. As spermatocytes and spermatids do not proliferate in culture, and do not readily take up and express transfected constructs, the experiments with HEK293 cells allowed us to identify the direct regulatory targets of ZFY. Additionally, they allowed us to see if the ZFY mechanisms of regulation were specific to the murine male germline or if these functions were conserved across different cell types and species. Our goal was also to look at the immediate/early consequences of *ZFY* activity in vitro, expanding over the analyses of functional consequences of *Zfy* loss in vivo, in the testis. Transient overexpression assays are typically used to explore the direct, as opposed to indirect regulatory targets of transcription factors. We prepared N-terminally tagged constructs expressing full-length human ZFY in a pCDNA3.1 backbone, under the control of the CMV promoter, and transfected these into HEK293 cells. Equivalent results were obtained with both N-terminal HA tag and N-terminal GFP tags: for brevity we focus on the latter here. For this comparison, overexpression of free EGFP from pEGFP-N1 was used as a control for the effects of the transfection process (Fig.8A, B). Forty-eight hours post-transfection, a pairwise comparison of DEGs between pCDNA3.1/ZFY-GFP and pEGFP-N1 showed that 10.22% of all genes were significantly up-regulated by at least two-fold by ZFY overexpression, and 3.97% of all genes were significantly down-regulated by at least two-fold. The preponderance of overexpressed genes agrees with a net activatory role for ZFY, either mediated through direct promoter activity or some broader effect on opening of chromatin.

HEK293 is a female cell line (i.e., ZFY null) and thus the comparison of transfected to non-transfected cells is most closely analogous to the DKO mouse data, in which there was a striking upregulation of sex-linked genes in both SC1 and RS cells. This was not observed in the HEK293 data set. While the magnitude of change for the most highly regulated genes did differ between autosomes and X chromosome, this applied to both up- and down-regulation, with autosomal up-regulated genes in general showing slightly higher fold changes (Fig. 8C, D). To search for commonalities between the data sets, after matching orthologous genes, we intersected the DEG lists for each mouse germ cell type with the HEK293 cell data, searching for genes that were concordantly regulated. Those that were upregulated in the transfection experiment and downregulated in the knockout testes were termed Consistently Activated Genes (CAGs) and those with the opposite pattern were termed Consistently Repressed Genes (CRGs) (Fig. 8E). There was very high agreement between germ cell subtypes, so for downstream pathway analysis using Reactome, we focused on genes showing concordant regulation between transfected HEK293 cells and DKO round spermatids (larger font in Fig. 8E). CAGs (*n* = 126) were significantly enriched for a set of pathways involved in cell-cell signaling, including IZUMO4 with a role in sperm-egg binding, and various other genes annotated as synaptic proteins. CRGs (*n* = 62) showed significant enrichment for genes involved in the G1/S transition, DNA damage response and/or replicative stress (File S2).

## Discussion

In the present article, we investigated the molecular consequences of *Zfy* loss in male germ cells by performing large-scale comparative transcriptomic analyses. We found that loss of both *Zfy1* and *Zfy2*, or loss of *Zfy2* alone, significantly alters the male germ cell transcriptome, whereas loss of *Zfy1* leads to only minor transcriptional changes. The strongest expression changes were observed in round spermatids, although de-regulated pathways were also identified in spermatocytes. In vitro analysis of cells expressing human *ZFY* confirmed that regulation of pathways related to apoptosis and cell cycle is conserved across species and cell types, whereas the regulation of sex-linked genes and spermiogenesis is confined to the male germline. Together, our data provide novel insights into how ZFY regulates various processes throughout meiosis and spermiogenesis.

### *Zfy* loss alters pathways relating to DNA packaging and chromatin organization

We demonstrated that ZFY regulates genes that are critical for proper chromatin packaging in post-meiotic cells. PEA showed that genes encoding for histones were upregulated in round spermatids from *Zfy* DKO and *Zfy2* KO, and GSEA found that nucleosome and chromatin gene sets were up-regulated in these cells from *Zfy* DKO. The X-chromosome carries several genes encoding H2A variants, such as H2AL and H2A.B, which are up-regulated in in *Zfy* DKO RS. All are relevant candidates to explain the defects in chromatin packaging of *Zfy* DKO sperm. The autosome-encoded H2AL1 has previously been shown to be essential for histone to protamine transition [39] and loss of H2A.B variants has been reported to alter spermatid chromatin structure [40]. Genes encoding H2A canonical histones are also up- or down-regulated, such as *Hist1h2ai* and *Hist2h2be*.

The in-silico findings were confirmed by several approaches demonstrating that histone-to-protamine transition is abnormal in *Zfy* DKO and that *Zfy* DKO sperm have poorly packaged chromatin, incomplete protamination, and increased histones retention. *Zfy2* KO appear to have a milder genotype, while *Zfy1* KO did not differ from the XY. Thus, *Zfy* may act as a transcriptional regulator of gene sets related to postmeiotic chromatin remodeling and compaction. Y chromosome deficiencies are often linked with problems with postmeiotic chromatin remodeling. Deficiency or loss of genes from the long arm of mouse Y chromosome has been shown to result in poor sperm chromatin organization [41–43]. The deficiencies or absence of genes from the Y chromosome short arm, where *Zfy* genes are located, cause predominantly premeiotic or meiotic arrests [44, 45]. However, when postmeiotic cells are present and spermiogenesis takes place, the resulting elongated spermatids and sperm are invariably abnormal, often with poorly condensed chromatin [13]. Our prior transmission electron microscopy characterization of chromatin condensation in sperm from *Zfy2* DKO males revealed its impairment [14]. Also, the transcriptome data acquired after knockout of the *ZFX* gene family in human cultured cells revealed upregulation of histone-related gene sets [2].

### *Zfy* loss alters spermatogenesis pathways

We found that pathways related to sperm flagellum and axoneme assembly were downregulated in spermatids from *Zfy* DKO males. This downregulation was observed only with the PEA using DEGs with a fold change greater than 1, and not with a higher fold-changed threshold. Thus, *Zfy* may modulate these processes by inducing small changes in transcript expression. The deregulation of sperm tail formation pathways matches with very severe morphology defects of sperm from *Zfy2* KO and *Zfy* DKO [14] and motility defects of sperm from *Zfy* DKO reported here. Sperm motility defects were also observed with *Zfy* KO mice produced by two other investigative groups [46, 47]. Nakasuji et al. [46] reported abnormal sperm motility as well as defects of axoneme and outer dense fibers, two structural elements of the sperm tail, with *Zfy2* KO and *Zfy* DKO males while Subrini et al. [47] have shown, using a threshold-free GSEA, that motility pathways were downregulated in whole testis. Thus, results from three independently obtained *Zfy* DKO mouse lines point to changes in both phenotype and transcriptome related to spermatogenesis.

The expressional changes in pathways related to sperm flagellum and axoneme assembly were not observed in spermatids from *Zfy2* KO males, and sperm these males did not display motility defects. However, sperm from *Zfy2* KO males had limited-to-no ability to fertilize oocytes in vitro. We suspect that this deficiency is due to sperm defects affecting their ability to participate in one or more steps required for successful fertilization other than motility, for example capacitation, acrosome reaction, or zona pellucida binding and penetration. Future tests are required to test for these possibilities.

Using mutant mice with Y chromosome deficiencies and transgenic rescue approach we have shown before that the presence of *Zfy2* is critical for sperm formation. Mice lacking Y chromosome but transgenic for two other Y-derived genes, *Sry* driving sex determination and *Eif2s3y* initiating spermatogenesis, produced only round spermatids [45]. Addition of the *Zfy2* transgene allowed for spermatid elongation [13, 15] and ultimately production of sperm functional in intracytoplasmic sperm injection (ICSI) [15]. The phenotype of *Zfy2* KO males [14] (and this study), and prior studies with mice with Y chromosome deletions [9, 11–13, 15] support that *Zfy2* is involved in regulation of spermiogenesis pathways. Greater sequencing depth may be required to detect the deregulation of spermatogenesis pathways in *Zfy2* KO round spermatids.

MEA suggests that ZFY has coregulators with known functions in spermatogenesis. ZFP105, a transcription factor candidate for enriched motifs identified in *Zfy2* KO and DKO RS, is suspected to have roles in spermatogenesis, with *Zfp105*-mutant mice exhibiting reduced fertility [48]. Like *Zfy2*, *Zfp105* is strongly expressed in round spermatid, making it an attractive candidate to co-regulate spermatogenesis with *Zfy*. MEIS1, another transcription factor candidate for motifs enriched in *Zfy* DKO RS, has also been implicated in regulating spermatogenesis from somatic cells, as Sertoli-cell specific knock-down of *Meis1* leads to male infertility [49]. However, the function of MEIS1 in male germline is unconfirmed, and *Meis1* is only expressed at low levels in spermatocytes and spermatids. Conditional knock-out of *Sox17*, another transcription factor candidate for motifs enriched in *Zfy2* KO DEGs, results in irregular detachment of immature spermatids [50].

### The three *Zfy* KO models vary in how the loss of *Zfy* affects their transcriptome

We observed very few transcriptional changes in germ cells form *Zfy1* KO males as compared to germ cells from *Zfy2* KO and *Zfy* DKO. This agrees with good fertility and normal spermatogenesis reported with *Zfy1* KO males [14] and previous studies performed with mutants with Y chromosome deficiencies supporting indirectly that *Zfy2* is a stronger player in spermatogenesis than *Zfy1* [11–13]. Based on the phenotypic differences between *Zfy2* KO and *Zfy* DKO males, with the latter being fully infertile with severe spermatogenesis defects and the former being less affected [14], we expected to find stronger transcriptome changes in germ cells from *Zfy* DKO males. Surprisingly, more DEGs were observed with *Zfy2* KO, in all germ cell types, indicating that the loss of *Zfy2* alone was clearly more detrimental to transcriptome than the loss of both *Zfy1* and *Zfy2*. We currently favor the hypothesis that this unexpected outcome is due to *Zfy2* loss influencing *Zfy1*. Loss of both *Zfy1* and *Zfy2* (in *Zfy* DKO) removes the regulatory function of both homologs. Loss of *Zfy2* alone removes *Zfy2* regulatory function, but also strongly affects *Zfy1*-dependent gene regulation, and the consequences of this latter effect are greater than the loss of *Zfy1*. Our findings stand in contrast to results from a recent transcriptome analysis of whole testis from *Zfy* KO mice showing that loss of both *Zfy1* and *Zfy2* have more severe effects than loss of *Zfy2* alone [47]. The differences between us and this study could be explained when one considers that purified spermatocytes and spermatids, which we used, are more representative of the male germline than all testicular cells combined.

Our data suggest that *Zfy* function as a transcriptional regulator is more intricate than previously anticipated. In mice, there are two main *Zfy* transcript variants created by splicing: a long variant that predominates for *Zfy2* and a short variant that predominates for *Zfy1* [3]. Although both variants contain an intact zinc finger DNA-binding motif, in vitro testing has shown that only the long variant has an active transactivating domain; thus the short variant can bind but cannot transactivate [3]. Under physiological conditions, the active and inactive variants exist in equilibrium, presumably competing for binding to promoter regions of the regulated genes and interacting with different proteins. When the predominating active *Zfy2* transcripts are eliminated, the inactive *Zfy1* transcripts could cause a cascade of transcriptional deregulation, perhaps blocking other transcription factors from accessing DNA, ultimately resulting in more significant expressional changes than those observed with loss of both homologs. This hypothesis could be confirmed in future with assays measuring the genomic and protein binding patterns of ZFY in vivo.

Our data add another layer of complexity since we see that *Zfy* loss deregulates alternative splicing of at least several hundreds of genes. Only a few are in common with DEGs, yet pathway analyses indicate that they are involved in similar pathways (such as motile cilium and nucleosome/histone pathways) and could therefore contribute to the observed phenotypes. It is worth mentioning that the splicing of *Zfy1* itself appears to be affected by *Zfy2* loss, and to favor the long isoform to the detriment of the short *Zfy1* transcript isoform, in round spermatids. This shift in *Zfy1* isoforms could have more detrimental effects on the number of DEGs but overall compensate partially for *Zfy2* (long isoform) loss. Other compensatory effects or at least “crosstalk” with genes related to *Zfy* could also be at stake. We indeed found that the X-linked *Zfx* is downregulated in primary and secondary spermatocytes from *Zfy2* KO, while the autosome encoded *Zfp711* is significantly upregulated in round spermatids from *Zfy* DKO. To fully understand this phenomenon would require the production of multiple new mouse mutants and remains a challenge for future studies.

### *Zfy* loss bears a strong effect on apoptosis-related pathways

The most significant transcriptional changes observed in *Zfy* KO spermatocytes included the upregulation of apoptosis gene sets. GSEA found that in addition to the apoptosis genes set, several other gene sets directly related to apoptosis were upregulated: P53, KRAS, and TGFβ signaling pathways. The P53 pathway becomes activated upon DNA damage and serves as a key regulator of DNA repair and programmed cell death [51]. Transforming growth factor β (TGFβ) induces apoptosis via activation of the pro-apoptotic Bim protein [52] (reviewed [53]). The KRAS signaling pathway is also known to play an important role in programmed cell death [54]. Using TUNEL assay to measure apoptosis in testis sections from *Zfy* KO males we observed an increased presence of apoptotic spermatocytes in *Zfy2* DKO, and to a lesser extend in *Zfy2* KO, confirming that loss of both *Zfy* homologs or *Zfy2* alone alters germ cell homeostasis. Thus, *Zfy* could control the checkpoints of meiotic apoptosis by directly or indirectly interacting with these pathways. This matches with previous data acquired with mutant mice and *Zfy* transgene rescue showing that *Zfy* are involved in control of spermatogenic quality functions during the pachytene stage of meiosis and during meiosis I by triggering the apoptotic elimination of spermatocytes [10, 11] and regulating meiotic sex chromosome inactivation (MSCI) [9] (reviewed in [1]).

Although only viable cells were included in sorted germ cell populations used for RNA isolation and sequencing, transcription of apoptosis related genes precedes any visible manifestation of apoptosis, and TUNEL assay data confirmed presence of high number of apoptotic spermatocytes in *Zfy* DKO and *Zfy2* KO testes. Thus, we cannot exclude that the observed upregulation of apoptosis genes linked to *Zfy* loss is a consequence of cell death rather than its cause. The data are clear in showing that *Zfy* loss alters apoptosis regulation, but the details of this regulation require further investigations.

### *Zfy* loss leads to altered expression of sex chromosomes-encoded genes

Prior transgene rescue studies have shown that *Zfy* is critical for initiation and regulation of MSCI [9, 10]. In the present study, we observed upregulation of sex-linked genes in spermatocytes and spermatids from *Zfy* DKO males. Our analyses with primary spermatocytes indicate both an incomplete MSCI and a premature expression of postmeiotic genes while, in round spermatids, point to a less efficient postmeiotic sex chromatin repression (PSCR).

Upregulation of Y chromosome-linked genes was also detected with *Zfy1* KO and *Zfy2* KO males but one must be cautious with the interpretation of this result due to the repetitive nature of the mouse Y chromosome long arm. Indeed, it carries >100 copies of the genes *Sly*, *Ssty1* and *Ssty2* [55] and they appear to be the main contributors of Y gene upregulation, at least in spermatocytes.

Surprisingly, X-linked genes were found downregulated in spermatocytes from *Zfy1* KO and *Zfy2* males. The reason for the X-linked downregulation in *Zfy1* and *Zfy2* KO spermatocytes is less clear. Given that *Zfy* has other regulatory functions during meiosis 1, including a recently discovered role in X-Y pairing [47], the transcriptional changes of sex-linked genes may not be due solely to *Zfy* function in MSCI, but rather be a result of deregulation of other pathways controlled by *Zfy*.

### Global transcriptome regulation by ZFY is observed in vitro and is not restricted to mouse

Overall, the comparison of germ cell data to transfected cells is instructive in several areas. First, the specific sex chromosome deregulation in response to germline knockout was not observed in the somatic cell transfection experiment. We therefore conclude that ZFY is unlikely to directly target large numbers of sex chromosome genes, and that the germline changes are a downstream effects of broader chromatin changes, potentially related to different chromatin environment as a consequence of MSCI/PMSC in the *Zfy* DKO model, with more complex factors at play in the single knockout models. Second, while in the germline ZFY appears to regulate expression of many cell-specific genes associated with spermatid functions, we do not see widespread ectopic activation of these pathways in the transient transfection experiment. Third, regulation of DNA damage repair and cell cycle genes emerges as one common factor conserved across species and cell types. Finally, under transient transfection conditions we see predominantly gene up-regulation, as expected if ZFY is a transcriptional activator. The more balanced up/down-regulation seen in the knockout mouse models is likely to represent an equilibrium state encompassing both direct and indirect effects of ZFY.

## Conclusions

Collectively, the emerging picture is that ZFY expression regulates a significant fraction of the total transcriptome, both in the knockout testes (i.e., chronic depletion model) and in HEK cell transfection (transient overexpression). This broad effect, coupled with the finding that chromatin pathways and cell cycle pathways are perturbed, argues that ZFY may exert its functions via a general modulation of chromatin regulation, rather than through direct action on a small number of direct targets. This is consistent with the very general binding motif (GGCCT) identified from previous in vitro and in silico work [2, 56] and the presence of ZFY broadly across CpG island promoters in prostate cells [2]. Given widespread interactions with broad classes of promoter, cell-type specific effects of ZFY are likely to be sculpted by interactions with other lineage-specific transcription factors. Generalized effects on chromatin regulation and DNA damage responses may explain the sex chromosome deregulation in germ cell types, given the known close connections between DNA damage response (DDR), MSCI and subsequent PMSC.

## Methods

### Biological resources

The mice were used for breeding, as sperm and oocyte donors for assisted reproduction, as vasectomized males and surrogate/foster females for embryo transfer, and for male germ cell collections. Two wild-type strains, bred in-house, were used, CD-1 (originate from Charles River Laboratories Crl:CD-1(ICR); strain code: 022) and C57BL/6J (originate from Jackson Laboratory, strain code: 000664).

The mice were fed ad libitum with a standard diet and maintained in a temperature- and light-controlled room (22 °C, 14 h light/10 h dark), in accordance with the guidelines of the Laboratory Animal Services at the University of Hawaii and guidelines presented in National Research Council’s “Guide for Care and Use of Laboratory Animals” published by Institute for Laboratory Animal Research of the National Academy of Science, Bethesda, MD, 2011. The protocol for animal handling and treatment procedures was reviewed and approved by the Animal Care and Use Committee at the University of Hawaii (animal protocol number 06-010).

*Zfy1* KO, *Zfy2* KO and *Zfy2* DKO males used in this study were produced by us before using CRISPR/Cas9 [14]. The *Zfy1* and *Zfy2* KO colonies were propagated by standard breeding, and the *Zfy* DKO mice were reproduced by intracytoplasmic sperm injection (ICSI) or round spermatid injection (ROSI). All *Zfy* KO males used in this study were on C57BL/6 genetic background. *Zfy1* KO were originally produced as pure C57BL/6 while *Zfy2* KO and *Zfy2* DKO males were produced on mixed background and subsequently backcrossed to C57BL/6 for at least 10 generations using breeding and assisted reproduction, respectively.

### Reagents

All chemicals were obtained from Sigma Chemical Co. (St Louis, MO, USA) unless otherwise stated.

### In vitro fertilization

#### Oocyte collection

Female mice were induced to superovulate with the injection of 5 IU pregnant mares’ serum gonadotrophin (eCG, ProSpec, East Brunswick, NJ, USA) and 5 IU human chorionic gonadotrophin (hCG, ProSpec, East Brunswick, NJ, USA) given 48 h apart. Oocyte collection and subsequent oocyte manipulation, including in vitro fertilization and microinjections, were done in HEPES-CZB [57], with subsequent culture in CZB medium [58] in an atmosphere of 5% CO_2_ in air.

#### In vitro fertilization (IVF)

IVF was done as described by us [59]. For IVF, the epididymal sperm were expressed from dissected cauda epididymides directly into a drop of HTF medium [60] (capacitation drop) and sperm concentration was quantified. Sperm were capacitated in HTF for 1.5 h at 37 °C in a humidified atmosphere of 5% CO_2_. Then, a portion of capacitated sperm suspension was transferred to another drop of HTF medium (fertilization drop) to achieve a concentration of 5 × 106/mL. Oocyte-cumulus cell complexes were released to the fertilization drop and gametes were co-incubated for 4 h. After co-incubation, the oocytes were washed several times with HEPES-CZB, followed by at least one wash with CZB. Embryos were cultured in CZB until pronuclei stage followed by cryopreservation or allowed to develop further.

### Germ cell isolation

Primary spermatocytes (SC1), secondary spermatocytes (SC2), and round spermatid (RS) cells were isolated from wild type (XY), *Zfy1* KO, *Zfy2* KO, and *Zfy* DKO mice via germ cell isolation and fluorescence-activated cell sorting (FACS). In each sorting experiment two testes were used. The testes were dissected, and tunica was removed. The seminiferous tubules were incubated in 167.5 U/mL collagenase IV (Worthington Biochemical, Lakewood, NJ, USA) for 10 min at 37 °C, washed twice with Krebbs Buffer, and then incubated in 0.25% Trypsin-EDTA with 20 µg/mL DNAse I for 25 min at 37 °C, with vigorous pipetting every five min to dissociate cells. The cells were then run through a 30 µm strainer, centrifuged for 6 min at 500 × *g*, and resuspended in 180 µL PBS-S (1% FBS, 10 mM HEPES, 1 mM pyruvate, and 1 mg/mL glucose in PBS). Spermatogonial stem cells (SSCs) were removed by addition of 20 µL CD90.2 MicroBeads (Miltenyi Biotec, Bergisch Gladbach, Germany), incubation on ice for 30 min, suspension in 3 mL PBS-S and separation through magnetic column.

Prior to FACS, the germ cells were resuspended in FACS buffer (1 mM EDTA, 25 mM HEPES, 1% FBS in PBS) at a concentration of 2 million cells per mL. This suspension was stained with 10 µM Vybrant™ DyeCycle™ Violet Stain (ThermoFischer, Waltham, MA, USA) for 30 min at 37 °C. Cells were sorted with BD FACS Aria III (BD Biosciences, Franklin Lakes, NJ, USA). Each FACs sorting included a “test sort” performed on small number of mixed germ cells. The sorted fractions of roughly 5000 cells were checked for purity by morphological assessment. If purity in “test sort” was >90%, the remaining mixed germ cells were sorted under the same conditions. The purity was checked again and when >90% samples divided into fractions containing 200,000–300,000 cells. The cells were spun down for 5 min at 500 × *g*, snap frozen in LN_2_, and stored at −80 °C for RNA extraction. The three germ cell types in a single biological replicate were from a single male.

In the initial stages of the project, we verified the purity of fractions using immunostaining for H2AX (expressed only in SC1) and SYCP3 (expressed in SC1, and, to a lesser extent, SC2). Roughly 50,000 SC1, SC2, and RS cells were collected and surface spreads of spermatogenic cells were prepared as described earlier [11]. The following primary antibodies were used: anti-SYCP3 (1:250; Ab97672, Abcam), and anti-γH2AX (1:500; 9718, Cell Signaling). As secondary antibodies we used Goat anti-Mouse Alexa Fluor 594 (1:500; A21125, Invitrogen) and Goat anti-Rabbit Alexa Fluor 488 (1:500; A11008, Invitrogen). The cells were distinguished based on nuclear size, DAPI morphology, and the SYCP3 and γH2AX staining pattern. The average purity was ~95% for SC1, ~90% for SC2, and >99% for RS.

### RNA sequencing

RNA was extracted from each SC1, SC2, and RS cell fraction using the RNeasy Micro Kit (Qiagen, Hilden, Germany). RNA quality assessment, library preparation, and total transcriptome RNA sequencing were performed at the University of Hawaii Cancer Center Genomics and Bioinformatics Shared Resource (UHCC GBSR). RNA integrity of all samples was assessed using Bioanalyzer (Agilent, Santa Clara, CA). The cDNA libraries were prepared using the NEBNext® Ultra^TM^ II Directional RNA Library Prep Kit for Illumina (New England Biolabs, Ipswich, MA) before quality assessment with the Agilent Bioanalyzer. The Illumina NextSeq 2000 P3 flow cell (Illumina, San Diego, CA) was then used to perform paired-end sequencing of 150-bp reads (roughly 33 million reads for each sample).

Initially, a pilot experiment was performed with SC1 (*n* = 3) and RS (*n* = 3) isolated from XY and *Zfy* DKO males. Subsequently, an expanded experiment was done using all cell types (SC1, SC2, and RS, *n* = 3 each) and all genotypes (XY, *Zfy1* KO, *Zfy2* KO, and *Zfy* DKO). The SC1 and RS data for XY and *Zfy* DKO acquired with pilot and expanded experiments were compared and found similar (Fig. S1) and therefore the analyses described below were performed on combined data.

RNA-seq data analysis was performed as previously described [61]. FASTQ files were trimmed and filtering using BBduk (v38.23) and were aligned with STAR (v2.7.2 d) on the mouse genome (GRCm38.p6). STAR and samtools package (v1.3.1) were used to estimate raw read counts. Differential expression analysis was performed using DEseq2 and edgeR R packages on expressed genes (CPM > 1 in minimum 2 samples). DEGs were considered significantly deregulated when they passed FDR < 0.05. We performed two separate functional analyses, one using DEGs with at least a 1.5-fold expression change, as used in a previous *ZFY* transcriptome analysis on human cultured cells [2] and a second using genes with at least a 1.0-fold expression change.

To calculate the number of DEGs per chromosome, the number of DEGs on each chromosome was divided by the total number of transcripts reported in the Mouse Genome Informatics database (https://www.informatics.jax.org). We excluded ribosomal RNA and microRNA genes from these calculations, as our sequencing method would not detect these types of RNA.

To identify enriched pathways we used four methods: gene set enrichment analysis (GSEA, [25, 62], PEA with the Enrichr function in ClusterProfiler [24, 63], functional analysis with the EnrichR online tool [64, 65], and MEA with the Hypergeometric Optimization of Motif EnRichment (HOMER) software [27]. GSEA considers all genes in relation to their expression level, ClusterProfiler uses DEGs with non-regulated genes to account for bias, and the EnrichR tools only consider DEGs to identify enriched pathways. We searched across three GO databases: Biological Processes, Cellular Components (CC), and Molecular Function (MF). We also considered the Hallmark (HM) database for conditions for which we did not identify significantly enriched pathways with all four methods. HOMER analysis identifies enriched motifs, identifying potential transcription factor candidates for each motif, and has a function to perform GO analysis.

### Expression analyses

Real time qPCR on whole testis or purified germ cells was performed to confirm deregulation of genes. For validation we selected genes that were the most strongly deregulated in RNA-seq data and that were also the most highly expressed in cells of interest. RNA was extracted from testis or purified cells using the RNeasy Micro Kit (Qiagen, Hilden, Germany). Reverse transcription of polyadenylated RNA was performed with Superscript Reverse Transcriptase IV, according to the manufacturer’s guidelines (Invitrogen, Carlsbad, CA, USA). In analyses of purified cells, the cDNA, synthesized from 50 ng RNA, was diluted 2–8-fold depending on the ability to accurately detect target gene expression. All reactions were carried out in triplicate per assay and normalized three ubiquitously expressed genes (*Ppia*, *Rsp18*, and *Rplp0*). The Ct value for each individual sample was calculated by subtracting the Ct value of a tested gene from the geometric mean of the three loading controls. The Ct value was calculated by subtracting the Ct of each tested male from the average Ct of reference samples (XY males). The data were expressed as a fold value of expression level.

Stringent rules for data inclusion were applied. The gene was included in the analysis if: (1) the number of biological replicates was no less than 2 per genotype; (2) at least two technical replicates yielded data in any biological replicate; (3) at least 2 technical replicates were similar (a dissimilar technical replicate data were excluded from the average); (4) no amplification was observed in blanks. Primer sequences are shown in Table S9.

### Alternative splicing analysis

Splicing events were identified using rMATS [66] (version 4.12) and Whippet [67] (version 1.7), both with default parameters. For rMATS, RNA-Seq data were aligned to the reference genome using the STAR aligner and resulting BAM files were processed with default parameters to quantify differential splicing (DS) between wild-type and knockout conditions. In Whippet, raw fastq files were quantified individually, followed by DS analysis, also comparing wild types and knockouts with default settings. The Output files from both tools were processed using the Maser (version 1.20.0) and GeneStructureTools (version 1.23) R packages with parameters: *minCounts* = 5, *deltaPSI* = 0.001%, *FDR* = 0.05, and *probability* = 95. A splicing event was considered relevant if it was identified by both tools, with a minimum 1 bp overlap assessed using Bedtools [68] (version 2.30). Gene ontology (GO) enrichment analysis was conducted on the genes with significant splicing changes (deltaPSI=10%, FDR < 0.05). GO terms were identified using the EnrichGO() function in the clusterProfiler R package (version 4.8.3), with default settings. Enriched terms with an FDR < 0.05 were considered significant.

To validate splicing events the same RT-qPCR strategy described earlier was used using round spermatids from XY and *Zfy* DKO males. One gene, *Piga*, was selected because it was (1) the most strongly differentially spliced, (2) expressed highly in round spermatids, and (3) contained spliced exon regions large enough to design multiple primer sequences (at least ~150 base pairs long). Primers were designed over the differentially spliced (DS) and non-spliced (NS) region of coding exons. Five and four primer pairs were designed for DS and NS region, respectively, their efficiency was tested, and three pairs per region with the highest efficiency (~95–105%) were used. The fold change in expression for *Zfy* DKO (in relation to XY) was calculated for the DS and NS regions of coding exons. The occurrence of splicing was calculated by dividing the average expression of DS regions by the average expression of NS regions for each sample.

### Expression profiling in cultured cells

N-terminally tagged ZFY expression constructs were commercially synthesized by GenScript. The complete human open reading frame of *ZFY* was cloned into both a pcDNA3.1+N-eGFP and a pcDNA3.1+N-HA backbone using the Xhol/Xbal sites of the vector, placing the complete human *ZFY* gene in-frame with either a GFP- or HA-tag respectively as N-terminal fusions. The resulting constructs were transformed into NEB 5-alpha competent E. coli cells (NEB, #C2988J) and subsequently purified using a QIAprep Spin Miniprep Kit (Qiagen, Cat. No./ID: 27104).

To identify transcriptional responses to ZFY overexpression, human embryonic kidney HEK293 cells, a gift from Michelle Garrett (University of Kent) were cultured at 37 °C under humidified conditions, with 5% CO_2_. Cells were cultured in Dulbecco’s Modified Eagle Medium (DMEM, Gibco, 11574486) supplemented with 10% Fetal Bovine Serum (FBS, Gibco, 11570516) and 1% L-Glutamine–Penicillin–Streptomycin (Sigma-Aldrich, G6784). Cells were seeded into six-well plates and transfected with the purified tagged ZFY expression constructs during log phase growth. pEGFP-N1, containing GFP alone, was used as a negative control transfection. Transfection was performed using Lipofectamine 3000 (ThermoFisher Scientific, #L3000001), adjusting the manufacturer’s protocol for 2 µg of DNA per well in a 6-well dish. In test transfections, nuclear localization of the tagged constructs (and cytoplasmic localization of free EGFP from pEGFP-N1) was observed by fluorescence microscopy, and the integrity of the full-length tagged ZFY protein confirmed by Western blotting (data not shown). For the expression analysis, following a 48-h incubation period post-transfection, cells were harvested by trypsinization, and total RNA extracted using the Qiagen RNEasy mini kit (Qiagen, 74104).

Expression analysis was performed on triplicate biological replicate wells for each construct. Paired-end RNA sequencing of 150 bp reads was performed by Novogene, and the downstream bioinformatics analysis was performed in house [69]. Quality control of the raw sequencing reads was performed using FASTQC (v0.11.9) [70]. The FASTQ files were indexed and aligned to the reference human genome (release 105) using HISAT2 (v2.2.1) [71] and were subsequently sorted using Samtools (v1.14) [72]. Feature counts (v2.0.1) was used to count the fragments to generate a count matrix containing all the read data [73]. Differential expression analysis was performed on the count matrix in R studio (v3.2.2) using DESeq2 (v1.34.0) [74]. DEGs were considered significant when they had a p.adj < 0.05 and a > 1.5-fold change.

For comparisons of DE genes in testis vs HEK293 cells, gene orthologues were identified using UniProt and the gene lists were then filtered to select for gene putatively concordantly regulated by ZFY in both experimental systems (i.e., genes that were upregulated in DKO testes and downregulated in ZFY knock-in HEK cells, or vice versa). A pathway analysis using Reactome [75] was then carried out for these lists of concordantly regulated genes.

### Chromatin staining

Chromomycin-A3 (CMA3) staining was performed on sperm isolated from XY, *Zfy1* KO, *Zfy2* KO, and *Zfy* DKO males as previously described [76]. Epididymal sperm were released into HEPES-CZB medium and washed once with PBS. For a positive control, XY spermatozoa were incubated on ice for 15 min in 5 mM DTT, 0.5% Triton-X-100 in PBS to destabilize the sperm chromatin. The XY negative control and all other genotypes were incubated only in PBS for 15 min on ice. The sperm suspension was then layered onto 1 M sucrose solution in 25 mM Tris-HCl (pH = 7.4), centrifuged at 3000 × *g* for 10 min, incubated in 500 µL fixation solution (3:1 methanol:acetic acid) on ice for 5 min, and then spread on slides and air-dried for 20 min at 37 °C. Prior to CMA3 staining, the positive control samples were incubated in 2 M NaCl, 2 mM DTT in H_2_O for 15 min at room temperature before being washed with distilled water. Each slide was incubated in CMA3 staining solution (0.25 mg/mL CMA3 in 17.5 mM citric acid, 165 mM disodium hydrogen phosphate, pH = 7.0, supplemented with 10 mM MgCl_2_ and 1% DMSO) for 30 min at room temperature. The slides were mounted with Vectashield Mounting Medium for Fluorescence with DAPI (Vector Laboratories, Burlingame, CA, USA) and imaged at 1000× magnification using a fluorescence Olympus BX51 microscope (Tokyo, Japan) with appropriate filter (495–519 nM). Sperm heads were either scored as CMA3 positive (bright green staining) or CMA3 negative (dull green staining), with at least 200 sperm scored for each male. Epididymal sperm samples from *Zfy* DKO males contain testicular cells other than spermatozoa; these cells were not considered in counts.

Aniline blue staining was performed on sperm from all genotypes following an established technique [77]. Epididymal spermatozoa were released into HEPES-CZB medium, and the sperm suspension was spread on slides and air-dried for 20 min at 37 °C. Slides were then fixed in 3% glutaraldehyde in PBS for 30 min at room temperature, incubated in aniline blue staining solution (5% aniline blue in 4% acetic acid, pH = 3.5) for 5 min at room temperature, washed twice with distilled water, and air-dried for 20 min at 37 °C. The slides were mounted with Anti-Fluorescence Quenching Agent (Elabscience, Houston, TX, USA) and imaged at 1000× magnification using an Olympus BX51 microscope (Tokyo, Japan). Sperm heads were either scored as aniline positive (dark blue or purple staining) or aniline negative (light blue or clear staining), with at least 200 sperm scored for each male. Epididymal sperm samples from *Zfy* DKO males contain testicular cells other than spermatozoa, most of which stain positive for aniline blue; these cells were not considered in counts.

### TUNEL staining

The TUNEL assay for apoptotic cell detection in testis sections was performed using the One-step TUNEL In Situ Apoptosis Kit (E-CK-A320, Elabscience, USA) according to the manufacture instructions, with small modification to add Lectin-PNA staining (L32459, Invitrogen, USA). Images were captured with DP80 digital camera (Olympus) with Olympus BX51 microscope and processed with CellSens Imaging software (Olympus). *Zfy* DKO males were compared to wild-type XY controls and two kit controls, positive and negative, were included. The positive control was testis section from wild-type male treated with DNA-se. The negative control was testis section from a wild-type male processed without TdT enzyme. To select randomly 20 tubules an iPad & apple pencil were used, and a pdf format of the compiled cell images was uploaded to the program Goodnotes. A circle was drawn within the cross-section without looking, and a tubule close to the circle periphery and positioned in the top left corner was selected as tubule 1. The remaining 19 tubules were selected by following the circular spiral motion clockwise, within the circle.

### Nuclear protein extraction for protamine analyses

PRM1/PRM2 protamine ratios were determined following procedures of nuclear proteins extraction and analysis as previously described [78]. Briefly, mouse sperm pellets were resuspended in 0.5% Triton X-100, 20 mM Tris–HCl (pH 8), 2 mM MgCl_2_. After centrifugation at 8900 × *g* 5 min at 4 °C, the sediment was resuspended in MilliQ H_2_O with 1 mM PMSF, centrifuging at the same conditions. Chromatin was denatured by resuspending pellets in 20 mM EDTA, 1 mM PMSF, 100 mM Tris-HCl pH8 and adding 1 volume of 575 mM DTT in 6 M GuHCl prior vortexing. After adding final 0.8% 4-vynilpyridine, the solution was incubated at 37 °C for 30 min protected from light to disrupt cysteine disulfide bonds, vortexing every 5 min. Chromatin was precipitated by adding 5 volumes of cold ethanol and incubating 1 h at −20 °C, followed by centrifugation 12,900 × *g* for 15 min at 4 °C. Basic nuclear proteins were extracted from DNA by incubating with 0.5 M HCl at 37 °C and recovered in the supernatant after centrifugation at 17,530 × *g* for 10 min at 4 °C. Proteins were precipitated with 20% trichloroacetic acid (TCA) on ice. Protein extracts were washed twice with 1% β-mercaptoethanol in acetone and air-dried before being resuspended in 5.5 M urea, 20% β-mercaptoethanol, 5% acetic acid. For in-gel quantification, purified extracts corresponding to 250,000–700,000 spermatozoa were run by acetic acid urea polyacrylamide gel electrophoresis (AU-PAGE). Gels were stained with SafeStain SimplyBlue™ (#LC6060, Thermo Fisher Scientific, Waltham, MA, USA) and scanned using an iBright™ FL1500 Imaging System (Thermo Fisher Scientific). Band density corresponding to mouse PRM1 and PRM2 was quantified using the iBright Analysis Software 1.8.1 to calculate PRM1/PRM2 ratios. Statistical analyses (Student *t*-test) and plotting were made on GraphPad Prism 10.

### Acidic western blot

For protamine Western Blot detection, we proceeded as previously described [61], with minor modifications. Antibodies are listed in Table S10. Sperm nuclear protein extracts corresponding to 2.1 million spermatozoa from one XY control and a pool of 3 *Zfy* DKO sperm samples were loaded into AU-PAGE and separated as detailed before. Proteins were transferred from the acid-urea gel towards the negative pole onto a 0.45-µm pore size nitrocellulose membrane (88018, ThermoFisher) for 5.5 h at 4 °C using an acidic transfer buffer consisting of 0.9 mM acetic acid. Membrane was blocked with 5% non-fat dry milk in PBST 0.1% for 1 h and incubated overnight at 4 °C with primary antibody against PRM2 (#MAb-Hup2B, Briar Patch Biosciences) diluted 1:1000 in blocking buffer. Secondary antibody (Goat anti‐mouse HRP, #31430, ThermoFisher) incubation was performed for 2 h at room temperature. Visualization was performed with Super-Signal West Pico Plus® ECL (#34580, ThermoFisher) on the iBright™ FL1500 Imaging System.

### Immunofluorescence

Immunofluorescence staining was performed on 5 µm thick sections of mice testes fixed in 4% buffered paraformaldehyde (PFA) and embedded in paraffin. Following deparaffinization and rehydration, and antigen retrieval, sections were permeabilized for 20 min in PBS containing 0.3% Triton X-100. To block endogenous peroxidase activity, tissue sections were incubated with 3% hydrogen peroxide solution for 20 min at room temperature. Blocking was then performed for 1 h at room temperature in Tris-buffered saline with 0.05% Tween-20 (TBST), supplemented with 0.1% Triton X-100, 2% horse serum, and 3% bovine serum albumin. Primary antibodies were diluted in blocking buffer and applied to the sections. Negative controls were prepared by omitting the primary antibody. Sections were incubated overnight at 4 °C. After incubation, sections were washed three times with TBST and fluorochrome-conjugated secondary antibodies, diluted in blocking buffer, were applied to the sections and incubated for 1 h at RT in a humidified chamber protected from light. Sections were then washed three times with TBST. Lectin PNA From *Arachis hypogaea* (peanut), Alexa Fluor^™^ 594 Conjugate was used (1:500; Invitrogen, Cat# L32459) to label the acrosomal region and identify the stages of seminiferous tubules, as described in [79]. Nuclei were counterstained using Hoechst included in the mounting medium, which was freshly prepared by mixing PBS, glycerol, and nuclear stain, and protected from light. Stained slides were mounted and visualized after 15 min. using a fluorescence microscope (Olympus BX51) equipped with a digital camera. Antibodies are listed in Table S10.

#### Statistics

Statistical parameters including the statistical tests used, values of *n*, and statistical significance are reported in the figure legends. Results are expressed as average ± standard error of the mean (SEM) or standard deviation (SDev). Differences were considered significant when the *p* value was <0.05 (*), <0.01 (**), <0.001 (***), or <0.0001 (****). If data were expressed as percentages, the percentages were transformed to angles for statistical analyses. Basic computations were done using GraphPad Prism (Prism 10, version 10.4.2).

Data shown in Fig. 2D was analyzed using Wilcoxon test adjusted with Benjamini-Hochberg (BH) correction. Data shown in Figs. 2F, 3G, I, 4A, 5E, 6D, 7D, 8D, and S7 were analyzed by unpaired *t*-test. Data shown in Fig. 5H were analyzed by Fisher’s Exact test. Data shown in Fig. S5 were analyzed with 2-way ANOVA and post-hoc Holm-Šidak and unpaired *t*-test. Data presented in Tables S2–3, S6–7 and Fig. S8 show the results of HOMER analysis. HOMER uses cumulative hypergeometric distribution to detect enriched motifs. This statistical approach calculates the probability of observing any given number of target sequences with the motifs, assuming no relationship between the target sequence and motif. Data presented in Fig. 3D, E, Fig. 6A, Tables S4 and S8 show the results of GSEA analysis. GSEA uses a weighted Kolmogorov-Smirnov (WKS) test to calculate an enrichment score. *P*-values are determined through permutation testing, generating a null distribution of ES values, and comparing the observed ES to this distribution. Data shown in Fig. 2A, B, E, S2, S4 and S8C were analyzed with DESeq2 and edgeR. DESeq2 and edgeR are R packages used for differential expression analysis using default parameters. The process involves modeling the raw read counts, estimating dispersions, and then performing statistical hypothesis testing. The statistical tests that were used are a modified likelihood-ratio test (LRT) against a fold change threshold (1.5) following by a BH correction. The data shown in Fig. 2D were analyzed with Wilcoxon test adjusted with Benjamin-Hochberg correction.

The data shown in Fig. 3A–C, Fig. 5A–D, and Table S5 were obtained using the EnrichR function in ClusterProfiler, which uses a hypergeometric test with an adjusted *p* value to determine if a set of genes is enriched within a particular functional category. PCA representations were produced with FactomineR package. Data shown in Fig. S6 were analyzed using z-score ratio.

The data shown in Fig. 7A–C and S12 were produced using rMATS and Whippet. rMATS calculates Percent Spliced In (PSI) values for each splicing event and applies a LRT to assess the significance of DS between conditions. The null hypothesis (assuming no splicing difference between conditions) is compared against the alternative model (condition-specific splicing changes), with *p* values derived from the LRT. To account for multiple testing, *p* values are adjusted using the BH method, giving the False Discovery Rate (FDR). AS events with |deltaPSI| ≥ 0.1 and FDR < 0.05 were considered statistically significant. Whippet uses a Bayesian inference framework to estimate splicing efficiency. DS is assessed using posterior probabilities, where events with P(DS) > 0.95 (this can be modulated by the user and indicates a 95% probability of DS) and |deltaPSI| ≥ 0.1 were used for significant AS selection. Data shown in Fig. S13 were produced using EnrichGO() function, which uses Fisher’s exact test to assess whether specific GO terms are significantly overrepresented in the gene set of interest compared to a background of all annotated genes. The resulting *p* values were adjusted for multiple testing using the BH method, with an adjusted *p* value (FDR) threshold of <0.05 considered statistically significant.

## Supplementary information

Supplementary Material

Original Data

Dataset File S1

Dataset File S1

Supplemental Movie M1

Supplemental Movie M2

## Data Availability

All data analyses for this study are included in this published article and its supplementary information files. Raw FASTQ files from the bulk transcriptome analysis are available at EBI array express repository (reference number: E-MTAB-14570) and GEO repository (accession number: GSE281037). The fully reproducible and documented analysis for RNA‐Seq is available on github at https://github.com/ManonCoulee/ZFY_Holmlund_2024. The fully reproducible and documented pathway enrichment analysis for RNA-seq is available at https://github.com/Hayden-Holmlund/Holmlund-2024-ZFY-RNA-seq.

## References

[1] Holmlund H, Yamauchi Y, Ruthig VA, Cocquet J, Ward MA. Return of the forgotten hero: the role of Y chromosome-encoded Zfy in male reproduction. Mol Hum Reprod. 2023;29:gaad025. 37354519 10.1093/molehr/gaad025 PMC10695432

[2] Ni W, Perez AA, Schreiner S, Nicolet CM, Farnham PJ. Characterization of the ZFX family of transcription factors that bind downstream of the start site of CpG island promoters. Nucleic Acids Res. 2020;48:5986–6000. 32406922 10.1093/nar/gkaa384 PMC7293018

[3] Decarpentrie F, Vernet N, Mahadevaiah SK, Longepied G, Streichemberger E, Aknin-Seifer I, et al. Human and mouse ZFY genes produce a conserved testis-specific transcript encoding a zinc finger protein with a short acidic domain and modified transactivation potential. Hum Mol Genet. 2012;21:2631–45. 22407129 10.1093/hmg/dds088 PMC3363334

[4] Holzel M, Kohlhuber F, Schlosser I, Holzel D, Luscher B, Eick D. Myc/Max/Mad regulate the frequency but not the duration of productive cell cycles. EMBO Rep. 2001;2:1125–32. 11743027 10.1093/embo-reports/kve251 PMC1084169

[5] Levens D. Disentangling the MYC web. Proc Natl Acad Sci USA. 2002;99:5757–9. 11983876 10.1073/pnas.102173199 PMC122847

[6] San Roman AK, Skaletsky H, Godfrey AK, Bokil NV, Teitz L, Singh I, et al. The human Y and inactive X chromosomes similarly modulate autosomal gene expression. Cell Genom. 2024;4:100462. 10.1016/j.xgen.2023.100462 PMC10794785 38190107

[7] Blanton LV, San Roman AK, Wood G, Buscetta A, Banks N, Skaletsky H, et al. Stable and robust Xi and Y transcriptomes drive cell-type-specific autosomal and Xa responses in vivo and in vitro in four human cell types. Cell Genom. 2024;4:100628. 39111319 10.1016/j.xgen.2024.100628 PMC11480847

[8] Turner JM. Meiotic sex chromosome inactivation. Development. 2007;134:1823–31. 17329371 10.1242/dev.000018

[9] Vernet N, Mahadevaiah SK, de Rooij DG, Burgoyne PS, Ellis PJI. Zfy genes are required for efficient meiotic sex chromosome inactivation (MSCI) in spermatocytes. Hum Mol Genet. 2016;25:5300–10. 27742779 10.1093/hmg/ddw344 PMC5418838

[10] Royo H, Polikiewicz G, Mahadevaiah SK, Prosser H, Mitchell M, Bradley A, et al. Evidence that meiotic sex chromosome inactivation is essential for male fertility. Curr Biol. 2010;20:2117–23. 21093264 10.1016/j.cub.2010.11.010

[11] Vernet N, Mahadevaiah SK, Ojarikre OA, Longepied G, Prosser HM, Bradley A, et al. The Y-encoded gene zfy2 acts to remove cells with unpaired chromosomes at the first meiotic metaphase in male mice. Curr Biol. 2011;21:787–93. 21530259 10.1016/j.cub.2011.03.057 PMC3176893

[12] Vernet N, Mahadevaiah SK, Yamauchi Y, Decarpentrie F, Mitchell MJ, Ward MA, et al. Mouse Y-linked Zfy1 and Zfy2 are expressed during the male-specific interphase between meiosis I and meiosis II and promote the 2nd meiotic division. PLoS Genet. 2014;10:e1004444. 24967676 10.1371/journal.pgen.1004444 PMC4072562

[13] Vernet N, Mahadevaiah SK, Decarpentrie F, Longepied G, de Rooij DG, Burgoyne PS, et al. Mouse Y-encoded transcription factor Zfy2 is essential for sperm head remodelling and sperm tail development. PLoS ONE. 2016;11:e0145398. 26765744 10.1371/journal.pone.0145398 PMC4713206

[14] Yamauchi Y, Matsumura T, Bakse J, Holmlund H, Blanchet G, Carrot E, et al. Loss of mouse Y chromosome gene Zfy1 and Zfy2 leads to spermatogenesis impairment, sperm defects, and infertility. Biol Reprod. 2022;106:1312–26. 35293998 10.1093/biolre/ioac057 PMC9199016

[15] Yamauchi Y, Riel JM, Ruthig V, Ward MA. Mouse Y-encoded transcription factor Zfy2 is essential for sperm formation and function in assisted fertilization. PLoS Genet. 2015;11:e1005476. 26719889 10.1371/journal.pgen.1005476 PMC4697804

[16] Bugno-Poniewierska M, Raudsepp T. Horse clinical cytogenetics: recurrent themes and novel findings. Animals. 2021;11:831. 33809432 10.3390/ani11030831 PMC8001954

[17] Ruiz AJ, Castaneda C, Raudsepp T, Tibary A. Azoospermia and Y chromosome-autosome translocation in a friesian stallion. J Equine Vet Sci. 2019;82:102781. 31732110 10.1016/j.jevs.2019.07.002

[18] Barasc H, Mary N, Letron R, Calgaro A, Dudez AM, Bonnet N, et al. Y-autosome translocation interferes with meiotic sex inactivation and expression of autosomal genes: a case study in the pig. Sex Dev. 2012;6:143–50. 21921590 10.1159/000331477

[19] Arakawa Y, Nishida-Umehara C, Matsuda Y, Sutou S, Suzuki H. X-chromosomal localization of mammalian Y-linked genes in two XO species of the Ryukyu spiny rat. Cytogenet Genome Res. 2002;99:303–9. 12900579 10.1159/000071608

[20] Mulugeta E, Wassenaar E, Sleddens-Linkels E, van IWF, Heard E, Grootegoed JA, et al. Genomes of Ellobius species provide insight into the evolutionary dynamics of mammalian sex chromosomes. Genome Res. 2016;26:1202–10. 27510564 10.1101/gr.201665.115 PMC5052041

[21] Waters PD, Ruiz-Herrera A. Meiotic executioner genes protect the Y from extinction. Trends Genet. 2020;36:728–38. 32773168 10.1016/j.tig.2020.06.008

[22] Ruiz-Herrera A, Waters PD. Fragile, unfaithful and persistent Ys-on how meiosis can shape sex chromosome evolution. Heredity. 2022;129:22–30. 35459933 10.1038/s41437-022-00532-2 PMC9273583

[23] Vernet N, Mahadevaiah SK, Ellis PJ, de Rooij DG, Burgoyne PS. Spermatid development in XO male mice with varying Y chromosome short-arm gene content: evidence for a Y gene controlling the initiation of sperm morphogenesis. Reproduction. 2012;144:433–45. 22869781 10.1530/REP-12-0158 PMC3464043

[24] Wu T, Hu E, Xu S, Chen M, Guo P, Dai Z, et al. clusterProfiler 4.0: a universal enrichment tool for interpreting omics data. Innov (Camb). 2021;2:100141. 10.1016/j.xinn.2021.100141 PMC8454663 34557778

[25] Subramanian A, Tamayo P, Mootha VK, Mukherjee S, Ebert BL, Gillette MA, et al. Gene set enrichment analysis: a knowledge-based approach for interpreting genome-wide expression profiles. Proc Natl Acad Sci USA. 2005;102:15545–50. 16199517 10.1073/pnas.0506580102 PMC1239896

[26] Moritz L, Hammoud SS. The art of packaging the sperm genome: molecular and structural basis of the histone-to-protamine exchange. Front Endocrinol. 2022;13:895502. 10.3389/fendo.2022.895502 PMC9258737 35813619

[27] Heinz S, Benner C, Spann N, Bertolino E, Lin YC, Laslo P, et al. Simple combinations of lineage-determining transcription factors prime cis-regulatory elements required for macrophage and B cell identities. Mol Cell. 2010;38:576–89. 20513432 10.1016/j.molcel.2010.05.004 PMC2898526

[28] Abramson J, Adler J, Dunger J, Evans R, Green T, Pritzel A, et al. Accurate structure prediction of biomolecular interactions with AlphaFold 3. Nature. 2024;630:493–500. 38718835 10.1038/s41586-024-07487-w PMC11168924

[29] Lolis D, Georgiou I, Syrrou M, Zikopoulos K, Konstantelli M, Messinis I. Chromomycin A3-staining as an indicator of protamine deficiency and fertilization. Int J Androl. 1996;19:23–27. 8698534 10.1111/j.1365-2605.1996.tb00429.x

[30] Ribas-Maynou J, Llavanera M, Mateo-Otero Y, Garcia-Bonavila E, Delgado-Bermudez A, Yeste M. Direct but not indirect methods correlate the percentages of sperm with altered chromatin to the intensity of chromatin damage. Front Vet Sci. 2021;8:719319. 34746276 10.3389/fvets.2021.719319 PMC8570191

[31] Pourmasumi S, Khoradmehr A, Rahiminia T, Sabeti P, Talebi AR, Ghasemzadeh J. Evaluation of sperm chromatin integrity using aniline blue and toluidine blue staining in infertile and normozoospermic men. J Reprod Infertil. 2019;20:95–101. 31058054 PMC6486564

[32] Sellami A, Chakroun N, Ben Zarrouk S, Sellami H, Kebaili S, Rebai T, et al. Assessment of chromatin maturity in human spermatozoa: useful aniline blue assay for routine diagnosis of male infertility. Adv Urol. 2013;2013:578631. 24198830 10.1155/2013/578631 PMC3808709

[33] Corzett M, Mazrimas J, Balhorn R. Protamine 1: protamine 2 stoichiometry in the sperm of eutherian mammals. Mol Reprod Dev. 2002;61:519–27. 11891924 10.1002/mrd.10105

[34] de Mateo S, Gazquez C, Guimera M, Balasch J, Meistrich ML, Ballesca JL, et al. Protamine 2 precursors (Pre-P2), protamine 1 to protamine 2 ratio (P1/P2), and assisted reproduction outcome. Fertil Steril. 2009;91:715–22. 18314125 10.1016/j.fertnstert.2007.12.047

[35] Arevalo L, Esther Merges G, Schneider S, Schorle H. Protamines: lessons learned from mouse models. Reproduction. 2022;164:R57–R74. 35900356 10.1530/REP-22-0107

[36] Oliva R. Protamines and male infertility. Hum Reprod Update. 2006;12:417–35. 16581810 10.1093/humupd/dml009

[37] Rathke C, Baarends WM, Awe S, Renkawitz-Pohl R. Chromatin dynamics during spermiogenesis. Biochim Biophys Acta. 2014;1839:155–68. 24091090 10.1016/j.bbagrm.2013.08.004

[38] Soumillon M, Necsulea A, Weier M, Brawand D, Zhang X, Gu H, et al. Cellular source and mechanisms of high transcriptome complexity in the mammalian testis. Cell Rep. 2013;3:2179–90. 23791531 10.1016/j.celrep.2013.05.031

[39] Barral S, Morozumi Y, Tanaka H, Montellier E, Govin J, de Dieuleveult M, et al. Histone variant H2A.L.2 guides transition protein-dependent protamine assembly in male germ cells. Mol Cell. 2017;66:89–101.e108. 28366643 10.1016/j.molcel.2017.02.025

[40] Molaro A, Wood AJ, Janssens D, Kindelay SM, Eickbush MT, Wu S, et al. Biparental contributions of the H2A.B histone variant control embryonic development in mice. PLoS Biol. 2020;18:e3001001. 33362208 10.1371/journal.pbio.3001001 PMC7757805

[41] Moretti C, Serrentino ME, Ialy-Radio C, Delessard M, Soboleva TA, Tores F, et al. SLY regulates genes involved in chromatin remodeling and interacts with TBL1XR1 during sperm differentiation. Cell Death Differ. 2017;24:1029–44. 28475176 10.1038/cdd.2017.32 PMC5442469

[42] Riel JM, Yamauchi Y, Sugawara A, Li HY, Ruthig V, Stoytcheva Z, et al. Deficiency of the multi-copy mouse Y gene Sly causes sperm DNA damage and abnormal chromatin packaging. J Cell Sci. 2013;126:803–13. 23178944 10.1242/jcs.114488 PMC3619810

[43] Yamauchi Y, Riel JM, Stoytcheva Z, Burgoyne PS, Ward MA. Deficiency in mouse Y chromosome long arm gene complement is associated with sperm DNA damage. Genome Biol. 2010;11:R66. 20573212 10.1186/gb-2010-11-6-r66 PMC2911114

[44] Mazeyrat S, Saut N, Grigoriev V, Mahadevaiah SK, Ojarikre OA, Rattigan A, et al. A Y-encoded subunit of the translation initiation factor Eif2 is essential for mouse spermatogenesis. Nat Genet. 2001;29:49–53. 11528390 10.1038/ng717

[45] Yamauchi Y, Riel JM, Stoytcheva Z, Ward MA. Two Y genes can replace the entire Y chromosome for assisted reproduction in the mouse. Science. 2014;343:69–72. 24263135 10.1126/science.1242544 PMC3880637

[46] Nakasuji T, Ogonuki N, Chiba T, Kato T, Shiozawa K, Yamatoya K, et al. Complementary critical functions of Zfy1 and Zfy2 in mouse spermatogenesis and reproduction. PLoS Genet. 2017;13:e1006578. 28114340 10.1371/journal.pgen.1006578 PMC5287576

[47] Subrini J, Varsally W, Balsells IB, Bensberg M, Sioutas G, Ojarikre O, et al. Systematic identification of Y-chromosome gene functions in mouse spermatogenesis. Science. 2025;387:393–400. 39847625 10.1126/science.ads6495 PMC7617377

[48] Zhou H, Liu LH, Zhang H, Lei Z, Lan ZJ. Expression of zinc finger protein 105 in the testis and its role in male fertility. Mol Reprod Dev. 2010;77:511–20. 20186958 10.1002/mrd.21171 PMC3062163

[49] Sarkar RK, Sen Sharma S, Mandal K, Wadhwa N, Kunj N, Gupta A, et al. Homeobox transcription factor Meis1 is crucial to Sertoli cell mediated regulation of male fertility. Andrology. 2021;9:689–99. 33145986 10.1111/andr.12941

[50] Uchida A, Imaimatsu K, Suzuki H, Han X, Ushioda H, Uemura M, et al. SOX17-positive rete testis epithelium is required for Sertoli valve formation and normal spermiogenesis in the male mouse. Nat Commun. 2022;13:7860. 36543770 10.1038/s41467-022-35465-1 PMC9772346

[51] Aubrey BJ, Kelly GL, Janic A, Herold MJ, Strasser A. How does p53 induce apoptosis and how does this relate to p53-mediated tumour suppression? Cell Death Differ. 2018;25:104–13. 29149101 10.1038/cdd.2017.169 PMC5729529

[52] Ramesh S, Qi XJ, Wildey GM, Robinson J, Molkentin J, Letterio J, et al. TGF beta-mediated BIM expression and apoptosis are regulated through SMAD3-dependent expression of the MAPK phosphatase MKP2. EMBO Rep. 2008;9:990–7. 18704116 10.1038/embor.2008.158 PMC2572119

[53] Ramesh S, Wildey GM, Howe PH. Transforming growth factor beta (TGFbeta)-induced apoptosis: the rise & fall of Bim. Cell Cycle. 2009;8:11–17. 19106608 10.4161/cc.8.1.7291 PMC3191464

[54] Ferreira A, Pereira F, Reis C, Oliveira MJ, Sousa MJ, Preto A. Crucial role of oncogenic KRAS mutations in apoptosis and autophagy regulation: therapeutic implications. Cells. 2022;11:2183. 35883626 10.3390/cells11142183 PMC9319879

[55] Soh YQ, Alfoldi J, Pyntikova T, Brown LG, Graves T, Minx PJ, et al. Sequencing the mouse y chromosome reveals convergent gene acquisition and amplification on both sex chromosomes. Cell. 2014;159:800–13. 25417157 10.1016/j.cell.2014.09.052 PMC4260969

[56] Zuo Z, Billings T, Walker M, Petkov PM, Fordyce PM, Stormo GD. On the dependent recognition of some long zinc finger proteins. Nucleic Acids Res. 2023;51:5364–76. 36951113 10.1093/nar/gkad207 PMC10287918

[57] Chatot CL, Lewis JL, Torres I, Ziomek CA. Development of 1-cell embryos from different strains of mice in CZB medium. Biol Reprod. 1990;42:432–40. 2111184 10.1095/biolreprod42.3.432

[58] Chatot CL, Ziomek CA, Bavister BD, Lewis JL, Torres I. An improved culture medium supports development of random-bred 1-cell mouse embryos in vitro. J Reprod Fertil. 1989;86:679–88. 2760894 10.1530/jrf.0.0860679

[59] Holmlund H, Yamauchi Y, Durango G, Fujii W, Ward MA. Two acquired mouse Y chromosome-linked genes, Prssly and Teyorf1, are dispensable for male fertility double dagger. Biol Reprod. 2022;107:752–64. 35485405 10.1093/biolre/ioac084 PMC9476217

[60] Quinn P. Enhanced results in mouse and human embryo culture using a modified human tubal fluid medium lacking glucose and phosphate. J Assist Reprod Genet. 1995;12:97–105. 7670281 10.1007/BF02211377

[61] Blanco M, El Khattabi L, Gobe C, Crespo M, Coulee M, de la Iglesia A, et al. DOT1L regulates chromatin reorganization and gene expression during sperm differentiation. EMBO Rep. 2023;24:e56316. 10.15252/embr.202256316 PMC10240200 37099396

[62] Mootha VK, Lindgren CM, Eriksson KF, Subramanian A, Sihag S, Lehar J, et al. PGC-1alpha-responsive genes involved in oxidative phosphorylation are coordinately downregulated in human diabetes. Nat Genet. 2003;34:267–73. 12808457 10.1038/ng1180

[63] Yu G, Wang LG, Han Y, He QY. clusterProfiler: an R package for comparing biological themes among gene clusters. OMICS. 2012;16:284–7. 22455463 10.1089/omi.2011.0118 PMC3339379

[64] Chen EY, Tan CM, Kou Y, Duan Q, Wang Z, Meirelles GV, et al. Enrichr: interactive and collaborative HTML5 gene list enrichment analysis tool. BMC Bioinforma. 2013;14:128. 10.1186/1471-2105-14-128 PMC3637064 23586463

[65] Kuleshov MV, Jones MR, Rouillard AD, Fernandez NF, Duan Q, Wang Z, et al. Enrichr: a comprehensive gene set enrichment analysis web server 2016 update. Nucleic Acids Res. 2016;44:W90–97. 27141961 10.1093/nar/gkw377 PMC4987924

[66] Shen S, Park JW, Lu ZX, Lin L, Henry MD, Wu YN, et al. rMATS: robust and flexible detection of differential alternative splicing from replicate RNA-Seq data. Proc Natl Acad Sci USA. 2014;111:E5593–5601. 25480548 10.1073/pnas.1419161111 PMC4280593

[67] Sterne-Weiler T, Weatheritt RJ, Best AJ, Ha KCH, Blencowe BJ. Efficient and accurate quantitative profiling of alternative splicing patterns of any complexity on a laptop. Mol Cell. 2018;72:187–200.e186. 30220560 10.1016/j.molcel.2018.08.018

[68] Quinlan AR, Hall IM. BEDTools: a flexible suite of utilities for comparing genomic features. Bioinformatics. 2010;26:841–2. 20110278 10.1093/bioinformatics/btq033 PMC2832824

[69] Garcia I, Larcombe L. Exploring the perturbation of biological processes caused by gene upregulation using knock-in transfection, transcriptomics and bioinformatics analysis. Target identification and validation in drug discovery: methods and protocols. 3rd ed. Germany: Springer Nature; 2024. 10.1007/978-1-0716-4418-8_7 40163301

[70] Andrews S FastQC: a quality control tool for high throughput sequence data [Online]. 2010. https://www.bioinformaticsbabrahamacuk/projects/fastqc/.

[71] Kim D, Langmead B, Salzberg SL. HISAT: a fast spliced aligner with low memory requirements. Nat Methods. 2015;12:357–60. 25751142 10.1038/nmeth.3317 PMC4655817

[72] Danecek P, Bonfield JK, Liddle J, Marshall J, Ohan V, Pollard MO, et al. Twelve years of SAMtools and BCFtools. Gigascience. 2021;10:giab008. 33590861 10.1093/gigascience/giab008 PMC7931819

[73] Liao Y, Smyth GK, Shi W. featureCounts: an efficient general purpose program for assigning sequence reads to genomic features. Bioinformatics. 2014;30:923–30. 24227677 10.1093/bioinformatics/btt656

[74] Love MI, Huber W, Anders S. Moderated estimation of fold change and dispersion for RNA-seq data with DESeq2. Genome Biol. 2014;15:550. 25516281 10.1186/s13059-014-0550-8 PMC4302049

[75] Milacic M, Beavers D, Conley P, Gong C, Gillespie M, Griss J, et al. The reactome pathway knowledgebase 2024. Nucleic Acids Res. 2024;52:D672–D678. 37941124 10.1093/nar/gkad1025 PMC10767911

[76] Simoes R, Feitosa WB, Mendes CM, Marques MG, Nicacio AC, de Barros FR, et al. Use of chromomycin A3 staining in bovine sperm cells for detection of protamine deficiency. Biotech histochem. 2009;84:79–83. 19306222 10.1080/10520290902843595

[77] Franken DR, Franken CJ, de la Guerre H, de Villiers A. Normal sperm morphology and chromatin packaging: comparison between aniline blue and chromomycin A3 staining. Andrologia. 1999;31:361–6. 10643511 10.1046/j.1439-0272.1999.00290.x

[78] Soler-Ventura A, Castillo J, de la Iglesia A, Jodar M, Barrachina F, Ballesca JL, et al. Mammalian sperm protamine extraction and analysis: a step-by-step detailed protocol and brief review of protamine alterations. Protein Pept Lett. 2018;25:424–33. 29651936 10.2174/0929866525666180412155205

[79] Ahmed EA, de Rooij DG. Staging of mouse seminiferous tubule cross-sections. Methods Mol Biol. 2009;558:263–77. 19685330 10.1007/978-1-60761-103-5_16

[80] Gan H, Wen L, Liao S, Lin X, Ma T, Liu J, et al. Dynamics of 5-hydroxymethylcytosine during mouse spermatogenesis. Nat Commun. 2013;4:1995. 23759713 10.1038/ncomms2995

[81] Green CD, Ma Q, Manske GL, Shami AN, Zheng X, Marini S, et al. A comprehensive roadmap of murine spermatogenesis defined by single-cell RNA-seq. Dev Cell. 2018;46:651–67.e610. 30146481 10.1016/j.devcel.2018.07.025 PMC6713459

[82] Green KA, Franasiak JM, Werner MD, Tao X, Landis JN, Scott RT Jr., et al. Cumulus cell transcriptome profiling is not predictive of live birth after in vitro fertilization: a paired analysis of euploid sibling blastocysts. Fertil Steril. 2018;109:460–466.e462. 29428306 10.1016/j.fertnstert.2017.11.002

