# Correction: Large-scale transcriptomic analyses reveal downstream target genes of ZFY1 and ZFY2 transcription factors in male germ cells

**DOI:** 10.1038/s41418-025-01606-4

**Published:** 2025-11-04

**Authors:** Hayden Holmlund, Manon Coulée, Yasuhiro Yamauchi, Benazir Yarbabaeva, Muhammetnur Tekayev, Isabella R. Garcia, Olivier U. Feudjio, Alberto de la Iglesia, Lee Larcombe, Peter J. I. Ellis, Julie Cocquet, Monika A. Ward

**Affiliations:** 1 Yanagimachi Institute for Biogenesis Research, John A. Burns School of Medicine, University of Hawaii, Honolulu, HI USA; 2 Institut Cochin, Université Paris Cité, INSERM, CNRS, Paris, France; 3 School of Biosciences, University of Kent, Canterbury, UK; 4 ADLIN Science, Evry, Cedex France; 5 Apexomic, Stevenage Bioscience Catalyst, Stevenage, UK

**Keywords:** Gene expression, Cell biology, Chromatin, Development

Correction to: *Cell Death & Differentiation* 10.1038/s41418-025-01569-6, published online 27 August 2025

In this article the author name “Olivier U. Feudijo” should read “Olivier U. Feudijo”.

The original article has been corrected.

