# Supplementary material for: Large-scale transcriptomic analyses reveal downstream target genes of ZFY1 and ZFY2 transcription factors in male germ cells
Source: Cell Death Differ. 2025 Aug 27;33(2):392–410. doi: 10.1038/s41418-025-01569-6 (PMC12881418; doi:10.1038/s41418-025-01569-6)
Supplement: Supplementary file 1 — Supplementary Material [file 41418_2025_1569_MOESM1_ESM.pdf]

Large-scale transcriptomic analyses reveal downstream target genes of ZFY1 and ZFY2 transcription factors in male germ cells

Hayden Holmlund[1], Manon Coulée[2], Yasuhiro Yamauchi[1], Benazir Yarbabaeva[1], Muhammetnur Tekayev[1], Isabella R. Garcia[3], Olivier U. Feudijo[2,4], Alberto de la Iglesia[2], Lee Larcombe[5], Peter J. I. Ellis[3]
Julie Cocquet[2], Monika A. Ward[1,6]

Supplementary Material

Table S1. STAR stats for bulk RNA sequencing.

Table S2. Gene Ontology (GO) analysis of *Zfy* DKO upregulated DEGs.

Table S3. Gene Ontology (GO) analysis of of *Zfy2* KO upregulated DEGs.

Table S4. Gene Set Enrichment Analysis (GSEA) of *Zfy* KO Germ Cells.

Table S5. Pathway enrichment analysis (PEA) of *Zfy* DKO germ cell DEGs.

Table S6. Gene Ontology (GO) analysis of of *Zfy* DKO downregulated DEGs.

Table S7. Gene Ontology (GO) analysis of of *Zfy2* KO downregulated DEGs.

Table S8. Gene Set Enrichment Analysis (GSEA) using the mouse hallmark (mh.all) database.

Table S9. Primer sequences.

Table S10. Antibodies.

Figure S1. Comparison of transcriptomes for pilot and expanded experiment.

Figure S2. Significantly de-regulated genes in *Zfy* KO germ cells.

Figure S3: Correlation of differentially expressed genes (DEGs) between *Zfy* KO models.

Figure S4. Differentially expressed genes (DEGs) by chromosome.

Figure S5. qPCR validation of gene deregulation detected by RNA-seq.

Figure S6. Expression dynamics of X-Y linked genes.

Figure S7. Expression of down-regulated sex-linked genes in *Zfy1* KO germ cells.

Figure S8. Identifying potential binding partners for ZFY in RS.

Figure S9. Alphafold modeling of potential ZFY and ZFX binding motifs identified by MEA

Figure S10. Protamine content in sperm from *Zfy* KO males – untrimmed gel and blot

Figure S11. Immunofluorescence detection of BCL2L.

Figure S12. *Zfy* loss deregulates the splicing of hundreds of transcripts.

Figure S13. Enrichment analysis of genes subject to differential splicing.

Supplemental File S1. Enriched pathways identified by the GO analysis function of the HOMER tool.

Supplemental File S2. Concordantly regulated genes in mouse germ cells and transfected HEK293 cells.

Supplementary Movie M1: Exemplary motility of sperm from XY control.

Supplementary Movie M2: Exemplary motility of sperm from *Zfy* DKO male.

Supplementary References

Table S1. STAR stats for bulk RNA sequencing.

**Feature**	**1_XY_sc2**	**2_XY_sc2**	**3_XY_sc2**	**4_DKO_sc2**	**5_DKO_sc2**	**6_DKO_sc2**	**7_XY_rs**	**8_XY_rs**	**9_XY_rs**	**10_XY_sc1**	**11_XY_sc1**	**12_XY_sc1**
average_read_length	274	274	277	281	279	278	275	279	277	278	277	269
#mapped_splice_sites	30840410	29135886	32390875	26733666	35209874	33337355	47039125	25950128	28711597	23895274	31449323	27076717
#annotated_splice_sites	29879000	28293007	31454530	25959159	34057185	32233170	45593916	25151401	27825892	23265699	30578993	26336816
#sequenced_reads	36549347	33731633	38296057	30947376	41201935	42005998	51457857	28704819	30156680	33116282	42366764	41903179
#uniquely_mapped_reads	33051318	30504498	34836402	28164530	37377415	37792050	46352424	26110152	27312283	30055513	38369180	37232538
#multi_mapped_reads_kept	1267859	1146263	1248724	1103298	1468435	1481087	1819747	1012056	1070604	1162328	1479813	1651346
#multi_mapped_reads_discarded	125104	103371	108965	113622	155470	164844	181015	96275	90718	77894	105464	112827
#chimeric_reads	205794	171860	184168	181188	273113	289639	292161	171023	153563	146235	211942	235589

**Feature**	**13_2KO_sc1**	**14_2KO_sc1**	**15_2KO_sc1**	**16_2KO_sc2**	**17_2KO_sc2**	**18_2KO_sc2**	**19_2KO_rs**	**20_2KO_rs**	**21_2KO_rs**	**22_1KO_sc1**	**23_1KO_sc1**	**24_1KO_sc1**
average_read_length	273	275	271	283	279	276	275	274	271	276	277	276
#mapped_splice_sites	24320854	26509992	21990303	28081773	24625916	22378123	24189726	26800668	23408457	27816977	26509903	25042154
#annotated_splice_sites	23719024	25835573	21416955	27082636	23771624	21649795	23292826	25660869	22633643	27037992	25789816	24433000
#sequenced_reads	30485412	34263307	27746160	39259285	41493187	28182973	31891406	40804576	26678937	37105479	36592904	31947843
#uniquely_mapped_reads	27381079	30559232	24519258	35387708	35816310	25285729	28461416	36310114	23792574	33179077	32511288	28057070
#multi_mapped_reads_kept	915672	1109571	881476	1249798	1309080	922107	1124550	1538053	926304	1230090	1149613	941748
#multi_mapped_reads_discarded	59196	71945	76250	128307	133904	97565	136771	210357	97225	95026	85912	52152
#chimeric_reads	149474	238176	204103	281917	440656	202476	249329	387650	178250	182900	200109	111278

**Feature**	**25_1KO_sc2**	**26_1KO_sc2**	**27_1KO_sc2**	**28_1KO_rs**	**29_1KO_rs**	**30_1KO_rs**	**31_DKO_sc1**	**32_DKO_sc1**	**33_DKO_sc1**	**34_DKO_rs**	**35_DKO_rs**	**36_DKO_rs**
average_read_length	277	279	280	281	280	279	279	285	278	274	280	280
#mapped_splice_sites	27942520	32119872	40157670	30212176	33294503	30958405	28402868	28865963	30735534	28216519	39617584	34319112
#annotated_splice_sites	27080456	31065633	38868722	29238726	32171424	29945602	27678729	28070479	29995979	27301781	38215298	33194640
#sequenced_reads	34716718	36949178	48031093	35091061	36953824	34582422	37332807	42129770	36121791	33304951	46037300	37414629
#uniquely_mapped_reads	31369573	33468124	43743284	32053397	33515275	31206635	33706765	38297834	32571572	29572559	41858959	33679175
#multi_mapped_reads_kept	1114421	1231977	1546411	1151347	1270965	1167402	1235693	1367669	1188793	1259463	1675143	1355419
#multi_mapped_reads_discarded	115151	129832	142432	123171	139677	117367	76457	90334	65262	134222	205501	136014
#chimeric_reads	198347	235261	245681	218603	222093	191008	146388	208089	113597	225131	312269	241833

**Feature**	**S1_XY_sc1**	**S2_XY_sc1**	**S3_XY_sc1**	**S4_XY_rs**	**S5_XY_rs**	**S6_XY_rs**	**S7_DKO_sc1**	**S8_DKO sc1**	**S9_DKO_sc1**	**S10_DKO_rs**	**S11_DKO_rs**	**S12_DKO_rs**
average_read_length	288	287	287	287	288	287	288	289	287	288	288	286
#mapped_splice_sites	9269706	15518228	10451778	11361261	17067451	18616129	18937467	14177317	11618695	21981462	27640337	5220429
#annotated_splice_sites	9031629	15100001	10144336	10955816	16499438	17983998	18430271	13828391	11302547	21137842	26682674	5022603
#sequenced_reads	15167024	23109923	15626658	14266139	21626978	21850862	29153637	23164901	16152099	29965046	33347040	7145629
#uniquely_mapped_reads	14140398	21328139	14344233	13171585	19919029	20305019	26834868	21730041	14731646	27532416	31100593	6357276
#multi_mapped_reads_kept	584277	854623	581369	525236	833972	840838	1213248	980097	637016	1245025	1391856	301752
#multi_mapped_reads_discarded	31116	45928	24829	50500	75187	70103	60054	48576	24760	151884	162433	27448
#chimeric_reads	65623	91820	50711	90334	133173	117229	125093	122429	43452	235258	245383	42625

Samples S1-12 are data from the pilot experiment (batch 1, samples 1-36 are data from the expanded experiment (batch 2).

Table S2. Gene Ontology (GO) analysis of *Zfy* DKO upregulated DEGs.

	Zfy DKO RS UP BP			*Zfy* DKO RS UP CC			*Zfy* DKO RS UP MF		
**Rank**	**Term ID**	**Term**	**p-val**	**Term ID**	**Term**	**p-val**	**Term ID**	**Term**	**p-val**
1	GO:0090304	nucleic acid metabolic process	1.29E-28	GO:0005622	intracellular	7.52E-46	GO:0003676	nucleic acid binding	5.96E-29
2	GO:0043170	macromolecule metabolic process	8.68E-28	GO:0044424	intracellular part	7.52E-46	GO:1901363	heterocyclic compound binding	2.24E-27
3	GO:0044237	cellular metabolic process	1.42E-26	GO:0005634	nucleus	1.51E-41	GO:0097159	organic cyclic compound binding	3.47E-26
4	GO:0006725	cellular aromatic compound metabolic process	6.17E-26	GO:0044422	organelle part	1.53E-40	GO:0005488	binding	7.48E-21
5	GO:0006139	nucleobase-containing compound met process	1.04E-25	GO:0044446	intracellular organelle part	1.61E-39	GO:0003723	RNA binding	3.51E-16
6	GO:0046483	heterocycle metabolic process	3.53E-25	GO:0043226	organelle	5.60E-39	GO:0003677	DNA binding	2.25E-14
7	GO:0034641	cellular nitrogen compound metabolic process	1.58E-24	GO:0043229	intracellular organelle	1.66E-38	GO:0005515	protein binding	2.20E-13
8	GO:0008152	metabolic process	4.91E-24	GO:0044428	nuclear part	1.95E-36	GO:0005524	ATP binding	1.22E-12
9	GO:0006807	nitrogen compound metabolic process	2.10E-23	GO:0070013	intracellular organelle lumen	5.64E-36	GO:0043167	ion binding	2.01E-12
10	GO:0071840	cellular component organization or biogenesis	1.08E-22	GO:0043233	organelle lumen	6.08E-36	GO:0003682	chromatin binding	4.65E-12
11	GO:0071704	organic substance metabolic process	1.13E-22	GO:0031974	membrane-enclosed lumen	6.08E-36	GO:0004386	helicase activity	7.01E-12
12	GO:1901360	organic cyclic compound metabolic process	1.20E-22	GO:0043231	intracellular membrane-bounded organelle	2.00E-35	GO:0032559	adenyl ribonucleotide binding	7.38E-12
13	GO:0010467	gene expression	2.61E-21	GO:0031981	nuclear lumen	7.83E-35	GO:0030554	adenyl nucleotide binding	1.49E-11
14	GO:0044238	primary metabolic process	1.86E-20	GO:0043227	membrane-bounded organelle	9.75E-35	GO:0035639	purine ribonucleoside triphosphate binding	1.26E-10
15	GO:0044260	cellular macromolecule metabolic process	5.11E-20	GO:0043228	non-membrane-bounded organelle	8.66E-28	GO:0008144	drug binding	1.72E-10
16	GO:0016070	RNA metabolic process	1.26E-19	GO:0043232	Intracell non-membrane-bounded organelle	3.04E-27	GO:0042623	ATPase activity, coupled	3.05E-10
17	GO:0016043	cellular component organization	1.37E-18	GO:0005654	nucleoplasm	6.63E-26	GO:0032553	ribonucleotide binding	4.40E-10
18	GO:0006996	organelle organization	3.55E-17	GO:0044464	cell part	2.40E-25	GO:0043168	anion binding	6.38E-10
19	GO:0006396	RNA processing	6.28E-17	GO:0005623	cell	2.92E-25	GO:0032555	purine ribonucleotide binding	8.36E-10
20	GO:0051276	chromosome organization	1.80E-16	GO:0032991	protein-containing complex	1.85E-23	GO:0016887	ATPase activity	8.59E-10
21	GO:0006974	cellular response to DNA damage stimulus	5.26E-15	GO:0005694	chromosome	1.49E-16	GO:0097367	carbohydrate derivative binding	1.41E-09
22	GO:0006261	DNA-dependent DNA replication	1.56E-12	GO:0005737	cytoplasm	1.27E-15	GO:0017076	purine nucleotide binding	1.54E-09
23	GO:0006325	chromatin organization	1.85E-12	GO:0044451	nucleoplasm part	8.82E-15	GO:0042393	histone binding	5.59E-09
24	GO:0022613	ribonucleoprotein complex biogenesis	1.98E-12	GO:0044427	chromosomal part	2.29E-13	GO:1901265	nucleoside phosphate binding	9.14E-09
25	GO:0006259	DNA metabolic process	1.27E-11	GO:0005829	cytosol	4.47E-13	GO:0000166	nucleotide binding	9.14E-09
26	GO:0006281	DNA repair	3.12E-10	GO:1990904	ribonucleoprotein complex	1.24E-11	GO:0008094	DNA-dependent ATPase activity	1.98E-08
27	GO:0006260	DNA replication	7.26E-10	GO:0044444	cytoplasmic part	1.93E-11	GO:0003697	single-stranded DNA binding	4.94E-08
28	GO:0019219	regulation of nucleobase-containing met proc	7.45E-10	GO:0016604	nuclear body	3.76E-11	GO:0003729	mRNA binding	1.66E-07
29	GO:0016071	mRNA metabolic process	8.75E-10	GO:0000228	nuclear chromosome	1.05E-10	GO:0003824	catalytic activity	1.99E-07
30	GO:0016569	covalent chromatin modification	1.39E-09	GO:0044454	nuclear chromosome part	5.79E-10	GO:0140097	catalytic activity, acting on DNA	4.77E-07

Top 30 pathways identified by the Gene Ontology analysis function in HOMER. Genes related to DNA packaging are shown in blue. BP = biological process, CC = cellular component, MF = molecular function.

Table S3. Gene Ontology (GO) analysis of *Zfy2* KO upregulated DEGs.

Rank	*Zfy2* KO RS UP BP			*Zfy2* KO RS UP CC			*Zfy2* KO RS UP MF		
	Term ID	Term	p-val	Term ID	Term	p-val	Term ID	Term	p-val
1	GO:0090304	nucleic acid metabolic process	2.25E-48	GO:0005622	intracellular	1.78E-51	GO:0003723	RNA binding	2.59E-31
2	GO:0006139	Nucleobase compound metabolic process	6.12E-43	GO:0044424	intracellular part	1.78E-51	GO:0003676	nucleic acid binding	7.55E-30
3	GO:0016070	RNA metabolic process	6.35E-42	GO:0044446	intracellular organelle part	3.40E-49	GO:1901363	heterocyclic compound binding	2.05E-27
4	GO:0006725	cellular aromatic compound metabolic process	3.49E-40	GO:0043229	intracellular organelle	4.73E-49	GO:0097159	organic cyclic compound binding	3.13E-25
5	GO:0046483	heterocycle metabolic process	7.79E-39	GO:0043226	organelle	1.10E-48	GO:0005488	binding	1.46E-14
6	GO:0006396	RNA processing	4.38E-38	GO:0044422	organelle part	8.95E-48	GO:0005524	ATP binding	1.84E-13
7	GO:0043170	macromolecule metabolic process	1.42E-37	GO:0005634	nucleus	1.38E-47	GO:0032559	adenyl ribonucleotide binding	6.68E-13
8	GO:0010467	gene expression	1.27E-36	GO:0043231	intracellular membrane-bounded organelle	3.78E-44	GO:0030554	adenyl nucleotide binding	1.50E-12
9	GO:0034641	cellular nitrogen compound metabolic process	1.54E-36	GO:0043227	membrane-bounded organelle	2.67E-41	GO:0140098	catalytic activity, acting on RNA	2.89E-12
10	GO:0044237	cellular metabolic process	1.17E-35	GO:0044428	nuclear part	1.05E-40	GO:0004386	helicase activity	8.62E-12
11	GO:1901360	organic cyclic compound metabolic process	4.80E-34	GO:0070013	intracellular organelle lumen	2.55E-39	GO:0043167	ion binding	6.65E-11
12	GO:0006807	nitrogen compound metabolic process	5.64E-34	GO:0043233	organelle lumen	2.78E-39	GO:0008144	drug binding	9.15E-11
13	GO:0044238	primary metabolic process	1.01E-28	GO:0031974	membrane-enclosed lumen	2.78E-39	GO:0042623	ATPase activity, coupled	1.86E-10
14	GO:0008152	metabolic process	1.16E-28	GO:0031981	nuclear lumen	5.90E-38	GO:0035639	purine ribonucleoside triphosphate binding	2.02E-10
15	GO:0071704	organic substance metabolic process	7.48E-28	GO:0005654	nucleoplasm	1.12E-33	GO:0032553	ribonucleotide binding	2.07E-10
16	GO:0044260	cellular macromolecule metabolic process	1.06E-27	GO:0043228	non-membrane-bounded organelle	4.47E-27	GO:0003727	single-stranded RNA binding	2.82E-10
17	GO:0016071	mRNA metabolic process	2.64E-27	GO:0043232	intracellular non-membrane-bounded organelle	6.65E-27	GO:0032555	purine ribonucleotide binding	5.32E-10
18	GO:0008380	RNA splicing	3.79E-26	GO:0044464	cell part	4.96E-26	GO:0017076	purine nucleotide binding	1.05E-09
19	GO:0006397	mRNA processing	6.52E-26	GO:0005623	cell	6.19E-26	GO:1901265	nucleoside phosphate binding	3.79E-09
20	GO:0050684	regulation of mRNA processing	2.24E-17	GO:0032991	protein-containing complex	5.14E-24	GO:0000166	nucleotide binding	3.79E-09
21	GO:0006996	organelle organization	3.14E-17	GO:0044451	nucleoplasm part	2.77E-23	GO:0004674	protein serine/threonine kinase activity	5.23E-09
22	GO:0071840	cellular component organization or biogenesis	5.52E-17	GO:0005737	cytoplasm	5.17E-21	GO:0016740	transferase activity	8.21E-09
23	GO:0034660	ncRNA metabolic process	7.84E-16	GO:1990904	ribonucleoprotein complex	1.93E-16	GO:0097367	carbohydrate derivative binding	2.12E-08
24	GO:0022613	ribonucleoprotein complex biogenesis	1.12E-15	GO:0005730	nucleolus	4.91E-16	GO:0016887	ATPase activity	2.31E-08
25	GO:0006974	cellular response to DNA damage stimulus	4.59E-15	GO:0016604	nuclear body	2.27E-15	GO:0008173	RNA methyltransferase activity	2.69E-08
26	GO:1903311	regulation of mRNA metabolic process	6.04E-15	GO:1902494	catalytic complex	5.56E-15	GO:0008168	methyltransferase activity	3.52E-08
27	GO:0034470	ncRNA processing	2.05E-14	GO:0044444	cytoplasmic part	5.32E-13	GO:0140097	catalytic activity, acting on DNA	3.57E-08
28	GO:0016043	cellular component organization	1.09E-13	GO:0005694	chromosome	3.54E-12	GO:0003697	single-stranded DNA binding	4.14E-08
29	GO:0000375	RNA splicing, via transesterification reactions	2.23E-13	GO:0016607	nuclear speck	1.62E-11	GO:0016741	transferase activity, transferring one-carbon groups	7.77E-08
30	GO:0000398	mRNA splicing, via spliceosome	2.23E-13	GO:0005681	spliceosomal complex	2.30E-11	GO:0003677	DNA binding	1.14E-07

Top 30 pathways identified by the Gene Ontology analysis function in HOMER. BP = biological process, CC = cellular component, MF = molecular function.

Table S4. Gene Set Enrichment Analysis (GSEA) of *Zfy* KO germ cells DEGs.

Cell Type	Data-base	Gene Set	Size	ES	NES	p-val	q-val	FWER p-val	Rank at Max	Expression*
Zfy DKO RS	BP	REGULATION_OF_RIBONUCLEASE_ACTIVITY	16	0.85	2.03	0.000	0.031	0.031	4434	UP
		RESPONSE_TO_PHENYLALANINE	25	-0.66	-2.02	0.000	0.000	0.000	12587	DOWN
	CC	NUCLEOSOME	120	0.69	2.09	0.000	0.001	0.001	6738	UP
		CHROMOSOME_CENTROMERIC_CORE_DOMAIN	25	0.80	2.03	0.000	0.003	0.006	4359	UP
	MF	STRUCTURAL_CONSTITUENT_OF_CHROMATIN	57	0.74	2.12	0.000	0.000	0.000	6691	UP
		PORE_FORMING_ACTIVITY	24	-0.61	-1.89	0.000	0.000	0.000	12661	DOWN
		TRACE_AMINE_RECEPTOR_ACTIVITY	15	-0.68	-1.83	0.000	0.000	0.001	12978	DOWN
Zfy DKO SC2	BP	RESPONSE_TO_PHENYLALANINE	25	-0.67	-1.75	0.000	0.000	0.006	12081	DOWN
	CC	PORE_FORMING_ACTIVITY	24	0.88	2.00	0.000	0.000	0.000	3612	UP
Zfy DKO SC1	MF	PORE_FORMING_ACTIVITY	24	0.93	2.31	0.000	0.000	0.000	1913	UP
		TRIPLET_CODON_AMINO_ACID_ADAPTOR_ACTIVITY	22	0.84	2.05	0.000	0.002	0.003	4542	UP
		TRACE_AMINE_RECEPTOR_ACTIVITY	15	-0.7	-1.87	0.0	0.0	0.0	7428	DOWN
		TYPE_I_INTERFERON_RECEPTOR_BINDING	24	-0.54	-1.5	0.0	1E-4	0.001	15206	DOWN
Zfy2 KO RS	MF	TRACE_AMINE_RECEPTOR_ACTIVITY	15	0.90	1.96	0.000	0.004	0.004	2172	UP
		TYPE_I_INTERFERON_RECEPTOR_BINDING	24	0.84	1.93	0.000	0.003	0.006	2413	UP
		PHEROMONE_ACTIVITY	18	-0.65	-1.77	0.0	0.0	0.0	13535	DOWN
Zfy2 KO SC2	BP	RESPONSE_TO_INTERFERON_ALPHA	32	0.79	1.76	0.000	0.046	0.046	7000	UP
	MF	PORE_FORMING_ACTIVITY	24	0.89	1.94	0.000	0.000	0.000	3507	UP
		CXCR_CHEMOKINE_RECEPTOR_BINDING	15	0.83	1.74	0.000	0.013	0.027	3642	UP
		BETA_2_MICROGLOBULIN_BINDING	28	0.78	1.72	0.000	0.011	0.032	5176	UP
		EXTRACELLULAR_MATRIX_BINDING	58	0.74	1.70	0.000	0.013	0.048	5680	UP
		TRACE_AMINE_RECEPTOR_ACTIVITY	15	-0.76	-2.02	0.000	0.000	0.000	7257	DOWN
		TRIPLET_CODON_AMINO_ACID_ADAPTOR_ACTIVITY	22	-0.67	-1.74	0.000	0.000	0.000	13577	DOWN
		PHEROMONE_ACTIVITY	18	-0.62	-1.54	0.000	0.002	0.005	13949	DOWN
Zfy2 KO SC1	CC	CATALYTIC_STEP_2_SPLICEOSOME	110	0.65	1.88	0.000	0.005	0.004	8469	UP
		SPLICEOSOMAL_COMPLEX	221	0.61	1.88	0.000	0.003	0.004	8577	UP
		PRERIBOSOME	112	0.61	1.79	0.000	0.018	0.039	7574	UP
	MF	PORE_FORMING_ACTIVITY	24	0.98	2.32	0.000	0.000	0.000	626	UP
		HELICASE_ACTIVITY	150	0.67	2.01	0.000	0.000	0.000	6466	UP
		ATP_DEPENDENT_ACTIVITY_ACTING_ON_DNA	115	0.67	1.95	0.000	0.000	0.001	6591	UP
		GOMF_CATALYTIC_ACTIVITY_ACTING_ON_RNA	386	0.60	1.93	0.000	0.000	0.001	7132	UP
		CATALYTIC_ACTIVITY_ACTING_ON_DNA	236	0.62	1.92	0.000	0.001	0.002	6708	UP
		CATALYTIC_ACTIVITY_ACTING_ON_A_TRNA	130	0.63	1.84	0.000	0.004	0.019	6213	UP
		NAD_RETINOL_DEHYDROGENASE_ACTIVITY	27	0.80	1.84	0.000	0.004	0.023	1122	UP
		PROTEIN_KINASE_C_BINDING	27	0.80	1.84	0.000	0.004	0.023	1122	UP
Zfy1 KO RS	BP	RESPONSE_TO_PHENYLALANINE	25	-0.60	-1.62	0.0	4E-4	0.001	15879	DOWN
	MF	TRIPLET_CODON_AMINO_ACID_ADAPTOR_ACTIVITY	22	-0.65	-1.76	0.0	0.0	0.0	14319	DOWN
		PHEROMONE_ACTIVITY	18	-0.62	-1.63	0.0	0.0	0.0	14553	DOWN
		PORE_FORMING_ACTIVITY	24	-0.62	-1.62	0.0	0.0	0.0	13401	DOWN
Zfy1 KO SC2	BP	SERTOLI_CELL_DIFFERENTIATION	29	0.81	1.83	0.000	0.028	0.028	4625	UP
	MF	PORE_FORMING_ACTIVITY	24	0.85	1.89	0.000	0.000	0.000	4286	UP
		TYPE_I_INTERFERON_RECEPTOR_BINDING	24	0.81	1.79	0.000	0.012	0.022	2482	UP
		TRIPLET_CODON_AMINO_ACID_ADAPTOR_ACTIVITY	22	-0.52	-1.56	0.000	0.011	0.015	12065	DOWN
Zfy1 KO SC1	BP	DISRUPTION_OF_CELL_ANATOMICAL_STRUCTURE_	32	0.84	2.08	0.000	0.000	0.000	3415	UP
	CC	PEPTIDASE_INHIBITOR_COMPLEX	24	0.80	1.90	0.000	0.005	0.005	3623	UP
	MF	PORE_FORMING_ACTIVITY	24	0.92	2.18	0.000	0.000	0.000	2161	UP
		CXCR_CHEMOKINE_RECEPTOR_BINDING	15	0.85	1.90	0.000	0.013	0.026	3162	UP
		FIBRONECTIN_BINDING	36	0.76	1.88	0.000	0.011	0.033	5306	UP
		TRACE_AMINE_RECEPTOR_ACTIVITY	15	-0.81	-2.05	0.000	0.000	0.000	7634	DOWN
		TYPE_I_INTERFERON_RECEPTOR_BINDING	24	-0.64	-1.67	0.000	0.000	0.000	14188	DOWN
		GOMF_PHEROMONE_ACTIVITY	18	-0.53	-1.33	0.000	0.015	0.048	14152	DOWN

All significantly upregulated and downregulated pathways are shown for *Zfy1* KO, *Zfy2* KO, and *Zfy* DKO SC1, SC2, and RS cells. SC1, primary spermatocytes; SC2, secondary spermatocytes; RS, round spermatids; BP, Biological Processes; CC, cellular components' MF, molecular function; Size, total number of genes in each gene set; ES, enrichment score; NES, normalized enrichment score; p-val, nominal p value; q-val, FDR-adjusted p value; FWER pval, family-wise error rate; Rank at Max, the rank of the gene where the ES score has the maximal value; *Expression, UP - gene sets enriched in knock-out, DOWN - gene set enriched in XY.

Table S5. Pathway enrichment analysis (PEA) of *Zfy* DKO germ cell DEGs.

Cell Type	Data -base	Description	Gene Ratio	Bg Ratio	pval	p.adj	qval	Count	-log (padj)	diffexp
Zfy DKO RS	BP	CILIUM_MOVEMENT	52/1603	193/12307	1.50E-07	4.4E-04	4.4E-04	52	3.4	DOWN
		MOTILE_CILIUM_ASSEMBLY	26/1603	69/12307	1.98E-07	4.4E-04	4.4E-04	26	3.4	DOWN
		SPERM_FLAGELLUM_ASSEMBLY	19/1603	46/12307	1.71E-06	2.5E-03	2.5E-03	19	2.6	DOWN
		CILIUM_ORGANIZATION	79/1603	368/12307	3.54E-06	3.9E-03	3.9E-03	79	2.4	DOWN
		SPERM_AXONEME_ASSEMBLY	14/1603	30/12307	7.23E-06	6.4E-03	6.4E-03	14	2.2	DOWN
		CILIUM_OR_FLAGELLUM_DEPENDENT_CELL_MOTILITY	41/1603	164/12307	2.20E-05	1.6E-02	1.6E-02	41	1.8	DOWN
		MICROTUBULE_BASED_MOVEMENT	79/1603	391/12307	3.56E-05	2.3E-02	2.3E-02	79	1.6	DOWN
		SPERM_MOTILITY	36/1603	142/12307	4.97E-05	2.8E-02	2.8E-02	36	1.6	DOWN
		AXONEME_ASSEMBLY	24/1603	81/12307	6.47E-05	3.2E-02	3.2E-02	24	1.5	DOWN
	CC	MANCHETTE	12/1387	21/10533	2.42E-06	1.4E-03	1.3E-03	12	2.9	DOWN
		MOTILE_CILIUM	62/1387	276/10533	1.21E-05	3.5E-03	3.4E-03	62	2.5	DOWN
	MF	HISTONE_BINDING	69/1464	258/11094	3.3E-09	2.9E-06	2.9E-06	69	5.5	UP
		METHYLATION_DEPENDENT_PROTEIN_BINDING	36/1464	101/11094	7.5E-09	3.3E-06	3.3E-06	36	5.5	UP
		MODIFICATION_DEPENDENT_PROTEIN_BINDING	51/1464	204/11094	3.3E-06	9.7E-04	9.6E-04	51	3.0	UP
		SINGLE_STRANDED_DNA_HELICASE_ACTIVITY	12/1464	22/11094	4.8E-06	9.7E-04	9.7E-04	12	3.0	UP
		ATP_DEPENDENT_ACTIVITY_ACTING_ON_DNA	33/1464	113/11094	5.6E-06	9.7E-04	9.7E-04	33	3.0	UP
		HELICASE_ACTIVITY	37/1464	142/11094	2.7E-05	3.5E-03	3.4E-03	37	2.5	UP
		SINGLE_STRANDED_DNA_BINDING	32/1464	116/11094	2.8E-05	3.5E-03	3.4E-03	32	2.5	UP
		UNMETHYLATED_CPG_BINDING	7/1464	10/11094	5.7E-05	6.3E-03	6.3E-03	7	2.2	UP
		MRNA_BINDING	63/1464	308/11094	2.1E-04	2.1E-02	2.1E-02	63	1.7	UP
Zfy DKO SC2	CC	BASEMENT_MEMBRANE	8/99	91/10533	2.0E-06	4.0E-04	3.8E-04	8	3.4	DOWN
		EXTERNAL_ENCAPSULATING_STRUCTURE	14/99	358/10533	5.9E-06	5.7E-04	5.5E-04	14	3.2	DOWN
		COLLAGEN_CONTAINING_EXTRACELLULAR_MATRIX	10/99	282/10533	3.1E-04	2.0E-02	1.9E-02	10	1.7	DOWN

The EnrichR function in ClusterProfiler was used to identify which pathways were significantly upregulated or downregulated in *Zfy* DKO SC1, SC2, and RS cell DEGs (FC >1, FDR <0.05). No significantly de-regulated pathways were identified for *Zfy* DKO SC1 BP, CC, or MF databases, or for *Zfy* DKO SC2 BP or MF databases. SC1, primary spermatocytes; SC2, secondary spermatocytes; RS, round spermatids; Gene ratio, fraction of differentially expressed genes found in gene set; Bg ratio, the ratio of all genes in genes to all genes in database; pval, nominal p value; p.adj, adjusted p value; qval, FDR-adjusted p value; count, gene count; diffexp, up or down differential expression.

Table S6: Gene Ontology (GO) analysis of *Zfy* DKO downregulated DEGs.

	Zfy DKO RS DOWN BP			*Zfy* DKO RS DOWN CC			*Zfy* DKO RS DOWN MF		
**Rank**	**Term ID**	**Term**	**p-val**	**Term ID**	**Term**	**p-val**	**Term ID**	**Term**	**p-val**
1	GO:0051179	localization	7.27E-15	GO:0044424	intracellular part	2.48E-30	GO:0140096	catalytic activity, acting on a protein	6.69E-11
2	GO:0051234	establishment of localization	1.40E-12	GO:0005622	intracellular	2.48E-30	GO:0005515	protein binding	4.14E-09
3	GO:0006996	organelle organization	3.16E-12	GO:0005737	cytoplasm	2.63E-29	GO:0051020	GTPase binding	2.50E-08
4	GO:0033036	macromolecule localization	4.34E-12	GO:0043226	organelle	5.68E-24	GO:0019899	enzyme binding	3.82E-08
5	GO:0051641	cellular localization	1.73E-11	GO:0044464	cell part	4.92E-23	GO:0003824	catalytic activity	7.40E-08
6	GO:0016043	cellular component organization	2.37E-11	GO:0005623	cell	5.96E-23	GO:0043167	ion binding	9.52E-08
7	GO:0006810	Transport	3.59E-11	GO:0043229	intracellular organelle	1.79E-20	GO:0016740	transferase activity	1.52E-07
8	GO:0008104	protein localization	1.59E-10	GO:0044444	cytoplasmic part	4.66E-18	GO:0017016	Ras GTPase binding	1.80E-07
9	GO:0051649	establishment of localization in cell	1.97E-10	GO:0044422	organelle part	9.77E-16	GO:0031267	small GTPase binding	3.48E-07
10	GO:0071840	cellular component organization or biogenesis	9.92E-10	GO:0043227	membrane-bounded organelle	1.37E-14	GO:0043168	anion binding	1.05E-05
11	GO:0036211	protein modification process	4.65E-09	GO:0012505	endomembrane system	4.65E-14	GO:0015631	tubulin binding	1.71E-05
12	GO:0006464	cellular protein modification process	4.65E-09	GO:0044446	intracellular organelle part	8.59E-14	GO:0019787	ubiquitin-like protein transferase activity	2.60E-05
13	GO:0044267	cellular protein metabolic process	5.11E-09	GO:0043231	intracellular membrane-bounded organelle	7.87E-13	GO:0017137	Rab GTPase binding	3.48E-05
14	GO:0070925	organelle assembly	5.86E-09	GO:0042995	cell projection	4.66E-11	GO:0030554	adenyl nucleotide binding	4.61E-05
15	GO:0060271	cilium assembly	1.05E-08	GO:0120025	plasma membrane bounded cell projection	5.20E-11	GO:0035091	phosphatidylinositol binding	5.40E-05
16	GO:0046907	intracellular transport	1.23E-08	GO:0045202	synapse	1.52E-08	GO:0017056	structural constituent of nuclear pore	6.88E-05
17	GO:0048232	male gamete generation	1.40E-08	GO:0015630	microtubule cytoskeleton	2.56E-08	GO:0032559	adenyl ribonucleotide binding	8.96E-05
18	GO:0019538	protein metabolic process	1.86E-08	GO:0005929	cilium	2.70E-08	GO:0016301	kinase activity	8.97E-05
19	GO:1901564	organonitrogen compound metabolic process	2.90E-08	GO:0005783	endoplasmic reticulum	3.09E-08	GO:0004842	ubiquitin-protein transferase activity	1.37E-04
20	GO:0044782	cilium organization	3.34E-08	GO:0044456	synapse part	1.25E-07	GO:1901265	nucleoside phosphate binding	1.38E-04
21	GO:0007276	gamete generation	4.07E-08	GO:0044430	cytoskeletal part	1.58E-07	GO:0000166	nucleotide binding	1.38E-04
22	GO:0043412	macromolecule modification	8.23E-08	GO:0031514	motile cilium	2.20E-07	GO:0005488	binding	2.02E-04
23	GO:0007283	spermatogenesis	8.91E-08	GO:0005829	cytosol	3.74E-07	GO:0042578	phosphoric ester hydrolase activity	2.36E-04
24	GO:0030031	cell projection assembly	1.50E-07	GO:0097223	sperm part	3.75E-07	GO:0036094	small molecule binding	2.70E-04
25	GO:0120031	plasma membrane bounded cell projection	1.80E-07	GO:0032991	protein-containing complex	3.94E-07	GO:0016773	phosphotransferase activity, alcohol group as acceptor	2.90E-04
26	GO:0060285	cilium-dependent cell motility	2.30E-07	GO:0097458	neuron part	4.76E-07	GO:0005524	ATP binding	2.98E-04
27	GO:0001539	cilium or flagellum-dependent cell motility	2.30E-07	GO:0044441	ciliary part	4.97E-07	GO:0004672	protein kinase activity	3.00E-04
28	GO:0007017	microtubule-based process	2.57E-07	GO:0005856	cytoskeleton	5.61E-07	GO:0017076	purine nucleotide binding	3.32E-04
29	GO:0022607	cellular component assembly	2.58E-07	GO:0005815	microtubule organizing center	6.68E-07	GO:0016772	transferase activity, transferring phosphorus-containing	3.40E-04
30	GO:0051179	localization	7.27E-15	GO:0098794	postsynapse	6.69E-07	GO:1901981	phosphatidylinositol phosphate binding	3.74E-04

Top 30 pathways identified by the Gene Ontology analysis function in HOMER. Genes related to spermatogenesis or cell motility are shown in red. BP = biological process, CC = cellular component, MF = molecular function.

Table S7. Gene Ontology (GO) analysis of *Zfy2* KO downregulated DEGs.

Rank	*Zfy2* KO RS DOWN BP			*Zfy2* KO RS DOWN CC			*Zfy2* KO RS DOWN MF		
	Term ID	Term	p-val	Term ID	Term	p-val	Term ID	Term	p-val
1	GO:0006996	organelle organization	1.70E-20	GO:0044424	intracellular part	1.59E-56	GO:0019899	enzyme binding	1.09E-09
2	GO:0046907	intracellular transport	3.02E-15	GO:0005622	intracellular	1.59E-56	GO:0005515	protein binding	8.69E-09
3	GO:0051641	cellular localization	3.58E-15	GO:0043226	organelle	7.05E-48	GO:0016740	transferase activity	3.96E-08
4	GO:0051649	establishment of localization in cell	6.62E-15	GO:0005737	cytoplasm	3.19E-46	GO:0017056	structural constituent of nuclear pore	2.04E-07
5	GO:0044267	cellular protein metabolic process	1.19E-14	GO:0043229	intracellular organelle	9.61E-46	GO:0140096	catalytic activity, acting on a protein	2.19E-07
6	GO:0033036	macromolecule localization	1.01E-13	GO:0043227	membrane-bounded organelle	7.80E-39	GO:0031267	small GTPase binding	4.25E-07
7	GO:0008104	protein localization	1.65E-13	GO:0044446	intracellular organelle part	5.80E-37	GO:0017016	Ras GTPase binding	4.26E-07
8	GO:0045184	establishment of protein localization	3.63E-13	GO:0044422	organelle part	6.40E-36	GO:0051020	GTPase binding	4.44E-07
9	GO:0016043	cellular component organization	4.84E-13	GO:0043231	intracellular membrane-bounded organelle	2.71E-35	GO:0005048	signal sequence binding	1.93E-06
10	GO:0044260	cellular macromolecule metabolic process	9.69E-13	GO:0044464	cell part	3.72E-33	GO:0008139	nuclear localization sequence binding	3.38E-06
11	GO:0044237	cellular metabolic process	2.10E-12	GO:0005623	cell	4.72E-33	GO:0016773	phosphotransferase activity, alcohol group as acceptor	1.22E-05
12	GO:0071840	cellular component organization or biogenesis	3.58E-12	GO:0044444	cytoplasmic part	2.14E-28	GO:0003824	catalytic activity	1.33E-05
13	GO:0036211	protein modification process	9.08E-12	GO:0032991	protein-containing complex	8.62E-21	GO:0008047	enzyme activator activity	1.37E-05
14	GO:0006464	cellular protein modification process	9.08E-12	GO:0005634	nucleus	5.42E-17	GO:0008536	Ran GTPase binding	1.46E-05
15	GO:0043412	macromolecule modification	2.59E-11	GO:0031090	organelle membrane	1.08E-14	GO:0003735	structural constituent of ribosome	1.81E-05
16	GO:0019538	protein metabolic process	4.73E-11	GO:0012505	endomembrane system	1.41E-14	GO:0004672	protein kinase activity	1.88E-05
17	GO:0015031	protein transport	5.63E-11	GO:0044428	nuclear part	9.81E-11	GO:0060589	nucleoside-triphosphatase regulator activity	2.48E-05
18	GO:0015833	peptide transport	2.20E-10	GO:0005654	nucleoplasm	9.93E-11	GO:0030554	adenyl nucleotide binding	2.89E-05
19	GO:0042886	amide transport	3.09E-10	GO:0070013	intracellular organelle lumen	1.09E-10	GO:0005096	GTPase activator activity	3.49E-05
20	GO:0008152	metabolic process	7.67E-10	GO:0043233	organelle lumen	1.14E-10	GO:0030695	GTPase regulator activity	4.38E-05
21	GO:0043170	macromolecule metabolic process	1.07E-09	GO:0031974	membrane-enclosed lumen	1.14E-10	GO:0016772	transferase activity, transferring phosphorus-containing	4.46E-05
22	GO:0051179	localization	1.49E-09	GO:1902494	catalytic complex	3.17E-10	GO:0004445	inositol-polyphosphate 5-phosphatase activity	5.87E-05
23	GO:0007049	cell cycle	1.58E-09	GO:0005829	cytosol	8.37E-10	GO:0030234	enzyme regulator activity	6.24E-05
24	GO:0070727	cellular macromolecule localization	1.69E-09	GO:0031967	organelle envelope	1.43E-09	GO:0032559	adenyl ribonucleotide binding	7.64E-05
25	GO:0034613	cellular protein localization	1.85E-09	GO:0031975	envelope	1.54E-09	GO:0016301	kinase activity	8.42E-05
26	GO:0006807	nitrogen compound metabolic process	1.92E-09	GO:0043228	non-membrane-bounded organelle	1.94E-09	GO:0042578	phosphoric ester hydrolase activity	8.97E-05
27	GO:0070925	organelle assembly	4.81E-09	GO:0031981	nuclear lumen	2.19E-09	GO:0061650	ubiquitin-like protein conjugating enzyme activity	9.91E-05
28	GO:0044238	primary metabolic process	6.77E-09	GO:0043232	intracellular non-membrane-bounded organelle	3.75E-09	GO:0016791	phosphatase activity	1.24E-04
29	GO:0006886	intracellular protein transport	7.04E-09	GO:0015630	microtubule cytoskeleton	8.33E-09	GO:0046030	inositol trisphosphate phosphatase activity	1.34E-04
30	GO:0051234	establishment of localization	1.12E-08	GO:0005789	endoplasmic reticulum membrane	4.02E-08	GO:0017137	Rab GTPase binding	1.62E-04

Top 30 pathways identified by the Gene Ontology analysis function in HOMER. BP = biological process, CC = cellular component, MF = molecular function.

Table S8. Gene Set Enrichment Analysis (GSEA) using the mouse hallmark (mh.all) database.

***Zfy1* KO SC1**

NAME	Size	ES	NES	p-val	q val
ESTROGEN_RESPONSE_LATE	194	0.6	1.8	0.0E+0	3.0E-3
TNFA_SIGNALING_VIA_NFKB	196	0.6	1.8	0.0E+0	2.5E-3
APOPTOSIS	161	0.6	1.7	0.0E+0	3.7E-3
P53_PATHWAY	200	0.6	1.7	0.0E+0	3.5E-3
ADIPOGENESIS	200	0.6	1.7	0.0E+0	4.6E-3
ANGIOGENESIS	36	0.7	1.6	3.0E-3	4.2E-3
HYPOXIA	199	0.6	1.6	0.0E+0	4.7E-3
IL2_STAT5_SIGNALING	199	0.6	1.6	0.0E+0	4.1E-3
PANCREAS_BETA_CELLS	39	0.6	1.6	5.0E-3	4.4E-3
FATTY_ACID_METABOLISM	154	0.6	1.6	0.0E+0	4.1E-3
ESTROGEN_RESPONSE_EARLY	197	0.6	1.6	0.0E+0	3.9E-3
CHOLESTEROL_HOMEOSTASIS	71	0.6	1.6	3.0E-3	4.9E-3
TGF_BETA_SIGNALING	53	0.6	1.6	4.0E-3	5.2E-3
NOTCH_SIGNALING	32	0.7	1.6	5.0E-3	4.9E-3
COAGULATION	134	0.6	1.6	0.0E+0	5.0E-3
EPITHELIAL_MESENCHYMAL_TRAN.	194	0.6	1.6	0.0E+0	4.8E-3
MYOGENESIS	199	0.6	1.6	0.0E+0	4.8E-3
IL6_JAK_STAT3_SIGNALING	85	0.6	1.6	0.0E+0	5.1E-3
HEME_METABOLISM	188	0.6	1.5	0.0E+0	5.1E-3
INTERFERON_GAMMA_RESPONSE	188	0.6	1.5	0.0E+0	4.9E-3
UV_RESPONSE_DN	144	0.6	1.5	0.0E+0	5.7E-3
PROTEIN_SECRETION	94	0.6	1.5	3.0E-3	6.3E-3
MTORC1_SIGNALING	199	0.5	1.5	0.0E+0	6.5E-3
WNT_BETA_CATENIN_SIGNALING	42	0.6	1.5	2.3E-2	1.2E-2
BILE_ACID_METABOLISM	111	0.5	1.5	3.0E-3	1.2E-2

***Zfy2* KO SC1**

NAME	Size	ES	NES	p-val	q-val
TNFA_SIGNALING_VIA_NFKB	196	0.7	1.8	0.0E+0	0.0E+0
FATTY_ACID_METABOLISM	154	0.7	1.8	0.0E+0	0.0E+0
IL2_STAT5_SIGNALING	199	0.6	1.7	0.0E+0	2.0E-3
CHOLESTEROL_HOMEOSTASIS	71	0.6	1.7	0.0E+0	2.3E-3
ANGIOGENESIS	36	0.7	1.7	1.0E-3	2.6E-3
APOPTOSIS	161	0.6	1.6	0.0E+0	2.5E-3
XENOBIOTIC_METABOLISM	196	0.6	1.6	0.0E+0	2.6E-3
PEROXISOME	102	0.6	1.6	0.0E+0	2.3E-3
EPITHELIAL_MESENCHYMAL_TRAN.	194	0.6	1.6	0.0E+0	2.2E-3
MTORC1_SIGNALING	199	0.6	1.6	0.0E+0	2.3E-3
TGF_BETA_SIGNALING	53	0.6	1.6	2.0E-3	2.4E-3
UV_RESPONSE_DN	144	0.6	1.6	0.0E+0	2.3E-3
ADIPOGENESIS	200	0.6	1.6	0.0E+0	2.8E-3
BILE_ACID_METABOLISM	111	0.6	1.6	0.0E+0	3.3E-3
INTERFERON_ALPHA_RESPONSE	94	0.6	1.6	0.0E+0	3.3E-3
P53_PATHWAY	200	0.6	1.6	0.0E+0	3.3E-3
ESTROGEN_RESPONSE_LATE	194	0.6	1.6	0.0E+0	3.2E-3
HYPOXIA	199	0.6	1.6	0.0E+0	3.2E-3
KRAS_SIGNALING_UP	198	0.6	1.6	0.0E+0	3.4E-3
PROTEIN_SECRETION	94	0.6	1.5	1.0E-3	4.6E-3
INTERFERON_GAMMA_RESPONSE	188	0.6	1.5	0.0E+0	5.2E-3
NOTCH_SIGNALING	32	0.6	1.5	1.9E-2	5.8E-3
MYC_TARGETS_V2	58	0.6	1.5	8.0E-3	5.5E-3
ESTROGEN_RESPONSE_EARLY	197	0.5	1.5	0.0E+0	7.9E-3
COAGULATION	134	0.5	1.5	1.0E-3	8.9E-3

***Zfy* DKO SC1**

NAME	Size	ES	NES	p-val	q-val
TNFA_SIGNALING_VIA_NFKB	196	0.7	2.0	0.0E+0	0.0E+0
EPITHELIAL_MESENCHYMAL_TRAN.	194	0.6	1.9	0.0E+0	5.0E-4
TGF_BETA_SIGNALING	53	0.7	1.9	0.0E+0	3.3E-4
FATTY_ACID_METABOLISM	154	0.6	1.8	0.0E+0	5.0E-4
ESTROGEN_RESPONSE_LATE	194	0.6	1.8	0.0E+0	4.0E-4
ADIPOGENESIS	200	0.6	1.8	0.0E+0	1.7E-3
CHOLESTEROL_HOMEOSTASIS	71	0.6	1.8	0.0E+0	1.4E-3
HYPOXIA	199	0.6	1.8	0.0E+0	1.4E-3
XENOBIOTIC_METABOLISM	196	0.6	1.8	0.0E+0	1.2E-3
APOPTOSIS	161	0.6	1.8	0.0E+0	1.1E-3
UV_RESPONSE_DN	144	0.6	1.8	0.0E+0	1.0E-3
PEROXISOME	102	0.6	1.8	0.0E+0	9.2E-4
KRAS_SIGNALING_UP	198	0.6	1.7	0.0E+0	1.5E-3
COAGULATION	134	0.6	1.7	0.0E+0	1.6E-3
NOTCH_SIGNALING	32	0.7	1.7	4.0E-3	1.7E-3
BILE_ACID_METABOLISM	111	0.6	1.7	0.0E+0	1.6E-3
IL2_STAT5_SIGNALING	199	0.6	1.7	0.0E+0	1.7E-3
P53_PATHWAY	200	0.6	1.7	0.0E+0	1.6E-3
OXIDATIVE_PHOSPHORYLATION	195	0.6	1.7	0.0E+0	1.8E-3
IL6_JAK_STAT3_SIGNALING	85	0.6	1.7	0.0E+0	1.8E-3
REACTIVE_OXIGEN_SPECIES_PATHWAY	48	0.6	1.7	1.0E-3	1.9E-3
MYC_TARGETS_V2	58	0.6	1.7	2.0E-3	2.0E-3
ESTROGEN_RESPONSE_EARLY	197	0.6	1.7	0.0E+0	2.0E-3
INTERFERON_ALPHA_RESPONSE	94	0.6	1.7	0.0E+0	2.2E-3
INTERFERON_GAMMA_RESPONSE	188	0.6	1.6	0.0E+0	2.5E-3

***Zfy1* KO SC2**

NAME	Size	ES	NES	p-val	q-val
EPITHELIAL_MESENCHYMAL_TRAN.	194	0.7	1.7	0.0E+0	2.0E-3
TNFA_SIGNALING_VIA_NFKB	196	0.7	1.7	0.0E+0	1.0E-3
INTERFERON_GAMMA_RESPONSE	188	0.7	1.6	0.0E+0	6.7E-4
APOPTOSIS	161	0.7	1.6	0.0E+0	7.5E-4
INTERFERON_ALPHA_RESPONSE	94	0.7	1.6	0.0E+0	6.0E-4
IL2_STAT5_SIGNALING	199	0.6	1.6	0.0E+0	5.0E-4
IL6_JAK_STAT3_SIGNALING	85	0.7	1.6	0.0E+0	4.3E-4
NOTCH_SIGNALING	32	0.7	1.6	2.0E-3	1.0E-3
UV_RESPONSE_DN	144	0.6	1.6	0.0E+0	1.0E-3
KRAS_SIGNALING_UP	198	0.6	1.6	0.0E+0	1.0E-3
INFLAMMATORY_RESPONSE	197	0.6	1.6	0.0E+0	1.2E-3
HYPOXIA	199	0.6	1.5	0.0E+0	1.8E-3
ANGIOGENESIS	36	0.7	1.5	3.0E-3	1.7E-3
COMPLEMENT	185	0.6	1.5	0.0E+0	2.0E-3
TGF_BETA_SIGNALING	53	0.7	1.5	2.0E-3	2.3E-3
P53_PATHWAY	200	0.6	1.5	0.0E+0	3.1E-3
WNT_BETA_CATENIN_SIGNALING	42	0.6	1.5	6.0E-3	3.5E-3
UV_RESPONSE_UP	156	0.6	1.5	0.0E+0	3.4E-3
CHOLESTEROL_HOMEOSTASIS	71	0.6	1.5	2.0E-3	3.5E-3
ALLOGRAFT_REJECTION	192	0.6	1.5	0.0E+0	4.8E-3
ANDROGEN_RESPONSE	96	0.6	1.5	0.0E+0	4.6E-3
COAGULATION	134	0.6	1.5	0.0E+0	4.4E-3
ESTROGEN_RESPONSE_LATE	194	0.6	1.4	0.0E+0	7.0E-3
ESTROGEN_RESPONSE_EARLY	197	0.6	1.4	0.0E+0	7.0E-0
BILE_ACID_METABOLISM	111	0.6	1.4	2.0E-3	8.9E-3

***Zfy2* KO SC2**

NAME	Size	ES	NES	p-val	q-val
TNFA_SIGNALING_VIA_NFKB	196	0.7	1.7	0.0E+0	0.0E+0
EPITHELIAL_MESENCHYMAL_TRAN.	194	0.7	1.7	0.0E+0	0.0E+0
INTERFERON_ALPHA_RESPONSE	94	0.7	1.7	0.0E+0	0.0E+0
INTERFERON_GAMMA_RESPONSE	188	0.7	1.7	0.0E+0	0.0E+0
ANGIOGENESIS	36	0.7	1.7	0.0E+0	0.0E+0
APOPTOSIS	161	0.7	1.6	0.0E+0	0.0E+0
IL6_JAK_STAT3_SIGNALING	85	0.7	1.6	0.0E+0	0.0E+0
INFLAMMATORY_RESPONSE	197	0.7	1.6	0.0E+0	0.0E+0
NOTCH_SIGNALING	32	0.7	1.6	0.0E+0	0.0E+0
KRAS_SIGNALING_UP	198	0.7	1.6	0.0E+0	0.0E+0
HYPOXIA	199	0.7	1.6	0.0E+0	0.0E+0
COAGULATION	134	0.7	1.6	0.0E+0	0.0E+0
IL2_STAT5_SIGNALING	199	0.7	1.6	0.0E+0	0.0E+0
WNT_BETA_CATENIN_SIGNALING	42	0.7	1.6	0.0E+0	0.0E+0
ESTROGEN_RESPONSE_LATE	194	0.6	1.6	0.0E+0	2.7E-4
ALLOGRAFT_REJECTION	192	0.6	1.5	0.0E+0	3.7E-4
COMPLEMENT	185	0.6	1.5	0.0E+0	4.7E-4
UV_RESPONSE_DN	144	0.6	1.5	0.0E+0	4.4E-4
TGF_BETA_SIGNALING	53	0.7	1.5	0.0E+0	5.3E-4
P53_PATHWAY	200	0.6	1.5	0.0E+0	5.0E-4
CHOLESTEROL_HOMEOSTASIS	71	0.6	1.5	0.0E+0	1.8E-3
ESTROGEN_RESPONSE_EARLY	197	0.6	1.5	0.0E+0	1.9E-3
ANDROGEN_RESPONSE	96	0.6	1.4	0.0E+0	2.3E-3
XENOBIOTIC_METABOLISM	196	0.6	1.4	0.0E+0	2.5E-3
UV_RESPONSE_UP	156	0.6	1.4	0.0E+0	3.2E-3

***Zfy* DKO SC2**

NAME	Size	ES	NES	p-val	q-val
TNFA_SIGNALING_VIA_NFKB	196	0.7	1.8	0.0E+0	0.0E+0
INTERFERON_ALPHA_RESPONSE	94	0.7	1.8	0.0E+0	0.0E+0
INTERFERON_GAMMA_RESPONSE	188	0.7	1.8	0.0E+0	0.0E+0
EPITHELIAL_MESENCHYMAL_TRANS.	194	0.6	1.7	0.0E+0	5.0E-4
KRAS_SIGNALING_UP	198	0.6	1.6	0.0E+0	1.4E-3
APOPTOSIS	161	0.6	1.6	0.0E+0	1.7E-3
NOTCH_SIGNALING	32	0.7	1.6	3.0E-3	1.4E-3
COMPLEMENT	185	0.6	1.6	0.0E+0	1.4E-3
INFLAMMATORY_RESPONSE	197	0.6	1.6	0.0E+0	1.6E-3
WNT_BETA_CATENIN_SIGNALING	42	0.7	1.6	2.0E-3	1.7E-3
TGF_BETA_SIGNALING	53	0.6	1.6	4.0E-3	1.8E-3
HYPOXIA	199	0.6	1.6	0.0E+0	1.8E-3
ANGIOGENESIS	36	0.7	1.6	4.0E-3	1.8E-3
IL2_STAT5_SIGNALING	199	0.6	1.6	0.0E+0	1.7E-3
UV_RESPONSE_DN	144	0.6	1.5	0.0E+0	2.7E-3
COAGULATION	134	0.6	1.5	0.0E+0	4.0E-3
ALLOGRAFT_REJECTION	192	0.6	1.5	0.0E+0	3.8E-3
IL6_JAK_STAT3_SIGNALING	85	0.6	1.5	0.0E+0	3.7E-3
CHOLESTEROL_HOMEOSTASIS	71	0.6	1.5	2.0E-3	4.5E-3
UV_RESPONSE_UP	156	0.6	1.5	0.0E+0	6.0E-3
P53_PATHWAY	200	0.6	1.5	0.0E+0	6.2E-3
FATTY_ACID_METABOLISM	154	0.6	1.5	0.0E+0	8.0E-3
MYOGENESIS	199	0.5	1.5	0.0E+0	9.2E-3
ESTROGEN_RESPONSE_LATE	194	0.6	1.5	0.0E+0	8.9E-3
PEROXISOME	102	0.6	1.4	2.0E-3	8.8E-3

The top 25 significant pathways upregulated in *Zfy1* KO, *Zfy2* KO, and *Zfy* DKO SC1/2 cells. No significantly upregulated pathways were identified for *Zfy1* or *Zfy2* KO RS, and only one pathway (TGF_BETA_SIGNALING) was detected for *Zfy* DKO RS. Size, total number of genes in each gene set; ES, enrichment score; NES, normalized enrichment score; p-val, nominal p value; q-val, FDR-adjusted p value.

Table S9. Primer Sequences.

Primer ID	Strand	Primer sequence (5' – 3')	Reference	Target Sequence
Primers for amplifying sex-linked genes upregulated in Zfy DKO whole testis germ cells				
qPCR-*Lrch2*	Forward	TGGTCCCTTTGGCTTGAAGCCT	This study	*Lrch2* (ChrX)
	Reverse	GCTGCCTTATCTGTTCACGCTC		
qPCR-*Ubl4a*	Forward	CGTCACTTCAGTGTAGCAGATGC	This study	*Ubl4a* (ChrX)
	Reverse	CCATAGCCTCAGTCACTTCAGG		
qPCR-*Asb11*	Forward	GCCTTGCTAGAAAACGGTGCAC	This study	*Asb11* (ChrX)
	Reverse	CCAACAGCACATTGACACACGC		
qPCR-*Dcx*	Forward	CTGACTCAGGTAACGACCAAGAC	This study	*Dcx* (ChrX)
	Reverse	TTCCAGGGCTTGTGGGTGTAGA		
qPCR-*Fund2c*	Forward	ATGGTGGCGTAAGCTGTTTGGG	This study	*Fund2c* (ChrX)
	Reverse	TTCCAACCTTCTGGAATACGAAAC		
qPCR-*Magea1*	Forward	GCTCATCTCTGAGGAGTTTGTGC	This study	*Magea1* (ChrX)
	Reverse	CTGGTTTCTGCAAAAGCCCTTGG		
qPCR-*Pgr15l*	Forward	CTGATGGCAGTTGCTATGGACC	This study	*Pgr15l* (ChrX)
	Reverse	ATCGCATGAGGCAGAGCAAGGA		
qPCR-*Xkrx*	Forward	GCATCTAATCCTCTTGGGACCTG	This study	*Xkrx* (ChrX)
	Reverse	CACCTCCCATTCTATCAGCACC		
qPCR-*Atg4a*	Forward	CAGTCTCCACAGCGGATGAGTA	This study	*Atg4a* (ChrX)
	Reverse	GTGTGATGGGTGCTTCTGAACC		
qPCR-*Magea8*	Forward	CCAAGGGCTTTTGCAGAAACCAG	This study	*Magea8* (ChrX)
	Reverse	ATCCACGCCTTGGTCCTGTCTA		
Primers for amplifying genes approaching significant downregulation in Zfy1 KO whole testis germ cells				
qPCR-*Slc16a2*	Forward	GTGTATTCCGCCAGCGCACTTA	This study	*Slc16a2* (ChrX)
	Reverse	AAGAGCACCCAGGTCTCCTTGA		
qPCR-*Ids*	Forward	CTTTCTTCCTGGCAGTTGGGTAC	This study	*Ids* (ChrX)
	Reverse	GGTTGTAGGCTACAGGTGGTAG		
qPCR-*Nhs*	Forward	AGACCCAAGGAAGTATGGACGAG	This study	*Nhs* (ChrX)
	Reverse	CTCCAGCTTGAGAGTCCTCACT		
qPCR-*Hdac6*	Forward	TCGCTGTCTCATCCTACCTGCT	This study	*Hdac6* (ChrX)
	Reverse	GTCAAAGTTGGCACCTTCACGG		
qPCR-*Acsl4*	Forward	CCTTTGGCTCATGTGCTGGAAC	This study	*Acsl4* (ChrX)
	Reverse	GCCATAAGTGTGGGTTTCAGTAC		
qPCR-*Tspyl2*	Forward	TTTGGAGCGCAGAGACCTCATC	This study	*Tspyl2* (ChrX)
	Reverse	CCTGAAGATTGGTCAAGTAGCGG		
qPCR-*Tsr2*	Forward	CAGTGGAGGATTACTTCATCGCC	This study	*Tsr2* (ChrX)
	Reverse	GCAGACTCCCATCTTCCACAAC		
qPCR-*Diaph2*	Forward	TCAGTACAAAGTCATTCAGTGCC	This study	*Diaph2* (ChrX)
	Reverse	CATATGGAGAGGTGACAAGTGC		
Primers for amplifying ubiquitously expressed housekeeping genes				
qPCR-*Ppia*	Forward	TTACCCATCAAACCATTCCTTCTG	(1)	*Ppia* (Chr11)
	Reverse	AACCCAAAGAACTTCAGTGAGAGC		
qPCR-*Rsp18*	Forward	CGGAAAATAGCCTTCGCCATCAC	This Study	*Rsp18* (Chr17)
	Reverse	ATCACTCGCTCCACCTCATCCT		
qPCR-*Rplp0*	Forward	CAAAGCTGAAGCAAAGGAAGAG	(1)	*Rplp0* (Chr5)
	Reverse	AATTAAGCAGGCTGACTTGGTTG		
Primers for amplifying spermiogenesis genes downregulated in Zfy DKO RS				
qPCR-Aurka	Forward	TCATCCTGGCTCTGAAGGTGCT	This study	*Aurka (Chr2)*
	Reverse	CCATACAGCCTGAGGATGTTGG		

qPCR-Cep131	Forward	GTTCTGAGCGAGAAGTGTGAGG	This study	*Cep131 (Chr11)*
	Reverse	GGATTTTCTCGGTGGCACTCATG		
qPCR-Ccdc39	Forward	CTGAGAAGGAACTGAGCAAAGCC	This study	*Ccdc39 (Chr3)*
	Reverse	AGGACTTCCTCTGCCTTGCTGT		
qPCR-Parva	Forward	GGAATGAACGCCATCAACCTGC	This study	*Parva (Chr7)*
	Reverse	CATTGCGTGAGTTTGGATCGACC		
qPCR-Tmem231	Forward	TCCTTTTGCCCAGGACTACGAC	This study	*Tmem231 (Chr8)*
	Reverse	TGACAGCATGGATCACGAACGG		
qPCR-Snap29	Forward	TAGAGCCTCCACCTGAGCAGAA	This study	*Snap29 (Chr16)*
	Reverse	GCATCCTGTAGCCTTCTGAGGT		
qPCR-Slc9c1	Forward	GACCCTCAACATTGAAGCCATGG	This study	*Slc9c1 (Chr16)*
	Reverse	GCACTCTGGGACAGAACCTCAT		
qPCR-Celsr2	Forward	CATGAAGGACCTCCAGGTGGAT	This study	*Celsr2 (Chr3)*
	Reverse	CGTTGTGGCAAATGCTGCTGTC		
qPCR-Mapk15	Forward	TCCAGGACCTTGGCTCAGACTA	This study	*Mapk15 (Chr15)*
	Reverse	AGCAAACGCCAAGAGTCGCTTG		
qPCR-Ablim3	Forward	CTCGTTCACACTACCTGGCTGA	This study	*Ablim3 (Chr18)*
	Reverse	TGCTGTCGTAGTGGAAGAGGTC		
Primers for amplifying alternatively spliced genes deregulated in Zfy DKO RS				
qPCR-Gm1	Forward	GAGGTCTGGTTCGATGAGAAGG	This study	*Gm1 (Chr10)*
	Reverse	TTCAGCACACCTTCATGCCAGG		
qPCR-Pp2r3d	Forward	AGGCTGTGTCACAGTTGGAGGT	This study	*Pp2r3d (Chr9)*
	Reverse	AGGTCGCTCCTGTCAATGGTGA		
qPCR-Casc4	Forward	GCTGAGCTTCGTCAGGAGTTTC	This study	*Casc4 (Chr2)*
	Reverse	CCTTGATCTGCTGTCCACACTG		
qPCR-Gnb1l	Forward	GTAATCTGGCTGAAGACACTGCC	This study	*Gnb1l (Chr16)*
	Reverse	CTGAGTCCATGATGGTGTTCCTG		
qPCR-Mfsd11	Forward	GTCGCCATTGTTGGACCTCAGA	This study	*Mfsd11 (Chr11)*
	Reverse	GACAGATGCTGTGTAGAAGGACC		
qPCR-Tubd1	Forward	CAGCCTACATACTCTGAGGACAG	This study	*Tubd1 (Chr11)*
	Reverse	TAAACGCGCTGTACGCCAGAGA		
qPCR-Josd2	Forward	GATGTCAACGTGATCATGGCTGC	This study	*Josd2 (Chr7)*
	Reverse	GACACAGGAGAGGGTAGGTTCA		
qPCR-Cfl2	Forward	GCTCCTGAAAGTGCACCGTTAAA	This study	*Cfl2 (Chr12)*
	Reverse	GCGGTCCTTAATATCGTCCAAGC		
qPCR-Ccdc88c	Forward	CATGGAGCTACTCCGTGTGAAG	This study	*Ccdc88c (Chr12)*
	Reverse	CGTTCTGAGTCTCCAAGTGCTG		
qPCR-Piga	Forward	CTTTCTACACCTGGCGGAACGT	This Study	*Piga (ChrX)*
	Reverse	GCCACAGTGTGAGATGAGCCTG		
qPCR-Unk	Forward	GCTATGCCTGTCCCTACTACCA	This study	*Unk (Chr11)*
	Reverse	CCATGCTTGACGTTTGGACACG		
Primers for amplifying apoptosis genes upregulated in Zfy DKO SC1/SC2				
qPCR-Bcl2l2	Forward	CAAGTGCAGGATTGGATGGTGG	This study	*Bcl2l2 (Chr 14)*
	Reverse	CTGTCCTCACTGATGCCCAGTT		
qPCR-Gadd45b	Forward	GGAGACATTGGGCACAACCGAA	This study	*Gadd45b (Chr10)*
	Reverse	CTGCTCTCTTCACAGTAACTGGC		
qPCR-Btg2	Forward	GAGCGAGCAGAGACTCAAGGTT	This study	*Btg2 (Chr1)*
	Reverse	CGATAGCCAGAACCTTTGGATGG		

qPCR-Gsr	Forward	GTTTACCGCTCCACACATCCTG	This study	*Gsr (Chr8)*
	Reverse	GCTGAAAGAAGCCATCACTGGTG		
qPCR-Bgn	Forward	TGAACCAGGAGCCTTTGATGGC	This study	*Bgn (ChrX)*
	Reverse	GTCCTCCAACTCAATAGCCTGG		
qPCR-Gch1	Forward	AGCAAGTCCTTGGTCTCAGTAAAC	This study	*Gch1 (Chr14)*
	Reverse	ACCGCAATCTGTTTGGTGAGGC		
Primers for amplifying sex-linked genes upregulated in Zfy DKO RS				
qPCR-Magea5	Forward	GCTCATCTCTGAGGAGTTTGTGC	This study	*Magea5 (ChrX)*
	Reverse	CTGGTTTCTGCAAAAGCCCTTGG		
qPCR-Asb11	Forward	GCCTTGCTAGAAAACGGTGCAC	This Study	*Asb11 (ChrX)*
	Reverse	CCAACAGCACATTGACACACGC		
qPCR-Mpp1	Forward	CTACAGAAGCGTAACCGACCTG	This Study	*Mpp1 (ChrX)*
	Reverse	ATGAGACGAACTCTCCGAGCCT		
qPCR-Map7d2	Forward	TGGAGGAAAGGCTGAGCACAGT	This Study	*Map7d2 (ChrX)*
	Reverse	CCTCTTGTTCCTTCTGTAGCCG		
qPCR-Msn	Forward	GAAAGTGAGGCTGTGGAATGGC	This Study	*Msn (ChrX)*
	Reverse	CTCTGCTCCATTCTCATCCTGC		
qPCR-Hmgb3	Forward	AGAAGTGCTCGGAGAGGTGGAA	This Study	*Hmgb3 (ChrX)*
	Reverse	TCTTGCCTCCTTTAGCTGGTCC		
qPCR-Atrx	Forward	GTGAATCTGAGGATGAACAGCGG	This Study	*Atrx (ChrX)*
	Reverse	CCTCTTGAACCTTAATGCGCCG		
Primers for amplifying chromatin genes upregulated in Zfy DKO RS				
qPCR-Tbl1	Forward	CCGAGTAAAGCCACAGTCCTTC	This study	*Tbl1 (ChrX)*
	Reverse	TATCCTCGCAGTGGAGTCTCCA		
qPCR-Rbbp7	Forward	CTCAGCTGTTGTAGAGGATGTGG	This study	*Rbbp7 (ChrX)*
	Reverse	CCACCAAATGGCTCGGCTTAGA		
qPCR-Noc2l	Forward	CCTTCCTCTACATCCGACAGCT	This study	*Noc2l (Chr4)*
	Reverse	GCAGGATCTCACTGGAACCAAG		
qPCR-Anp32b	Forward	GCAGGATCTCACTGGAACCAAG	This study	*Anp32b (Chr4)*
	Reverse	CTCTCGGTCATAGCCATCCAGA		
qPCR-Taf1	Forward	ATCCTACAGGCTGTGGTGAAGG	This study	*Taf1 (ChrX)*
	Reverse	TTCAGAGACAGGCGACGAAGGT		
Primers for amplifying spermiogenesis genes downregulated in Zfy DKO RS				
Piga NS-2	Forward	GAGAGAGGGCACAAGGTCAT	This study	*Piga (ChrX) exon 2, coding sequence (non-spliced region) amplicon size 150 bp*
	Reverse	TGGCAGACTGTGAAAGAGAGT		
Piga NS-3	Forward	CCTCCTTCAGTGTCGCCTT	This study	*Piga (ChrX) exon 2, coding sequence (non-spliced region) amplicon size 170 bp*
	Reverse	ATGACCTTGTGCCCTCTCTC		
Piga-3	Forward	CGGGAGAGAATTACGATAATCCA	This study	*Piga (ChrX) exon 2, coding sequence (spliced region) amplicon size 167 bp*
	Reverse	GACACAGTTAGAAGCTTGTTTGT		
Piga-4	Forward	AGACAATGGGGCTTCAGACA	This study	*Piga (ChrX) exon 2, coding sequence (spliced region) amplicon size 230 bp*
	Reverse	TGGGTCTGGAGTGAAGTCAG		

Table S10. Antibodies

Target	Manufacturer	Reference	Usage
Western Blot primary antibodies			
PRM2	Briar Patch Biosciences	#Mab-Hub2B	1:1000
Western Blot secondary antibodies			
Goat anti-mouse HRP	ThermoFisher	#31430	1:5000
Immunofluorescence antibodies			
Mouse Anti-TNP2	Santa Cruz	SC-393843	1:100
Rabbit Anti-acetyl-Histone H4	Sigma-Aldrich	06-866	1:500
Anti-rabbit IgG (H+L), F(ab')$_2$ Fragment (Alexa Fluor® 488 Conjugate)	Cell Signaling	4412	1:500
Anti-mouse IgG (H+L), F(ab')$_2$ Fragment (Alexa Fluor® 647 Conjugate)	Cell Signaling	4410	1:500

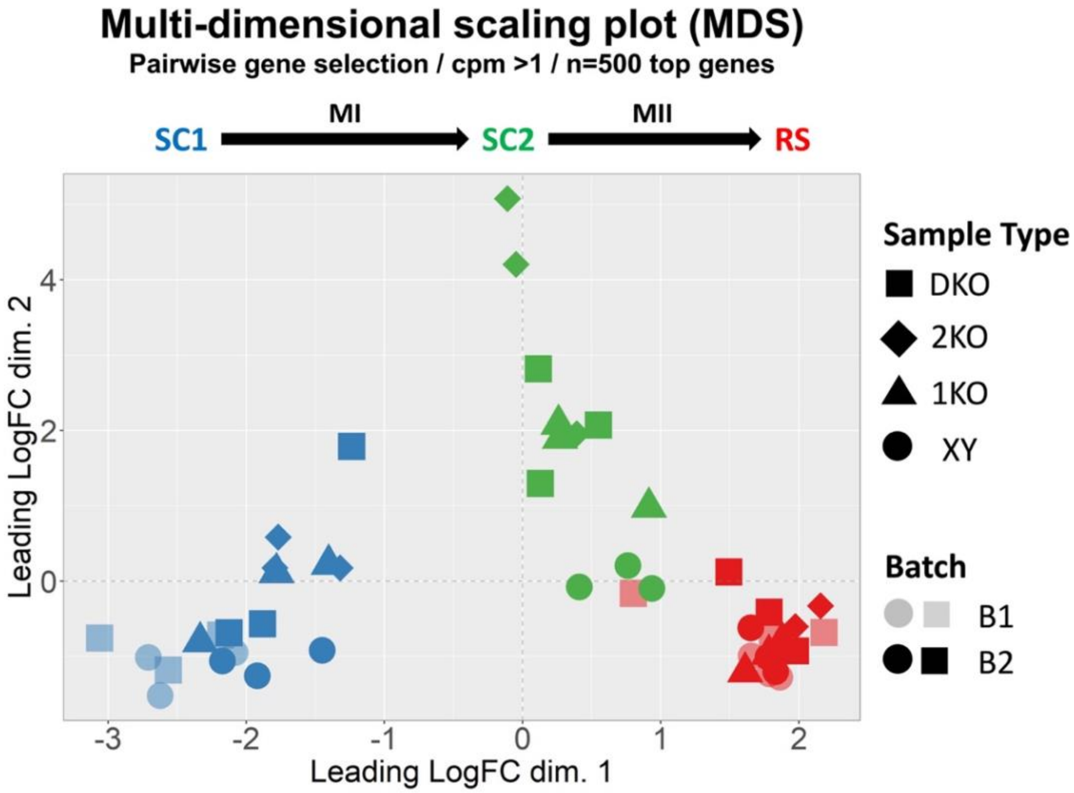

Figure S1: Comparison of transcriptomes for pilot and expanded experiment. Multiple dimension scaling (MDS) plot showing the transcriptional similarities between the pilot experiment (B1, light shade) and expanded experiment (B2, dark shade). Only XY and *Zfy* DKO SC1 and RS were included in B1, whereas all cell types and genotypes were included in B2. Because the transcriptomes of XY and *Zfy* DKO SC1 and RS were similar in pilot and expanded experiment, the data from both experiments were analyzed together. SC1 = primary spermatocytes, SC2 = secondary spermatocyte, RS = round spermatid, DKO = *Zfy* DKO, 1KO = *Zfy1* KO, 2KO = *Zfy2* KO, XY = WT.

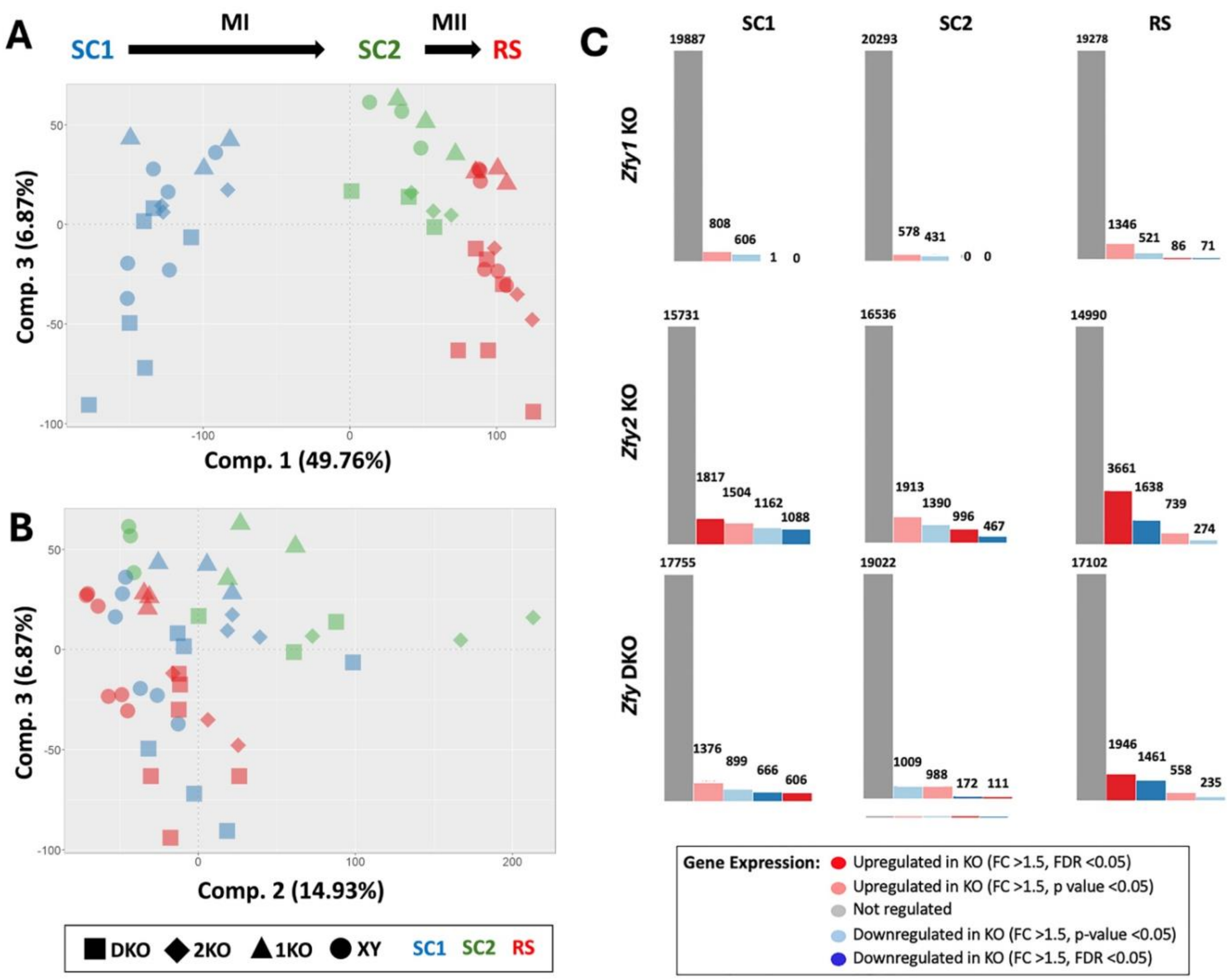

Figure S2: Significantly deregulated genes in *Zfy* KO germ cells. A-B: Principal component analysis (PCA) of component 1 vs component 3 (**A**) and component 2 vs component 3 (**B**) comparing transcriptomes of XY, *Zfy1* KO, *Zfy2* KO, and *Zfy* DKO primary spermatocytes (SC1), secondary spermatocytes (SC2), and round spermatid (RS). MI = meiosis I, MII = meiosis II. **C**: Bar graphs illustrating the number of significantly de-regulated genes and genes approaching significant de-regulation for *Zfy1* KO, *Zfy2* KO, and *Zfy* DKO SC1, SC2, and RS cells. Significantly upregulated = red, approaching significant upregulation = light red, not regulated = gray, approaching significant downregulation = light blue, and significantly downregulated = dark blue.

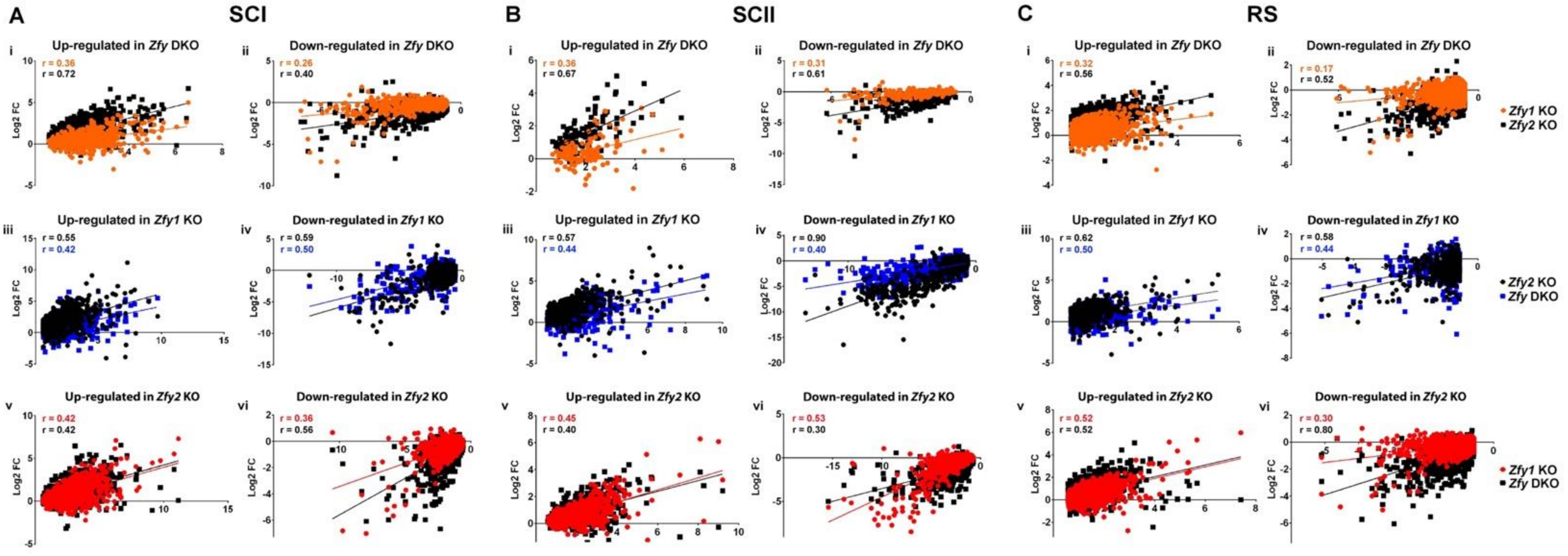

Figure S3: Correlation of differentially expressed genes (DEGs) between *Zfy* KO models. A-C: Scatter plots of differentially expressed genes (DEGs) for *Zfy1* KO (fold change >1.5, p-value <0.05), *Zfy2* KO (fold change >1.5, FDR <0.05), and *Zfy* DKO (fold change >1.5, FDR <0.05) primary spermatocytes (SC1), secondary spermatocytes (SC2) and round spermatid (RS) cells. For *Zfy1* KO p-value was used because there were not enough DEGs for FDR. The Log_2 fold change (FC) of the main genotype is shown on the X axis, and the Log_2 FC of the tested genotypes (with r values) is shown on the Y axis.

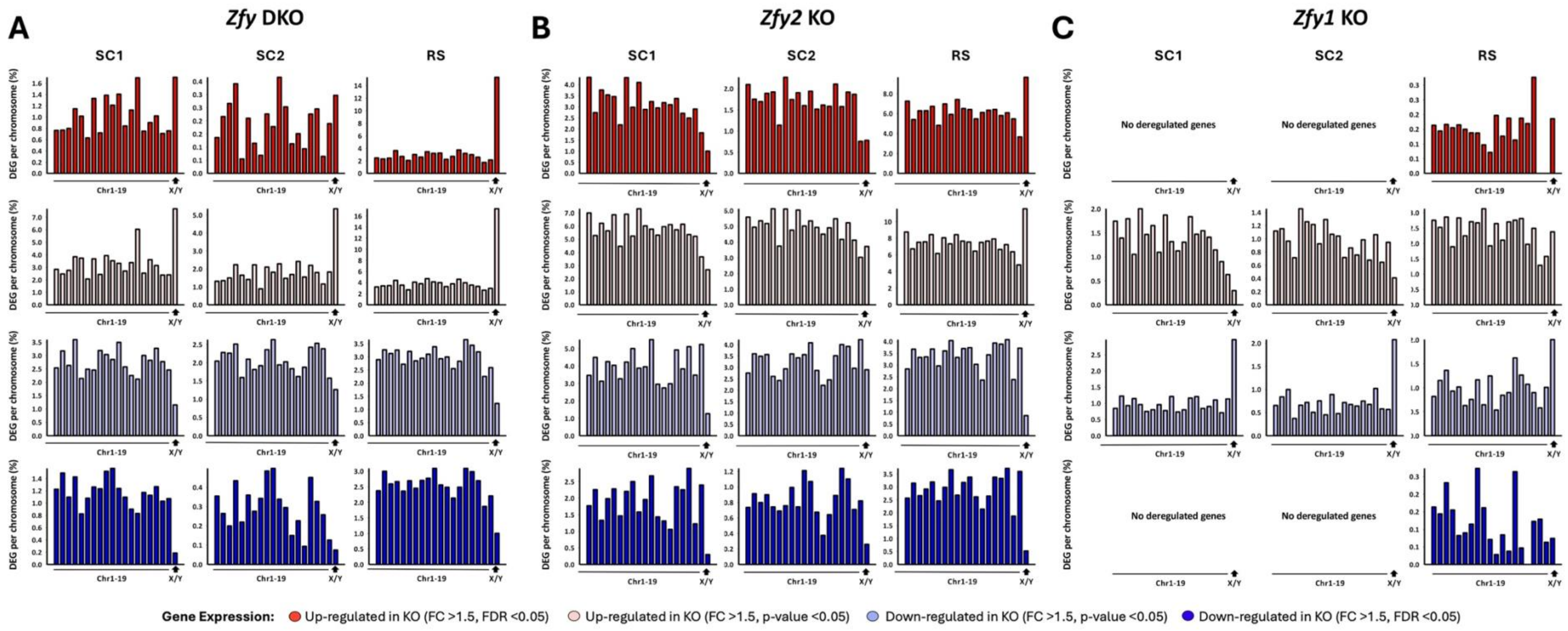

Figure S4: Differentially expressed genes (DEGs) by chromosome. Differentially expressed genes (DEGs, FC >1.5, FDR <0.05) and genes approaching significant deregulation (FC >1.5, p val <0.05) listed by chromosome for *Zfy* DKO (**A**), *Zfy2* KO (**B**), and *Zfy1* KO (**C**) primary spermatocytes (SC1), secondary spermatocytes (SC2) and round spermatids (RS). DEGs are shown in dark red and dark blue, genes approaching significant de-regulation are shown in light red and light blue. Values are shown as percent of DEGs per chromosome, with total gene number for each chromosome taken from the Mouse Genome Informatics (MGI) database. Chr1-19 = mouse autosomal chromosomes 1-19, X/Y = sex chromosomes.

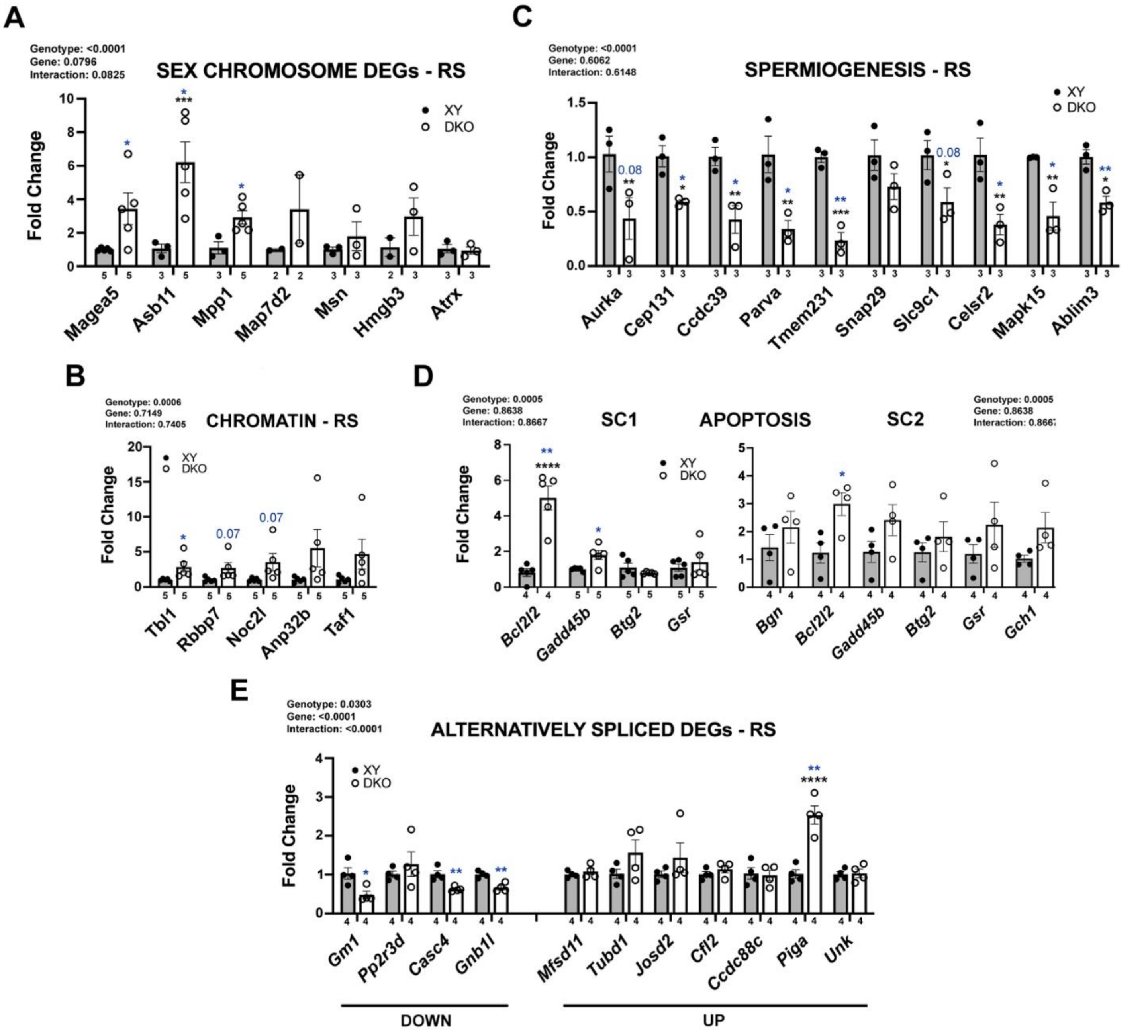

Figure S5. qPCR validation of gene deregulation detected by RNA-seq. Deregulation of genes detected by RNA-seq was validated by RT-qPCR with purified primary spermatocytes (SC1), secondary spermatocytes (SC2), and round spermatids (RS) from *Zfy* DKO (DKO, empty circles) and wild-type control (XY, filled circles), with the geometric mean of *Ppia*, *Rsp18*, and *Rplp0* used as a reference. Differentially regulated genes (DEGs) from Sex Chromosome (A), Chromatin (B), Spermiogenesis (C), Apoptosis (D) pathways and DEGs that were included among alternatively spliced genes (E) groups were tested. In E, DOWN and UP are genes that were downregulated and upregulated, respectively, in the RNA-seq analysis. Graphs are averages with SEM (B,C,D,E) and with SDev (A), with number of males indicated under each bar and individual data points shown as empty (DKO) or filled (XY) circles. The data were analyzed with 2-way ANOVA with Genotype and Gene as factors (P value shown within each graph) and post-hoc Holm-Šidak test for paired XY vs. DKO comparison (*, P<0.05; **, P<0.01; ***, P<0.001; ****, P<0.0001). The paired comparisons were also analyzed with standard t-test (two-tailed, homoscedastic) and respective P values are shown as blue color asterisk, to distinguish from black asterisk reflecting Holm-Šidak test.

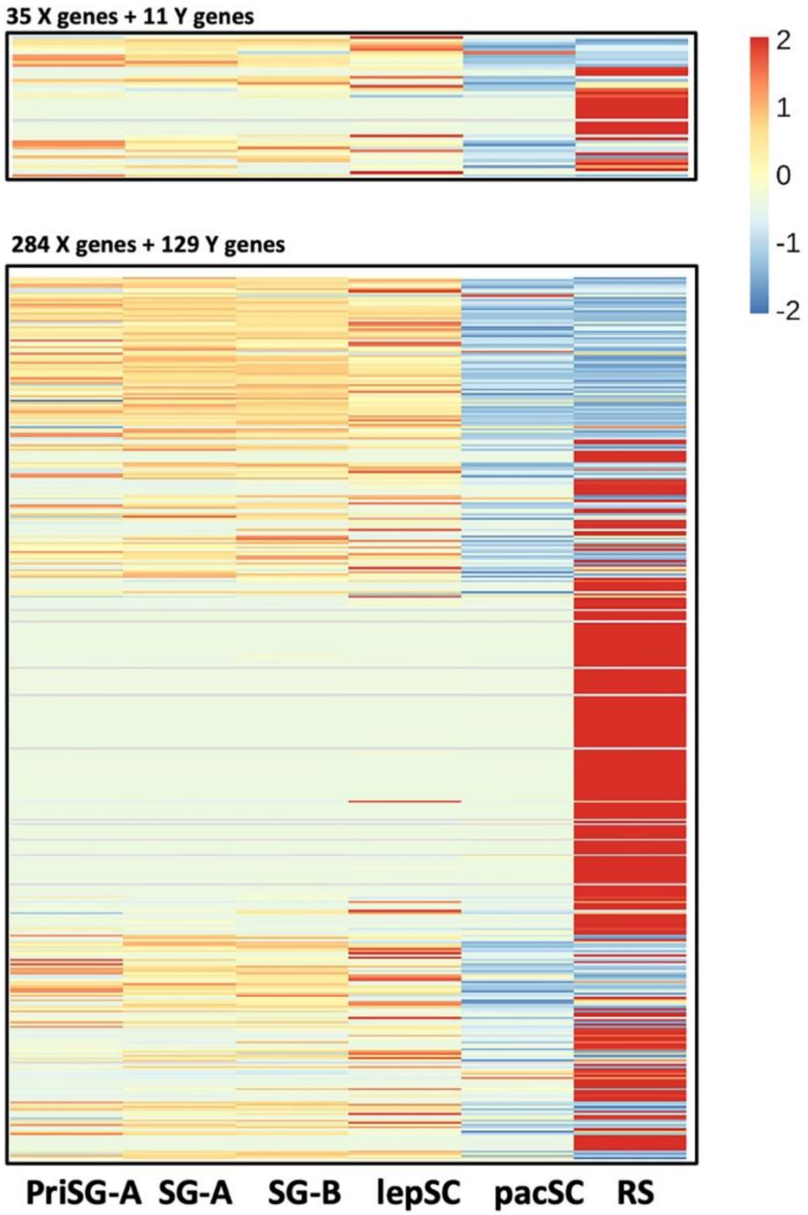

Figure S6. Expression dynamics of X/Y-linked genes. Expression dynamic of X/Y-linked genes found upregulated in *Zfy* DKO SC1 (top panel) and in *Zfy* DKO RS (bottom panel) during wild-type spermatogenesis. Warm colors indicate high expression levels, cold colors low expression levels/no expression. Data are from GSE35005 analyzed in Moretti et al 2016 (2). PriSG-A: primitive type A spermatogonia; SG-A and SG-B: Type A or Type B spermatogonia; lepSC: preleptotene spermatocytes; pacSC: pachytene spermatocytes; RS: round spermatids.

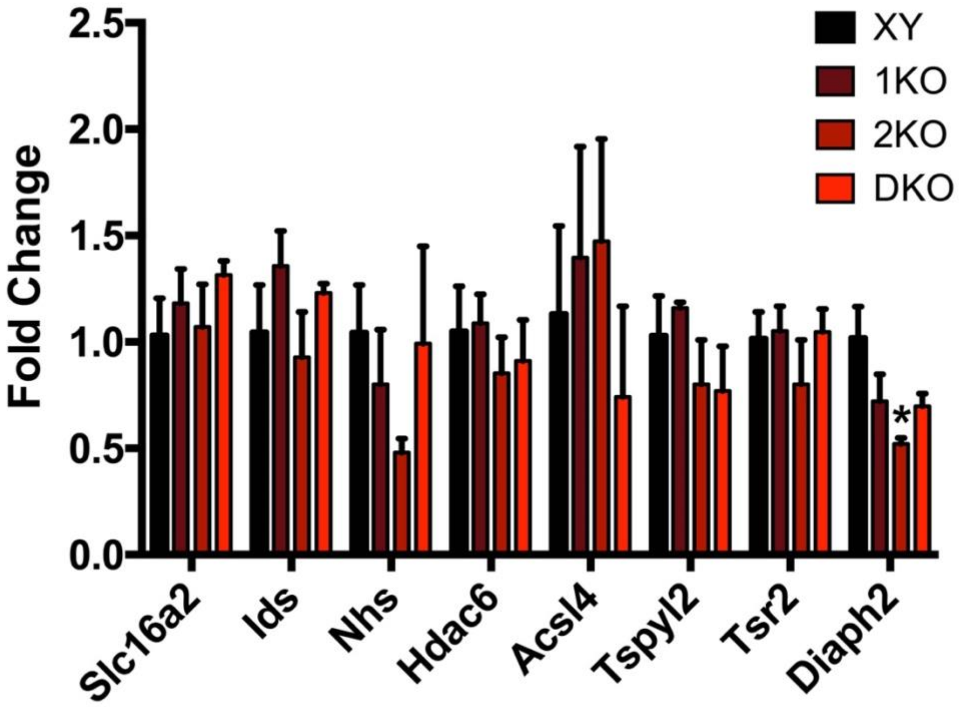

Figure S7: Expression of down-regulated sex-linked genes in *Zfy1* KO germ cells. qPCR of sex-linked genes in XY (black) *Zfy1* KO (dark red), *Zfy2* KO (red), and *Zfy* DKO (light red) whole testes, with the geometric mean of *Ppia*, *Rsp18*, and *Rplp0* used as a reference. Graphs are n=3 ± SEM. Statistical significance (unpaired t-test): * $P<0.05$.

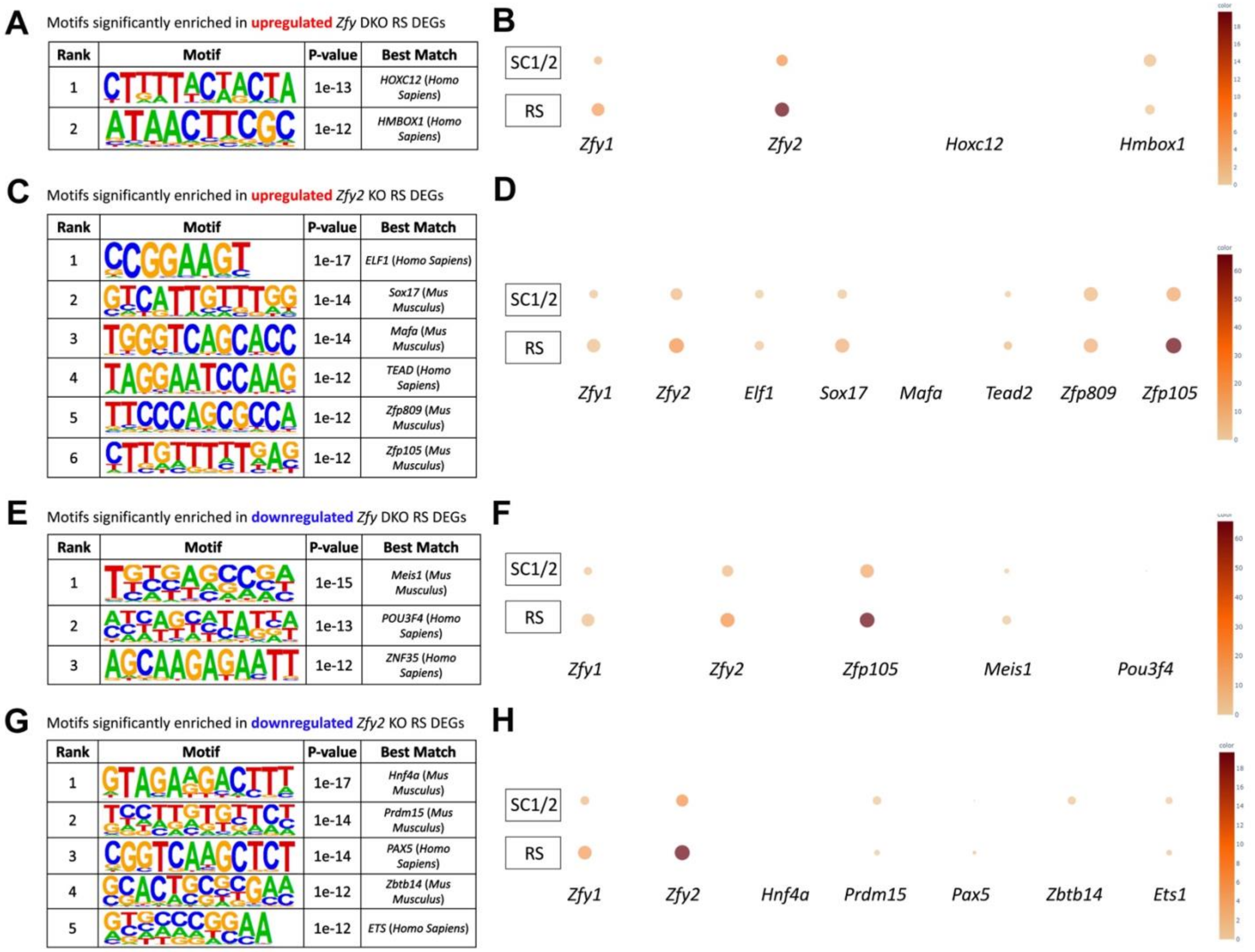

Figure S8: Identifying potential binding partners for ZFY in RS. A: HOMER results showing all significantly enriched motifs among *Zfy* DKO upregulated DEGs (FC >2.0, FDR <0.05). The "Best Match" column refers to the best human/mouse gene match for each motif. **B**: Previously reported (3) single cell RNA sequencing (scRNA-seq) data for the mouse orthologs of the top gene matches for significantly enriched motifs in *Zfy* DKO RS upregulated DEGs. Expression levels were assessed in relation to *Zfy1* and *Zfy2* in the male germline. High gene expression is shown in darker shade, low gene expression in light shade. SC1/2 = primary + secondary spermatocytes, RS = round spermatids. **C**: All significant motif enrichment results for *Zfy2* KO upregulated DEGs. **D**: Previously reported (3) RNA-seq data for "Best Match" HOMER results for *Zfy2* KO upregulated DEGs. Expression of the mouse orthologs of each best match is shown in relation to *Zfy1* and *Zfy2*. **E**: All significant motif enrichment results for *Zfy* DKO downregulated DEGs. F: Previously reported (Chen et al., 2018) RNA-seq data for "Best Match" HOMER results for *Zfy* DKO downregulated DEGs. Expression of the mouse orthologs of each best match is shown in relation to *Zfy1* and *Zfy2*. **G**: All significant motif enrichment results for *Zfy2* KO downregulated DEGs. **H**: Previously reported (3) RNA-seq data for "Best Match" HOMER results for *Zfy2* KO downregulated DEGs. Expression of the mouse orthologs of each best match is shown in relation to *Zfy1* and *Zfy2*.

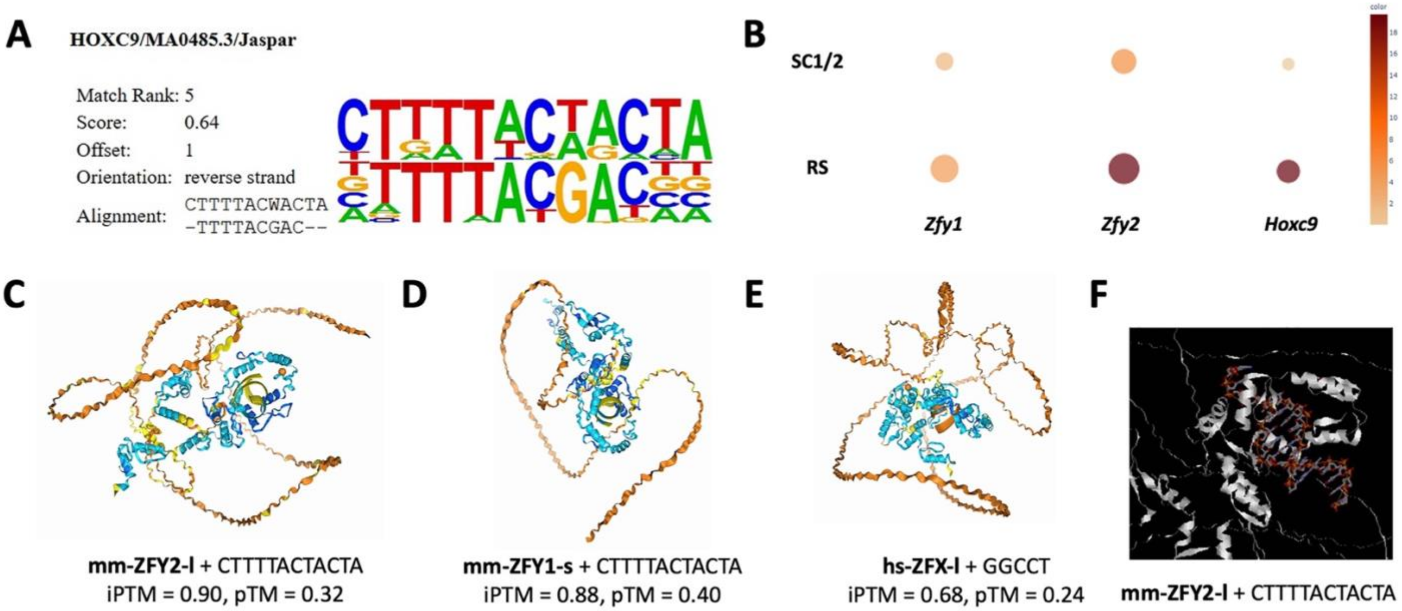

Figure S9. Alphafold modeling of potential ZFY and ZFX binding motifs identified by MEA. A: HOMER results showing match data for HOXC9 to the top ranked motif enriched in upregulated *Zfy* DKO RS DEGs. **B:** Previously reported (3)single cell RNA sequencing (scRNA-seq) data for *Hoxc9*. Expression levels were assessed in relation to *Zfy1* and *Zfy2* in the male germline. High gene expression is shown in darker shade, low gene expression in light shade. SC1/2, primary + secondary spermatocytes; RS, round spermatids. **C-E:** Alphafold modeling results for **(C)** the long isoform of mouse ZFY2 (mm-ZFY2-l) binding to the most significantly enriched motif in upregulated *Zfy* DKO RS DEGs, **(D)** the short isoform of mouse ZFY1 (mm-ZFY1-s) binding to the most strongly enriched motif in upregulated *Zfy* DKO RS DEGs, and **(E)** the long isoform of ZFX (hs-ZFX-l) binding to a previously reported ZFX-family motif. Dark blue = high significance, light blue = confident significance, yellow = low significance, orange = very low significance. iPTM = interface-predicted template modeling score; pTM = predicted template modeling score. **F:** Modeling of mm-ZFY2-l protein sequence monomer binding to the motif DNA helix in presence of a zinc ion.

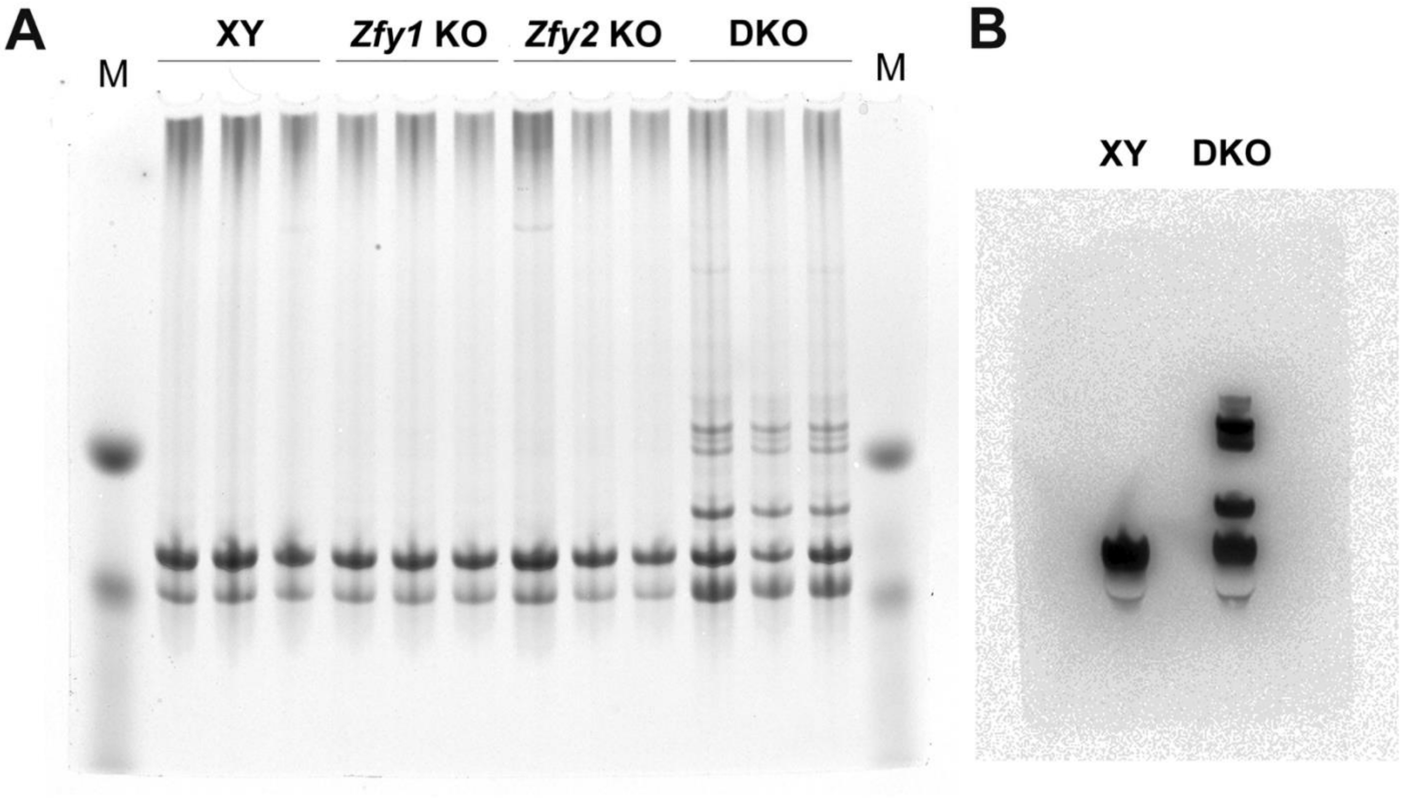

Figure S10. Protamine content in sperm from *Zfy* KO males – untrimmed gel and blot. A: Coomassie stained photo of untrimmed acid urea polyacrylamide gel electrophoresis (AU-PAGE) of cauda epididymal sperm basic nuclear protein extracts from XY, *Zfy1* KO, *Zfy2* KO and *Zfy* DKO males; this panel relates to Fig. 4Ai. **B**: Untrimmed photo of acidic Western blot using anti-PRM2 antibody of cauda epididymal sperm basic nuclear protein extracts from XY and *Zfy* DKO males; this panel relates to Fig. 4Aii.

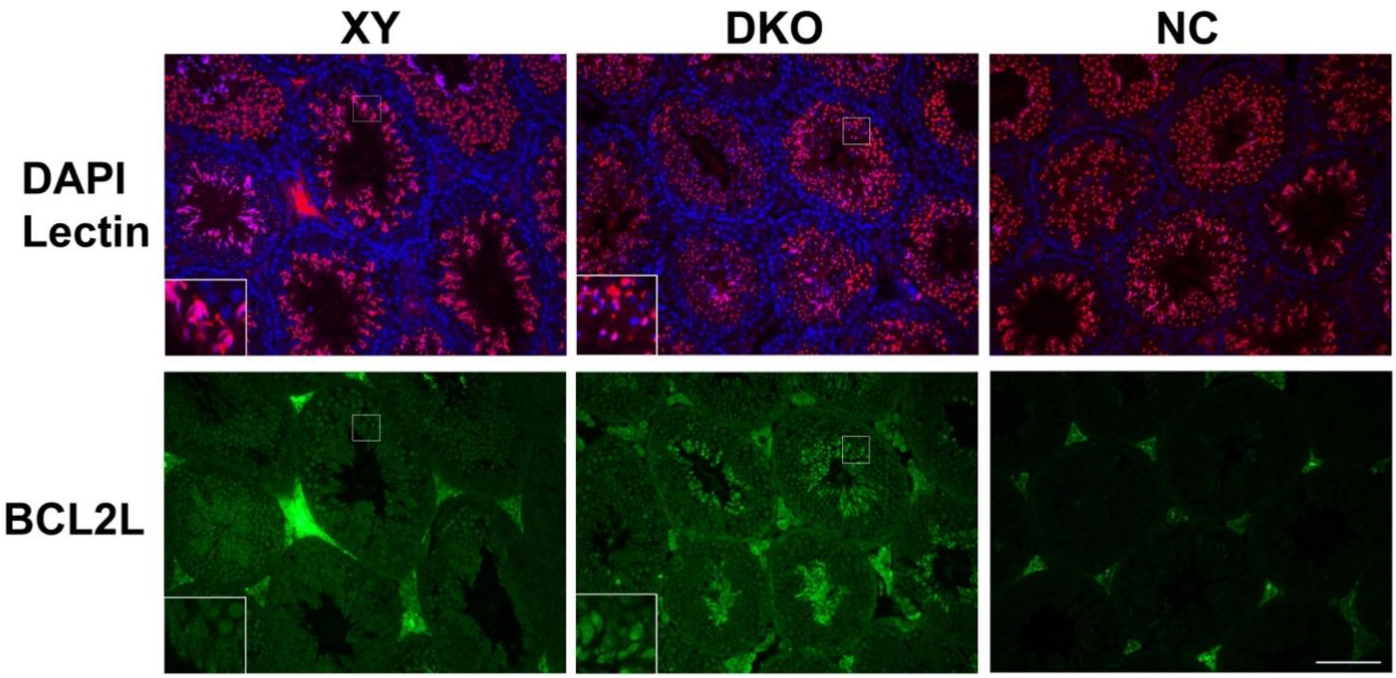

Figure S11. Immunofluorescence detection of BCL2L. Immunofluorescence detection of apoptosis regulator BCL2L (green) in testis sections from wild-type (XY) and *Zfy* DKO (DKO) males, with negative control (NC) missing anti-BCL2L antibody. Hoechst (blue) was used to stain nuclei and Lectin (red) was used to stain acrosome. In XY, BCL2L no cell-specific signals are seen while in DKO many germ cells fluoresce green. These cells are elongating/elongated spermatids accumulating near tubule lumens; these spermatids are morphologically abnormal as always seen with this genotype. Insets, 3x magnification. Scale, 100 µm.

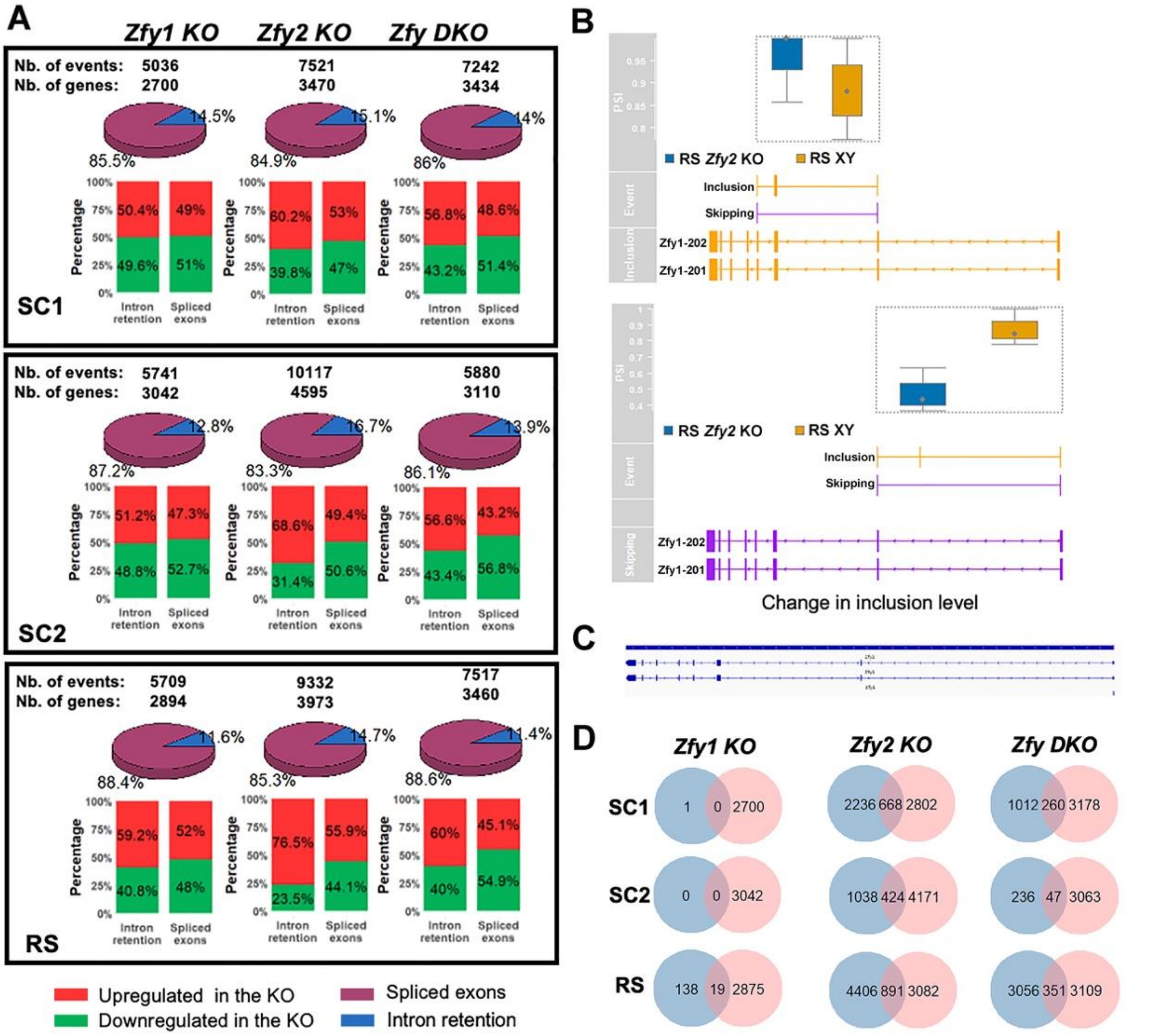

Figure S12. *Zfy* loss deregulates the splicing of hundreds of transcripts. A: Pie chart and bar graph representing the distribution of differential splicing events (with a threshold of deltaPSI = 10% and FDR <0.05), identified by rMATS alone. **B:** Transcripts showing a change in inclusion levels (PSI) of exon 4 (upper panel) and exon 2 (lower panel) of *Zfy1* due to *Zfy2* knockout in RS. **C:** Annotation of *Zfy1* showing a tiny exon 2, from ENSEMBL gtf file (GRCm38, release102). **D:** Venn diagrams illustrating the overlap between differentially expressed genes (DEGs, with FC = 1.5, FDR <0.05) and splicing-regulated genes as per rMATS (deltaPSI = 10%, FDR <0.05).

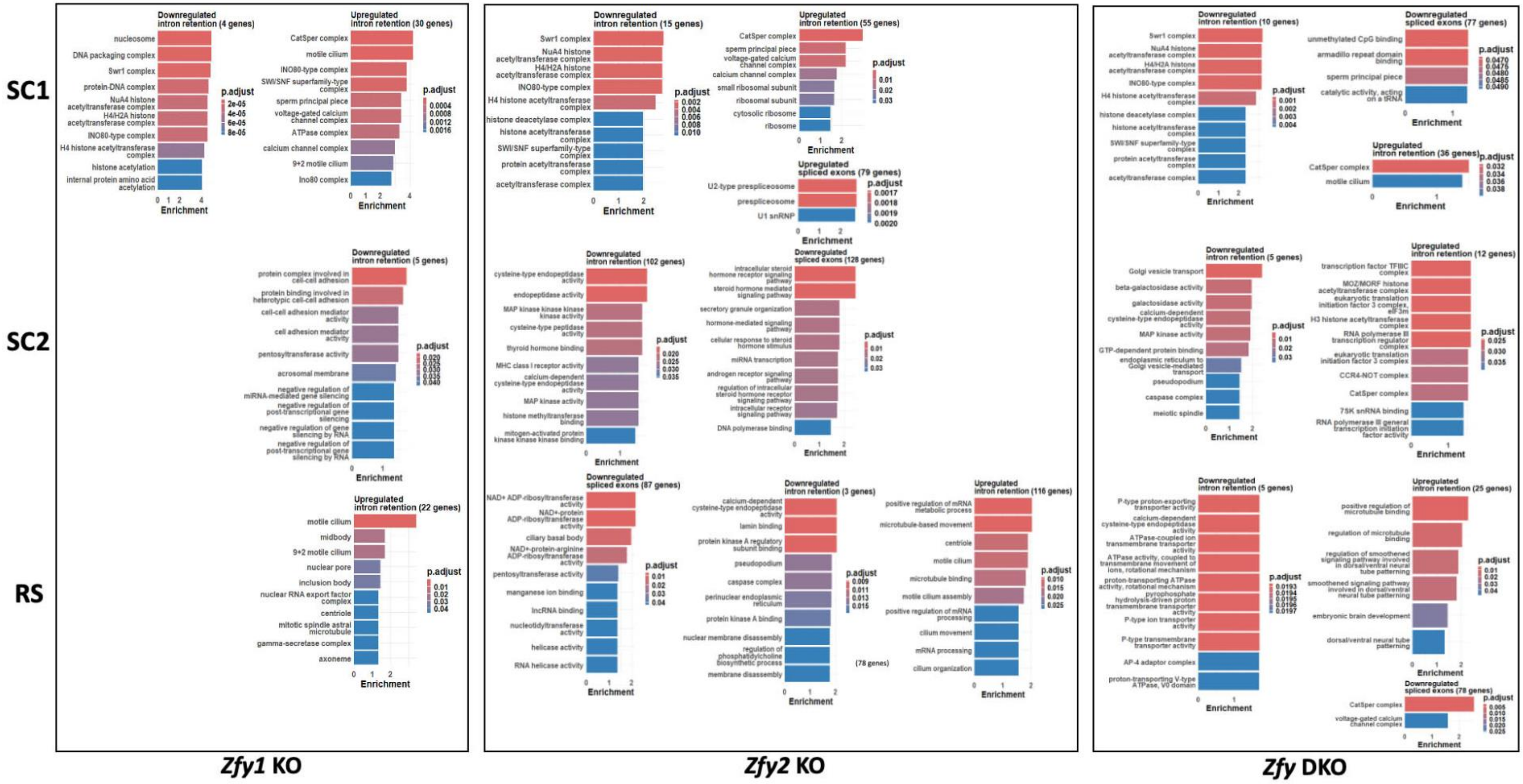

Figure S13. Enrichment analysis of genes subject to differential splicing. The analysis was performed for *Zfy1* KO, *Zfy2* KO, and *Zfy* DKO, against XY-wild type controls, across three germ cell types: primary spermatocytes (SC1), secondary spermatocytes (SC2), and round spermatids (RS). The numbers in brackets represent the number of genes in each category

These files are available as independently uploaded supplementary files:

Supplemental File S1: Enriched pathways identified by the GO analysis function of the HOMER tool. Tables are organized by genotype, cell type, upregulated vs downregulated, and database. 1KO = *Zfy1* KO, 2KO = *Zfy2* KO, DKO = *Zfy* DKO, SC1 = primary spermatocytes, SC2 = secondary spermatocytes, RS = round spermatid, BP = biological process, CC = cellular component, MF = molecular function, UP = upregulated, DOWN = downregulated, enrichment = p value, LogP = log of p value, genes in term = number genes in GO term, target genes in term = number of differentially expressed genes (DEGs) in term, fraction of targets in term = ratio of target genes in term to all genes in term, total target genes = total number of DEGs, total genes = total number of genes present in RNA-seq data. No enriched pathways were identified for *Zfy1* KO SC1 or SC2 cells. Supplemental Tables S2-3 and S6-7 contain the top 30 enriched pathways from *Zfy* DKO downregulated, *Zfy2* KO downregulated, *Zfy* DKO upregulated, and *Zfy2* KO upregulated, but all enriched pathways can be found in this file.

Supplemental File S2: Concordantly regulated genes in mouse germ cells and transfected HEK293 cells. The file contains three sheets, showing (1) concordant regulation of Consistently Activated Genes (CAGs; upregulated in ZFY knockin cells and downregulated in *Zfy* DKO) and Consistently Repressed Genes (CRGs; downregulated in ZFY knockin cells and upregulated in *Zfy* DKO), separate for three germ cell types from *Zfy* DKO; (2) CAGs pathway analysis and (3) CRG pathway analysis. The data are related to Fig. 8.

Supplementary Movie M1: Exemplary motility of sperm from XY control.

Supplementary Movie M2: Exemplary motility of sperm from *Zfy* DKO male.

Supplementary References

1. Yabuta, Y., Kurimoto, K., Ohinata, Y., Seki, Y. and Saitou, M. (2006) Gene expression dynamics during germline specification in mice identified by quantitative single-cell gene expression profiling. *Biol Reprod*, **75**, 705-716.
2. Moretti, C., Vaiman, D., Tores, F. and Cocquet, J. (2016) Expression and epigenomic landscape of the sex chromosomes in mouse post-meiotic male germ cells. *Epigenetics Chromatin*, **9**, 47.
3. Chen, Y., Zheng, Y., Gao, Y., Lin, Z., Yang, S., Wang, T., Wang, Q., Xie, N., Hua, R., Liu, M. *et al.* (2018) Single-cell RNA-seq uncovers dynamic processes and critical regulators in mouse spermatogenesis. *Cell Res*, **28**, 879-896.
