# Supplementary material for: Large-scale transcriptomic analyses reveal downstream target genes of ZFY1 and ZFY2 transcription factors in male germ cells
Source: Cell Death Differ. 2025 Aug 27;33(2):392–410. doi: 10.1038/s41418-025-01569-6 (PMC12881418; doi:10.1038/s41418-025-01569-6)

Original Data

Protamine content in sperm from *Zfy* KO males – untrimmed gel and blot.

Coomassie stained photo of untrimmed acid urea polyacrylamide gel electrophoresis (AU-PAGE) of cauda epididymal sperm basic nuclear protein extracts from XY, *Zfy1* KO, *Zfy2* KO and *Zfy* DKO males (relates to Fig. 4Ai).

Untrimmed photo of acidic Western blot using anti-PRM2 antibody of cauda epididymal sperm basic nuclear protein extracts from XY and *Zfy* DKO males (relates to Fig. 4Aii).

These images are also shown as Fig. S10.

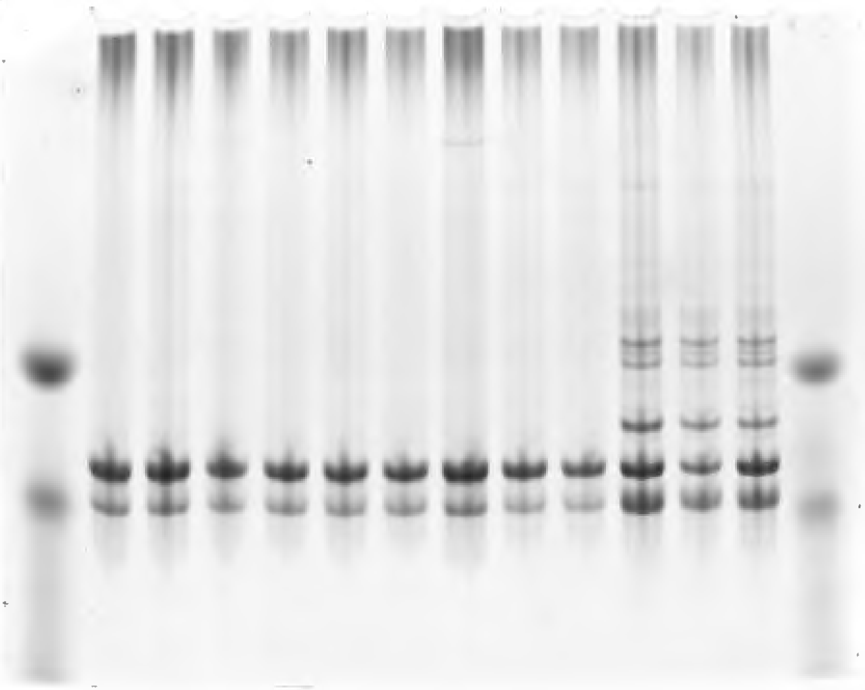

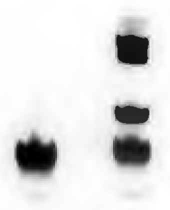

Supplement: Supplementary file 2 — Original Data [file 41418_2025_1569_MOESM2_ESM.pdf]
